# A big data approach to macrofaunal baseline assessment, monitoring and sustainable exploitation of the seabed

**DOI:** 10.1038/s41598-017-11377-9

**Published:** 2017-09-29

**Authors:** K. M. Cooper, J. Barry

**Affiliations:** Centre for Environment, Fisheries and Aquaculture Science, Lowestoft Laboratory, Pakefield Road, Lowestoft, Suffolk, NR33 0HT United Kingdom

## Abstract

In this study we produce a standardised dataset for benthic macrofauna and sediments through integration of data (33,198 samples) from 777 grab surveys. The resulting dataset is used to identify spatial and temporal patterns in faunal distribution around the UK, and the role of sediment composition and other explanatory variables in determining such patterns. We show how insight into natural variability afforded by the dataset can be used to improve the sustainability of activities which affect sediment composition, by identifying conditions which should remain favourable for faunal recolonisation. Other big data applications and uses of the dataset are discussed.

## Introduction

In common with many parts of the world, the UK’s seas are increasingly subject to pressure from a range of anthropogenic activities^1,2^. These activities include, *inter alia*, fishing, oil and gas operations, aggregate dredging, renewable energy generation, dredging of ports and harbours, and installation of cables and pipelines. To ensure maintenance of a ‘healthy’ marine environment^3^, it is essential that these activities are, as far as possible, carried out in an environmentally sustainable way. Whilst the focus of this study is on marine aggregate dredging and faunal-sediment relationships, findings may have relevance for other activities affecting seabed sediment composition.

**Figure 1:**
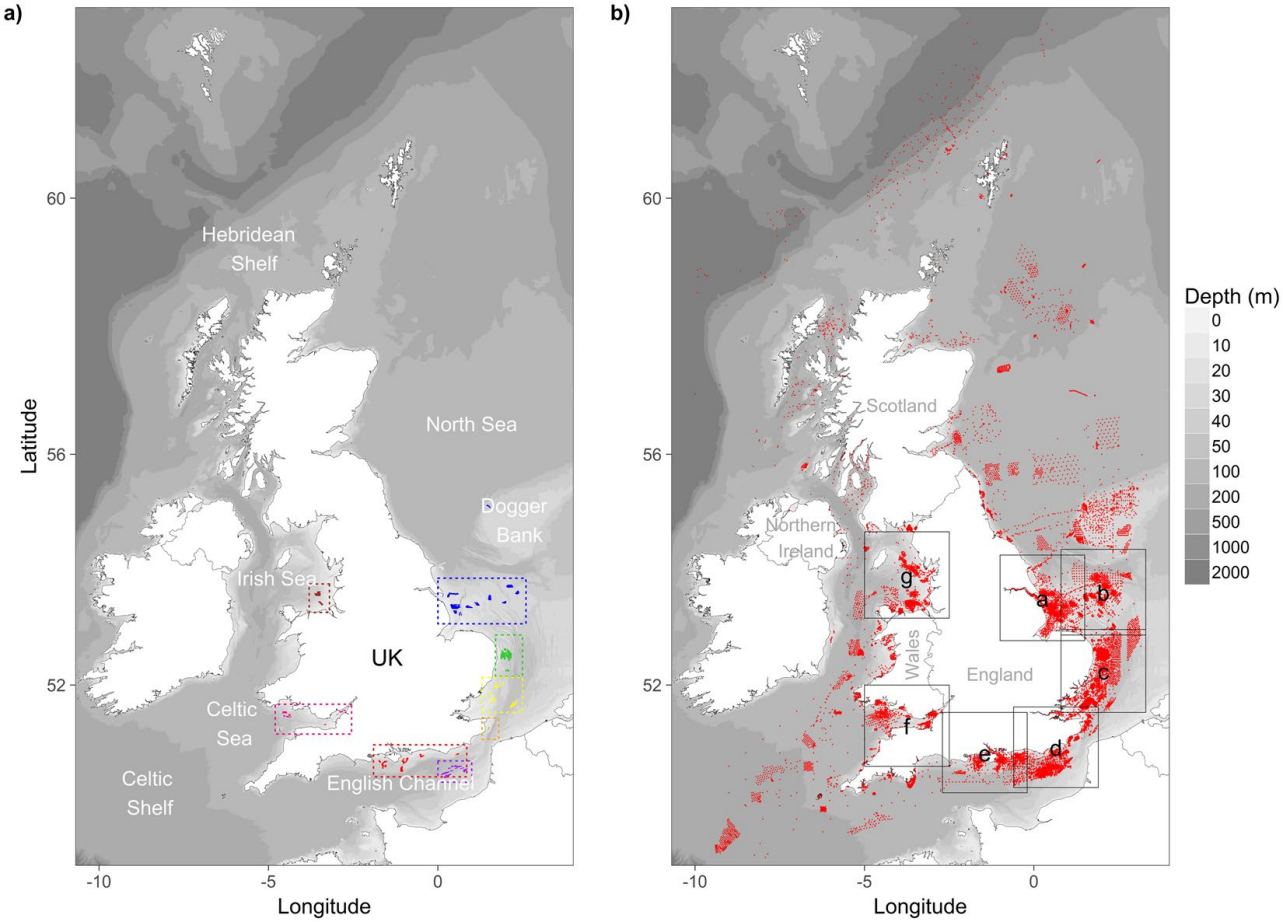
(**a**) Study area showing locations of aggregate dredging interest (blue - Humber dredging region, green - Anglian dredging region, yellow - Thames dredging region, orange - Goodwin Sands dredging licence, purple - eastern English Channel dredging region, red - South Coast dredging region, dark pink - Bristol Channel/Severn Estuary dredging region, brown - North-West dredging region). (**b**) Sample locations and extent of submaps used in Fig. 7. Underlying bathymetry is from the 2015 updated version of Defra’s Digital Elevation Model (DEM)^54^. Maps created in RStudio (version 1.0.143, https://www.rstudio.com/).

The UK marine aggregate dredging industry produces sand and gravel (aggregate) from licensed extraction areas located around the coast of England and Wales (Fig. 1a), with material used for construction, fill and coastal defence^4^. The process of aggregate dredging can create some localised environmental impacts (e.g. changes in seabed topography^5,6^, alterations to sediment composition^6–13^ and loss of benthic fauna^6,14,15^), although these vary considerably^16,17^. In recognition of the likely impacts, the aggregate industry’s operations are subject to Environmental Impact Assessment (EIA), and, where activities are subsequently licensed, to environmental monitoring over the course of the dredging permission.

Typically, this environmental monitoring has focused on assessing impacts of ongoing dredging, with considerable attention given to the invertebrate seabed assemblages or ‘benthos’. Whilst of interest, impacts to the benthos (e.g. reductions in faunal diversity and abundance) are increasingly predictable as the number of scientific studies increases^17^. What the monitoring has not explained to date, however, is what is likely to happen to impacted sites after cessation of dredging^11^? For example, will it be possible for the original faunal assemblage type to return? Whilst this issue of recoverability is surely the most important question for sustainability, the number of recovery studies remains limited^10,12,13,18–22^ due to the time-consuming and expensive nature of the work.

Expert judgements are made regarding the faunal recovery potential of aggregate extraction sites. However, predictions are difficult given the large number of factors involved (e.g. sensitivity of faunal communities to disturbance and changes in sediment composition^11^, nature of the local environment and its capacity to recover from physical disturbance^13,23^). Where recovery does not proceed in line with expectations then, theoretically, options for active seabed restoration can be considered^24^. However, whilst technically feasible, such options are likely to be expensive^25^ and outcomes uncertain^26,27^. For this reason, the existence of unacceptable residual impacts should, arguably, signify a failure of the monitoring and management process^17,25^.

In recognition of these challenges, an alternative approach to monitoring has recently been suggested^28,29^. This new approach seeks to ensure the return of the pre-impacted faunal assemblage type, thus preserving the ecosystem functioning of the seabed^30^. This is achieved by maintaining the associated habitat (sediment composition) within certain limits. These habitat limits are determined by the range of conditions seen for the particular assemblage in the wider environment. Following the successful testing of this approach at site specific^28^ and regional^29^ scales, a decision was taken by regulators, industry and other stakeholders to adopt it across all regions of aggregate dredging in the UK (see Fig. 1a). The newly termed ‘Regional Seabed Monitoring Programme’ (RSMP)^31^ is designed to improve environmental protection by making it clear when unacceptable changes in sediment composition are occurring, allowing for early management intervention. It is also expected to significantly reduce the costs of monitoring for the aggregates industry.

To allow for implementation of the RSMP, it is necessary to: (i) produce a baseline assessment of the UK’s macrobenthic infauna, with a particular focus around sites and regions of marine aggregate dredging, (ii) identify the range of sediment composition found in association with the different baseline faunal assemblages, and (iii) develop a method for assessing the likely ecological significance of anthropogenically-induced changes in sediment composition. These objectives are achieved using a dataset comprising of new and existing data belonging to government and industry sources.

## Results

### Faunal data analysis

#### Univariate indices

**Figure 2:**
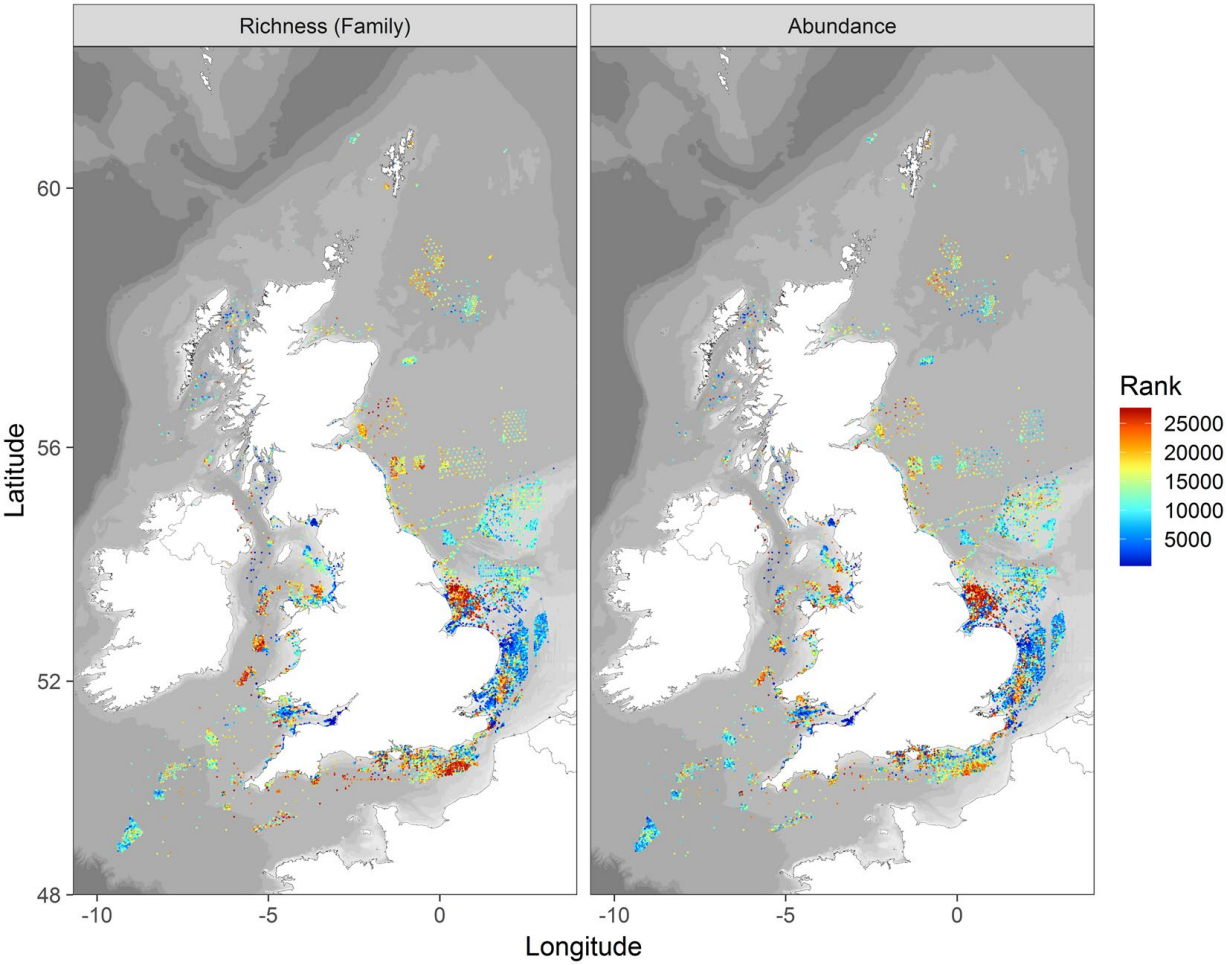
Heat maps based on a ranked ordering of samples for taxon richness and abundance per 0.1 m^2^ (see Fig. 7 for more detail of the aggregate producing regions). Maps created in RStudio (version 1.0.143, https://www.rstudio.com/).

Maps of taxon (family) richness (hereafter referred to as taxon richness) and total abundance reveal a complicated picture, although some patterns are discernible (Fig. 2). For instance, within the North Sea there is an underlying trend of increasing values for both measures with increasing latitude. A similar trend is observed from the Celtic Shelf to the southern Irish Sea. Within the English Channel values are generally high, although there is a clear transition to lower values at the eastern end. Patterns within the Irish Sea and along the west coast of Scotland are less clear because of the lower numbers of samples in these areas. Hotspots of diversity (in terms of taxon richness) can be found in several areas. Many of these hotspots are consistent with underlying trends (e.g. mid English Channel and mid Irish Sea), whilst others appear in marked contrast (e.g. Humber, Outer Thames). Within the Humber hotspot in particular, samples with very high values of both measures lie adjacent to those with very low values.

#### Community analysis

**Figure 3:**
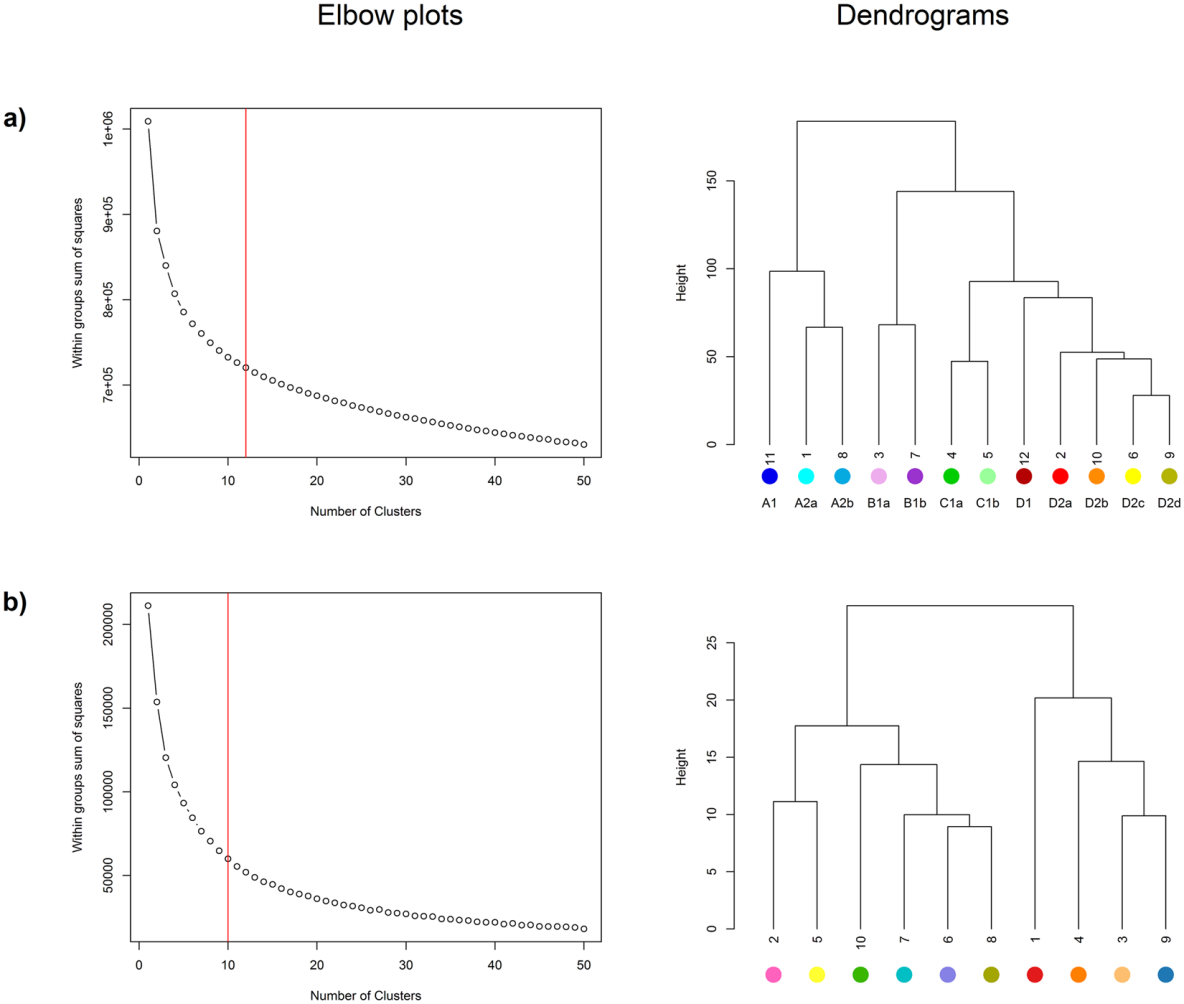
Elbow plots and dendrograms associated with a k-means clustering of (**a**) macrofaunal and (**b**) physical variables. The labels and colours used in the faunal dendrogram were chosen to reflect the relationship (similarity/dissimilarity) between the different cluster groups.

The elbow plot relating to the faunal data did not suggest an obvious number of groups for k-means clustering (Fig. 3a). We chose a clustering solution based on 12 groups, as this number coincided with a slight levelling out of the plot and explained >70% of the inherent variability. Opting for a lower rather than a higher number of cluster groups also served to increase the number of sample replicates for sediment analysis (see below).

**Figure 4:**
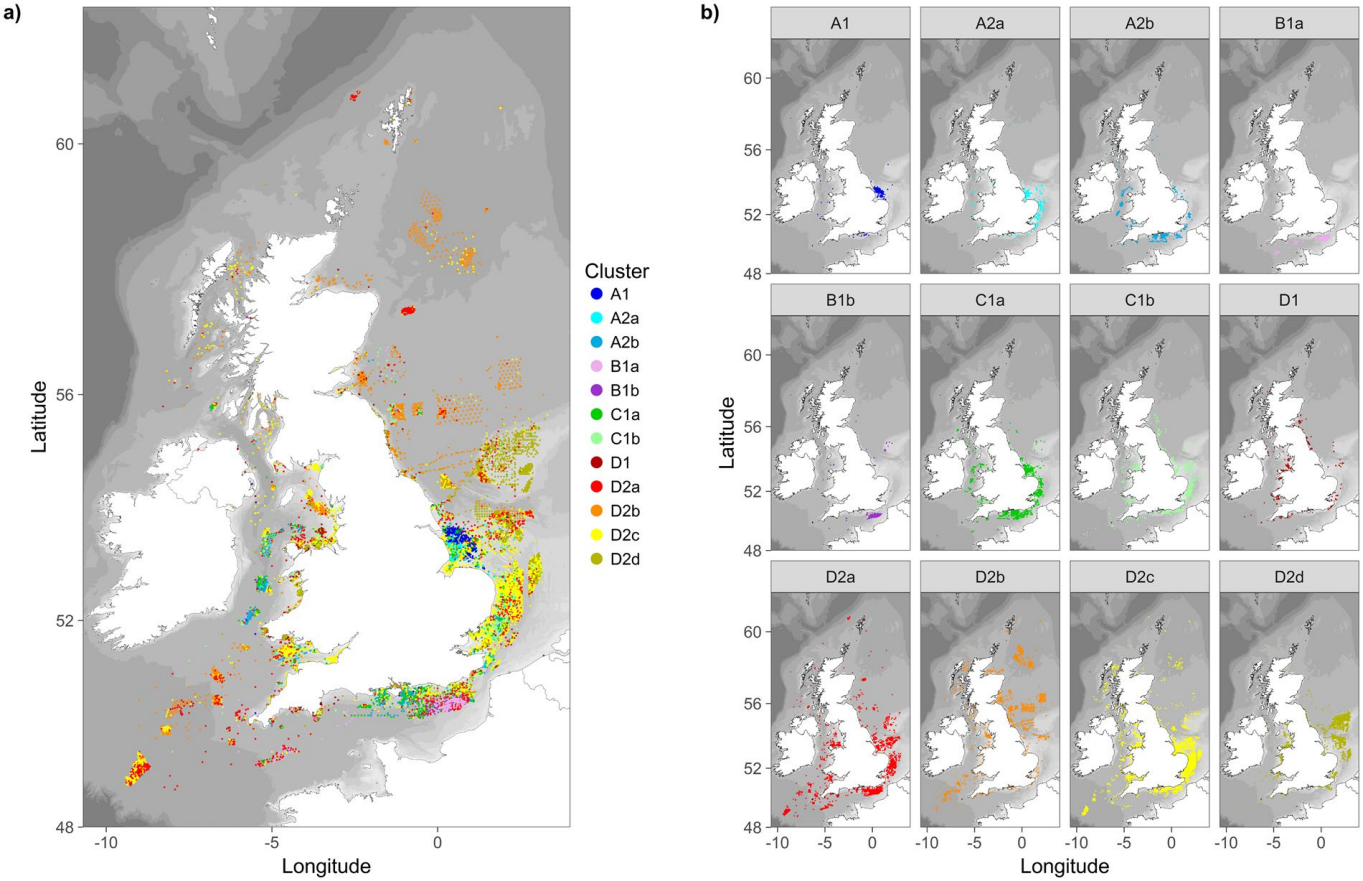
Spatial distribution of macrofaunal assemblages with all samples (**a**) and by individual cluster group (**b**). Assemblage groups are based on a k-means clustering of fourth-root transformed macrofaunal abundance data (colonials included). Maps created in RStudio (version 1.0.143, https://www.rstudio.com/).

The dendrogram reveals four broad groupings in the data, within which there are further subdivisions (Fig. 3a). Clear differences can be seen in the spatial distribution of faunal assemblages at a UK level (Fig. 4). For instance, some are spatially restricted (e.g. A1, A2a, B1a and B1b), whilst others are more widely distributed (e.g. C1a, C1b, D2a, D2b, D2c and D2d). Assemblage A1 is found almost exclusively off the Humber. A2a is predominately found in the southern North Sea, whilst being almost entirely absent from the northern North Sea and Celtic Sea. The main areas for assemblage A2b occur in the mid English Channel and mid Irish Sea, although it is also found in the outer Thames estuary and in other isolated patches. Assemblages B1a and B1b occur mainly in the eastern English Channel, with isolated occurrences elsewhere. C1a is found from the Humber region round to Lyme Bay, and also in the Bristol Channel and mid Irish Sea; isolated patches occur elsewhere. C1b has a widespread distribution, with notable patches in the southern North sea and coastal areas of the eastern English Channel. D1 has a largely coastal distribution throughout UK waters, although it is also found in some offshore areas of the southern North Sea. D2a has a widespread distribution, although it is notably absent from the more sandy areas of the Severn Estuary. Assemblage D2b is common in the deeper waters of the northern North Sea, the Celtic Sea and off the west coast of Scotland; with isolated occurrences elsewhere. Assemblage D2c is commonly found off the east coast, eastern end of the English Channel, Celtic Sea and Bristol Channel/Severn Estuary. D2d is found in offshore areas of the southern North Sea and on the Dogger Bank. Elsewhere, D2d can be found in coastal areas along the west coast of England and Wales, off the North East of England, in the eastern English Channel and in other isolated patches.

Results of a SIMPER analysis showed clear differences in the number of characterising taxa between the faunal assemblage groups (Table 1). For example, groups D2a, D2b, D2c and D2d were characterised by low numbers of taxa, whilst groups A1, A2a, A2b and B1a, had relatively higher numbers of characterising taxa. Polychaetes were the dominant faunal group across all assemblages, with bivalve molluscs being a feature of groups A1, A2a, B1b, D1, D2b and D2d. Epifaunal taxa such as bryozoans were characteristic of groups A1, A2b and B1a. Several taxa (e.g. *Spionidae*) characterised multiple assemblage groups, whilst others (e.g. the bivalve mollusc, *Glycymerididae*) were characteristic of only one assemblage, B1b. The highest and lowest mean numbers of taxa and abundance were associated with faunal groups A1 and D2c respectively.

**Table 1:** Biological characteristics of the macrofaunal assemblages identified through a k-means clustering of macrofaunal data (colonials included, forth-root transformation).

Cluster		Characteristic taxa	Richness (Family)				Abundance				*n*
			Max.	Min.	Mean		Max.	Min.	Mean		
	A1	*Balanidae* (C), *Styelidae* (AT), *Spionidae* (P), *Terebellidae* (P), *Syllidae* (P), *Porcellanidae* (DC), *Polynoidae* (P), *Sabellariidae* (P), *Capitellidae* (P), *Serpulidae* (P), *Nemertea* (N), *Cirratulidae* (P), *Mytilidae* (BM), *Phyllodocidae* (P), *Nematoda* (Ne), *Alcyonidiidae* (B), *Galatheidae* (DC), *Romancheinidae* (B), *Pholoidae* (P), *Amphiuridae* (E), *Electridae* (B)	120	35	70 (±14)	↑	6738	186	1122 (±789)	↑	366
	A2a	*Sabellariidae* (P), *Spionidae* (P), *Polynoidae* (P), *Terebellidae* (P), *Nemertea* (N), *Phyllodocidae* (P), *Lumbrineridae* (P), *Pholoidae* (P), *Cirratulidae* (P), *Capitellidae* (P), *Syllidae* (P), *Semelidae* (BM), *Porcellanidae* (DC)	106	21	52 (±14)	↑	11821	104	972 (±1151)	↑	707
	A2b	*Syllidae* (P), *Serpulidae* (P), *Terebellidae* (P), *Spionidae* (P), *Sabellariidae* (P), *Polynoidae* (P), *Capitellidae* (P), *Lumbrineridae* (P), *Cirratulidae* (P), *Amphiuridae* (E), *Porcellanidae* (DC), *Maldanidae* (P), *Styelidae* (AT), *Phyllodocidae* (P), *Nemertea* (N), *Sabellidae* (P), *Romancheinidae* (B)	104	29	58 (±12)	↑	3458	73	366 (±265)	↑	1191
	B1a	*Spionidae* (P), *Serpulidae* (P), *Syllidae* (P), *Glyceridae* (P), *Galatheidae* (DC), *Phyllodocidae* (P), *Terebellidae* (P), *Romancheinidae* (B), *Amphiuridae* (E), *Bitectiporidae* (B), *Polynoidae* (P), *Nemertea* (N), *Capitellidae* (P), *Scalibregmatidae* (P), *Phidoloporidae* (B), *Chorizoporidae* (B), *Microporellidae* (B), *Eunicidae* (P), *Cirratulidae* (P), *Adeonidae* (B)	117	35	64 (±12)	↑	879	58	227 (±109)	→	1099
	B1b	*Spionidae* (P), *Syllidae* (P), *Serpulidae* (P), *Terebellidae* (P), *Phyllodocidae* (P), *Capitellidae* (P), *Glyceridae* (P), *Nemertea* (N), *Polynoidae* (P), *Galatheidae* (DC), *Amphiuridae* (E), *Cirratulidae* (P), *Glycymerididae* (BM)	86	24	44 (±9)	→	2311	56	206 (±121)	→	1687
	C1a	*Spionidae* (P), *Terebellidae* (P), *Serpulidae* (P), *Syllidae* (P), *Capitellidae* (P), *Cirratulidae* (P), *Lumbrineridae* (P), *Sabellariidae* (P), *Nemertea* (N), *Glyceridae* (P)	66	10	31 (±9)	→	6056	19	153 (±252)	→	2462
	C1b	*Spionidae* (P), *Capitellidae* (P), *Terebellidae* (P), *Nemertea* (N), *Lumbrineridae* (P), *Cirratulidae* (P), *Glyceridae* (P), *Ampeliscidae* (A), *Phyllodocidae* (P), *Polynoidae* (P), *Scalibregmatidae* (P), *Pholoidae* (P), *Serpulidae* (P)	195	18	44 (±11)	→	4611	57	243 (±187)	→	1850
	D1	*Spionidae* (P), *Montacutidae* (BM), *Semelidae* (BM), *Nephtyidae* (P), *Capitellidae* (P), *Cirratulidae* (P), *Amphiuridae* (E), *Oweniidae* (P), *Nemertea* (N), *Pholoidae* (P), *Nuculidae* (BM)	107	12	40 (±11)	→	7607	113	637 (±665)	↑	962
	D2a	*Spionidae* (P), *Glyceridae* (P), *Nemertea* (N), *Terebellidae* (P), *Capitellidae* (P), *Phyllodocidae* (P)	56	6	24 (±8)	↓	4471	13	91 (±126)	↓	3288
	D2b	*Spionidae* (P), *Amphiuridae* (E), *Nephtyidae* (P), *Lumbrineridae* (P), *Oweniidae* (P), *Cirratulidae* (P), *Capitellidae* (P), *Nemertea* (N), *Semelidae* (BM), *Ampharetidae* (P)	56	9	27 (±8)	↓	4023	24	139 (±126)	↓	2631
	D2c	*Nephtyidae* (P), *Spionidae* (P), *Opheliidae* (P)	32	1	8 (±5)	↓	2098	1	26 (±61)	↓	8286
	D2d	*Spionidae* (P), *Bathyporeiidae* (A), *Nephtyidae* (P), *Magelonidae* (P), *Tellinidae* (BM)	45	4	19 (±6)	↓	5563	13	100 (±183)	↓	2903

Characterising species were identified through a SIMPER analysis and include taxa up to a total of 50% contribution. Letters in parenthesis identify the higher level taxonomic group: Amphipod crustacean (A), Ascidian tunicate (AT), Broyzoa (B), Bivalve Mollusc (BM), Crustacean (C), Decapod Crustacean (DC), Echinoderm (E), Polychaete (P), Phoronida (Ph), Nematoda (Ne). Values for Richness and Abundance are means and standard deviations. Arrows indicate the relative size of a value (↑ - High, ↓- Low, →- Medium).

#### Temporal assessment

**Figure 5:**
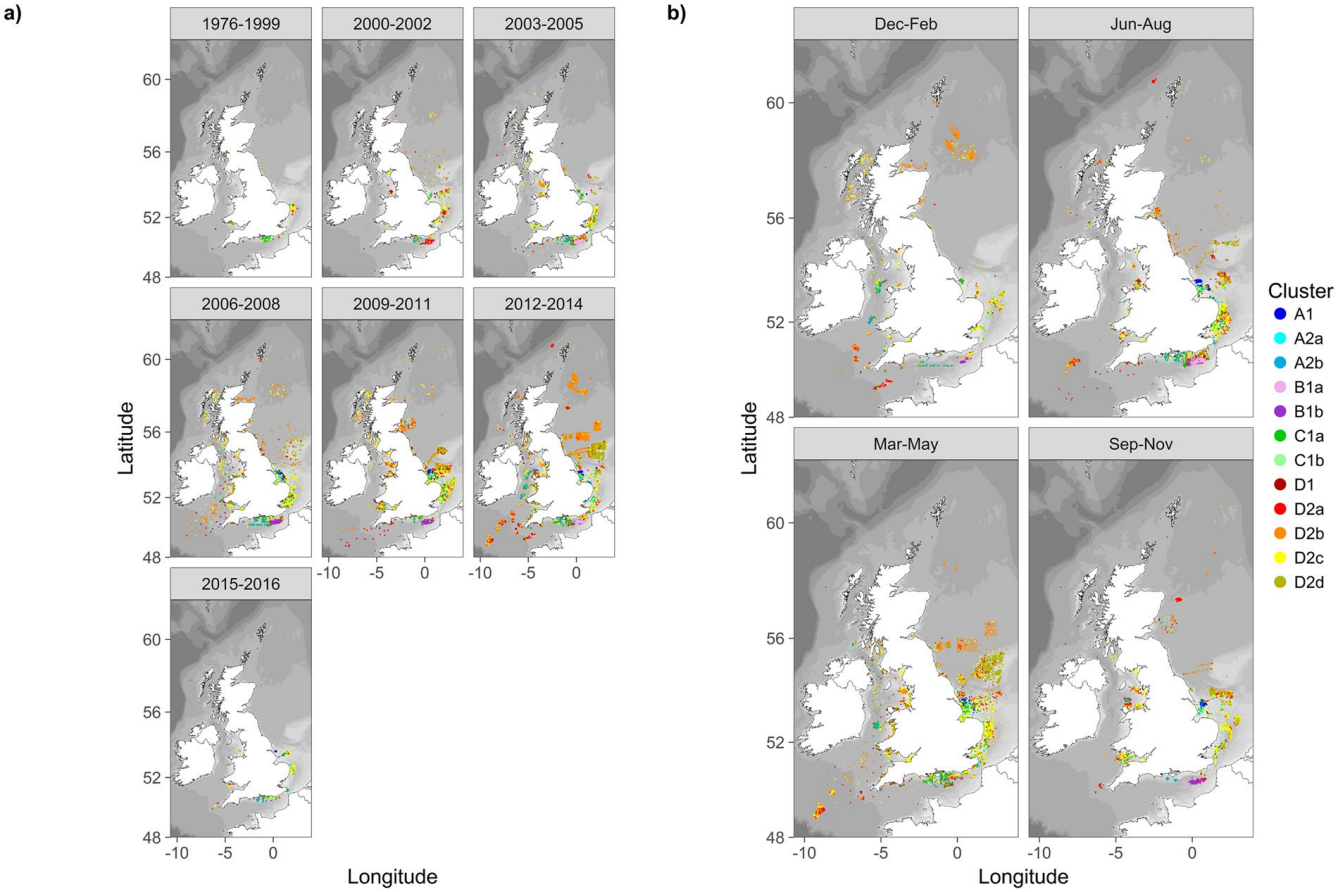
Faunal cluster distribution by year (**a**) and season (**b**). Maps created in RStudio (version 1.0.143, https://www.rstudio.com/).

Plots of faunal cluster identity by year (Fig. 5a) and season (Fig. 5b) showed broadly consistent spatial patterns. For example, assemblages A1/C1a, D2b, D2c, D2d and are frequently found, respectively, off the Humber estuary, in the deeper waters of the northern North Sea, off East Anglia, and on the Dogger Bank. This suggests that faunal assemblages, at the level of family, are largely stable though time. Within the eastern English Channel dredging region, the apparent switch from assemblage B1a to B1b (Fig. 5a) can be explained by the omission of colonial taxa from surveys undertaken between 2008 and 2013.

#### Explaining patterns in faunal distribution

Results of the *best* analysis identified 3 variables: AvCur, Mud and Sand as ‘best explaining’ the patterns in the macrofaunal data (Table 2). Despite the moderate correlation between the underlying resemblance matrices (ρ = 0.4), results from *adonis* showed that these predictors only accounted for a total of 13.1% of the total variability (AvCur = 6.1%, Mud = 3.0% and Sand = 4.0%); all were statistically significant (*p* < 0.01).

**Table 2:** Results of a *best* analysis identifying the subset of environmental variables which are most correlated with the macrofaunal data.

Variable(s)	Size	Correlation (ρ)
Sand	1	0.2998
AvCur, Sand	2	0.3842
**AvCur**, **Mud**, **Sand**	**3**	**0**.**4207**
Lat, AvCur, Mud, Sand	4	0.4161
Lat, Depth, AvCur, Mud, Sand	5	0.4089
Lat, SPM, Depth, AvCur, Mud, Sand	6	0.3973
Lat, SPM, Depth, WOV, AvCur, Mud, Sand	7	0.3847
Lat, Sal, SPM, Depth, WOV, AvCur, Mud, Sand	8	0.3704

**Figure 6:**
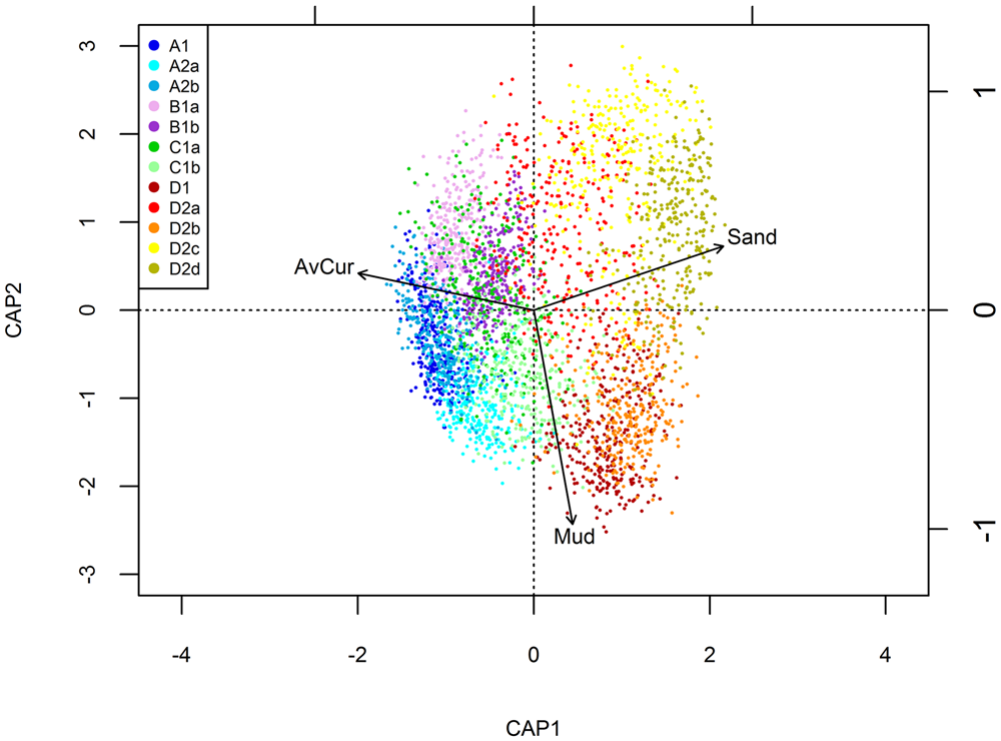
Distance-based redundancy analysis (dbRDA) ordination showing sampling sites (coloured by faunal assemblage group) and vectors for the main environmental predictor variables. Note that the variables Sand and AvCur were highly correlated with Gravel and Stress respectively.

When considered together, the dbRDA ordination (Fig. 6) and heat maps for individual environmental variables (Supplementary Fig. S1) provide some insight into the spatial distribution of faunal assemblages (Fig. 4b). For example, groups D1 and D2b are found in areas of high mud content with weaker currents; D2c and D2d dominate in areas of high sand content; A1, A2b, B1a, B1b and C1a and are all found in areas with high gravel content and strong currents; C1b, A2a and D2a are found in areas of more mixed sediment.

#### Faunal distribution within areas of aggregate industry interest

**Figure 7:**
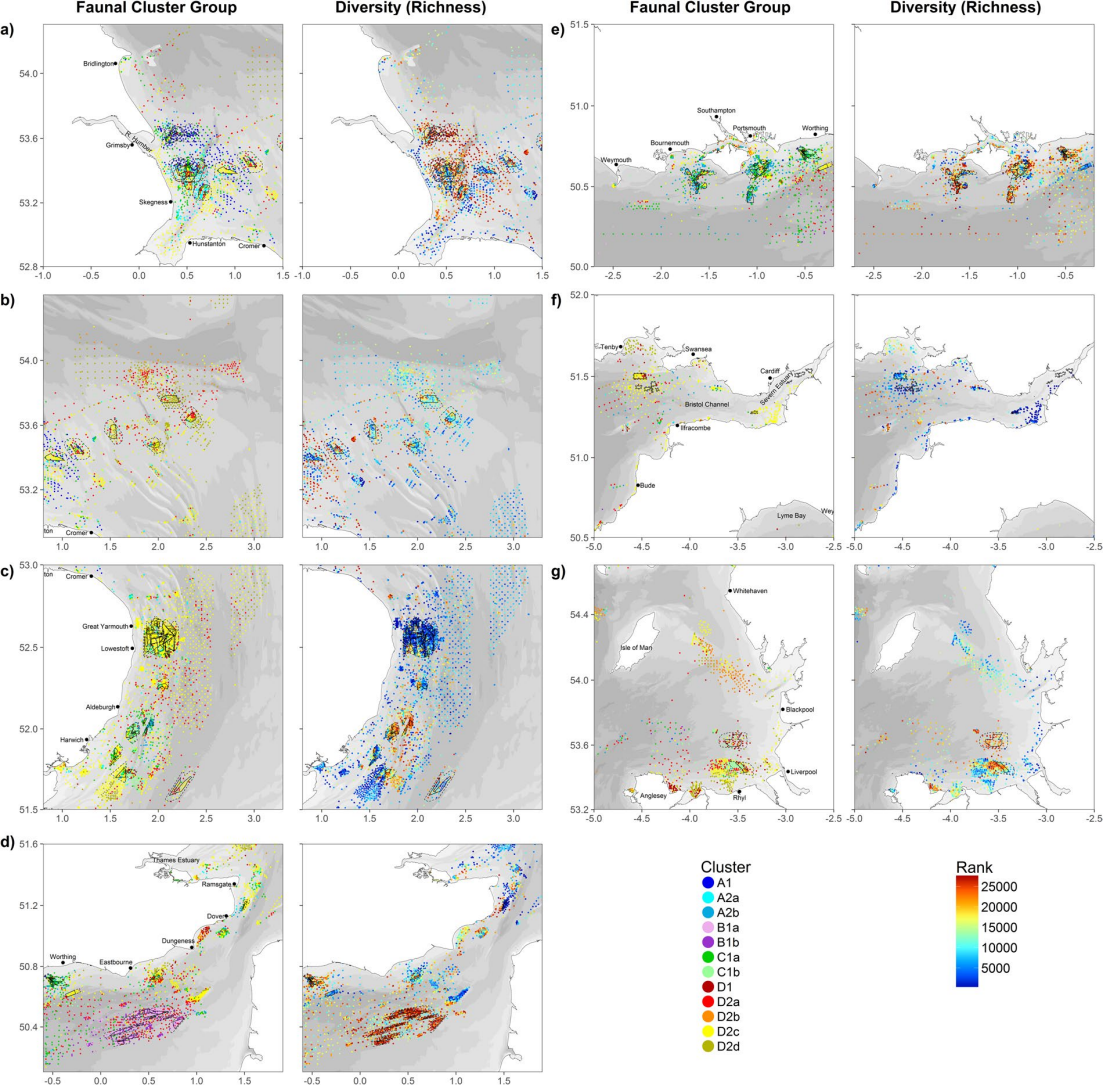
Faunal cluster group and diversity (taxon Richness) for samples by sub region (see Fig. 1b for submap extents). Areas of aggregate dredging interest (licensed and application areas) shown as solid black lines, whilst areas of potential secondary effect are shown as dashed black lines. Maps created in RStudio (version 1.0.143, https://www.rstudio.com/).

Maps showing the faunal cluster identity of stations within aggregate extraction sites, their zones of potential secondary effect, and the wider region (Fig. 7) fulfil the first objective of this study: to produce a baseline assessment of benthic macrofauna, with a particular focus around sites and regions of marine aggregate dredging. As seen in these plots, the nature of faunal assemblages differs between extraction areas, with some supporting only a single assemblage (e.g. Figure 7c,f), whilst others support multiple groups (e.g. Figure 7b). With the exception of assemblage D2b, all groups are represented within extraction areas. In addition, taxon richness also shows wide variation across extraction sites, both within and between regions. For example, some sites support very spare assemblages (Fig. 7c,f), whilst others support much richer assemblages (Fig. 7a,d,e).

### Faunal-sediment relationships

In this section we examine the composition of sediments found in association with different faunal cluster groups - first using all the data (i.e. irrespective of sample location) and then within different physical cluster regions to control for the influence of other variables. We also explore whether reductions in the proportion of gravel could lead to declines in taxon richness and total abundance.

#### Identification of physical cluster groups

**Figure 8:**
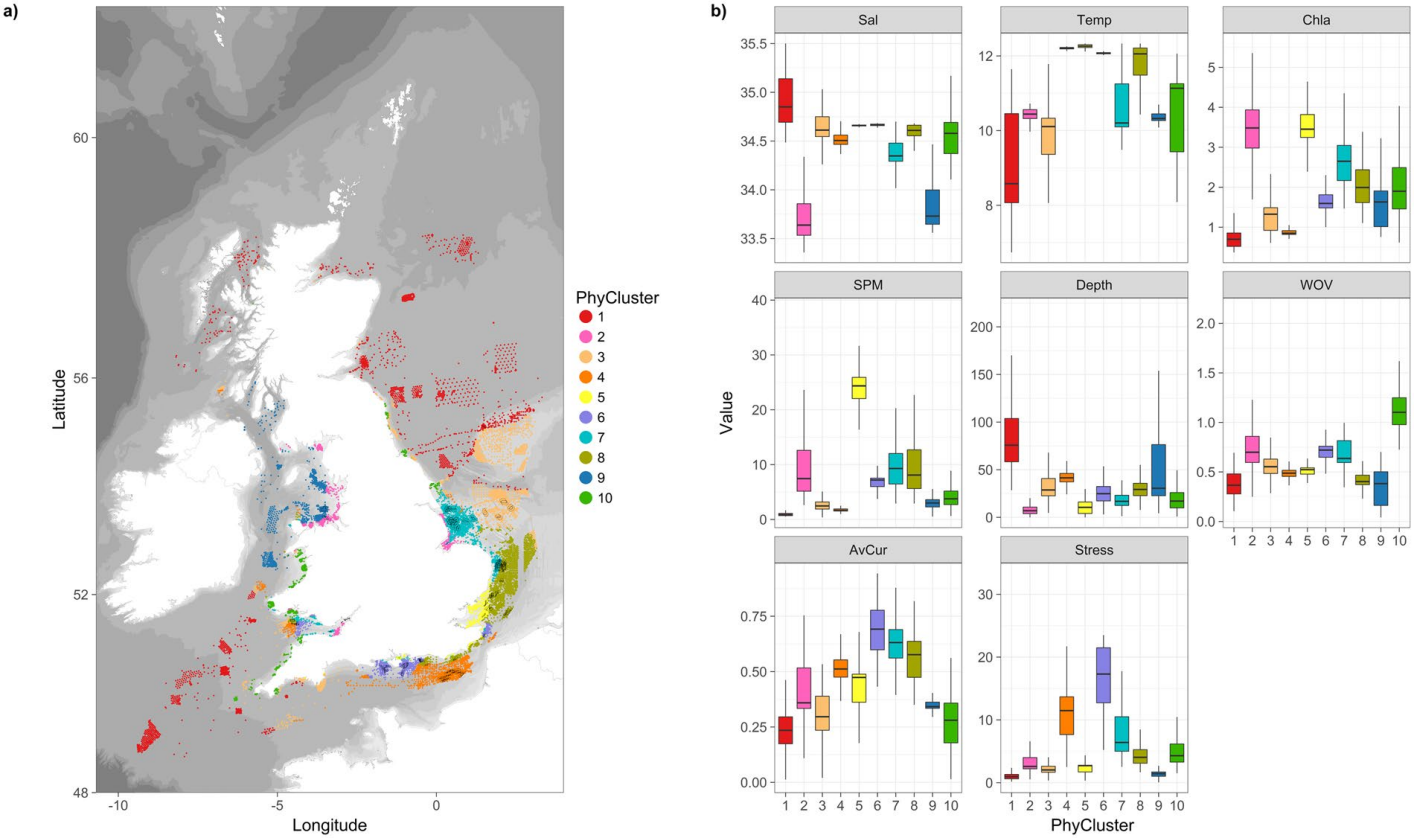
(**a**) Physical cluster group identity for individual sample stations. Analysis based on a k-means clustering of non-sediment environmental variables for: Sal, Temp, Chl a, SPM, Depth, WOV, AvCur and Stress (see Table 5 for details). (**b**) Box and whisker plots for physical variables by physical cluster group. Map created in RStudio (version 1.0.143, https://www.rstudio.com/).

Based on the output of an elbow plot (Fig. 3b), k-means clustering was used to partition samples into 10 environmental cluster groups (Fig. 8a). Box and whisker plots for environmental variable by physical cluster group (Fig. 8b) provide insight into the different environmental conditions (not including sediments) associated with each group. For example, sites belonging to cluster group 6, located around the Isle of Wight, are associated with strong average currents and high bed stress.

#### Sediment composition by faunal and faunal-physical cluster groups

**Figure 9:**
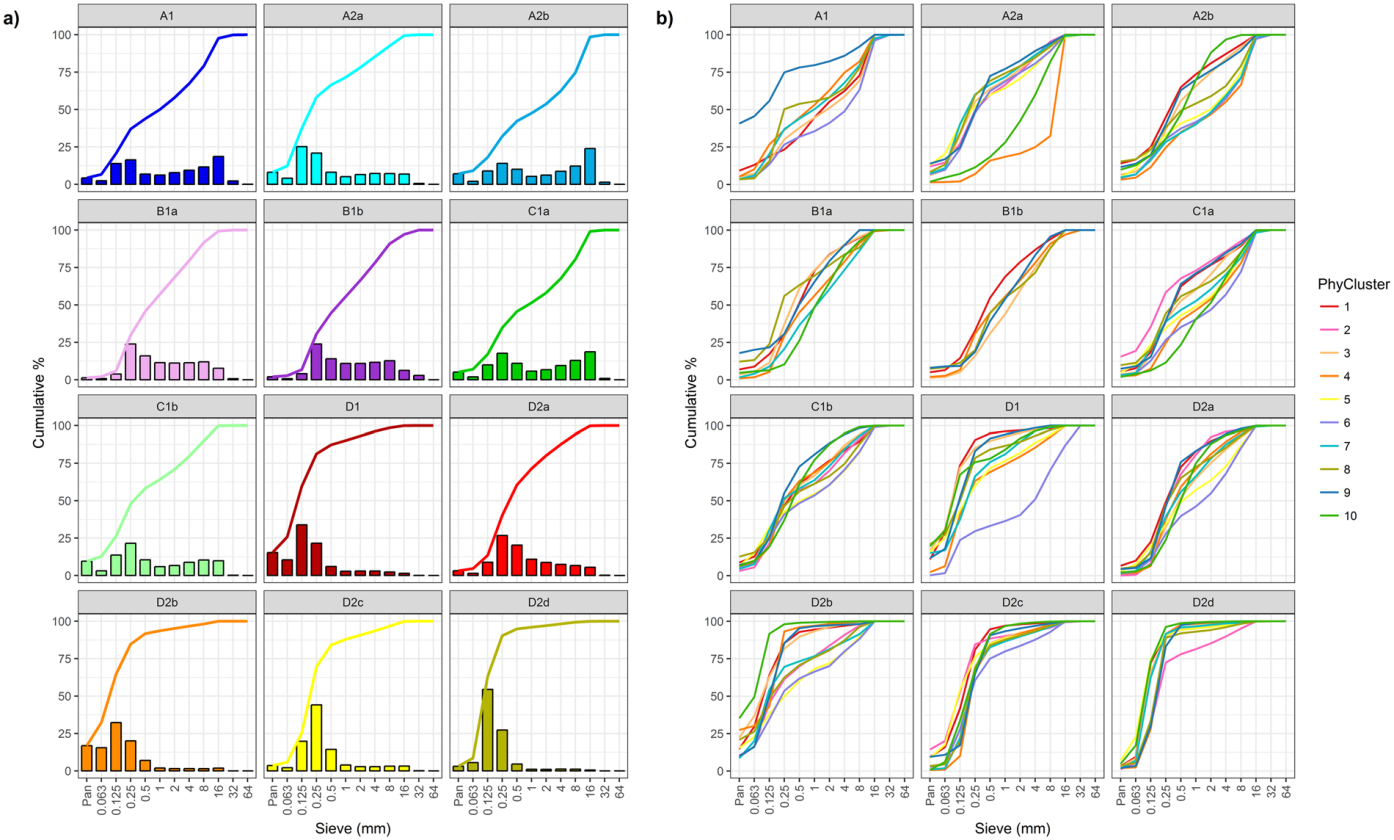
(**a**) Mean cumulative sediment distribution plots, with accompanying histogram, for each faunal cluster group. (**b**) Mean cumulative sediment distribution plots by physical cluster group, faceted by faunal cluster group.

Examination of cumulative sediment distribution plots reveals some clear differences in the mean sediment composition of samples from the different faunal cluster groups (Fig. 9a). The most obvious separation is between samples with a significant proportion of gravel (groups A, B and C), versus those mainly dominated by sand (group D). Sand-dominated groups are split between those with higher (D1 and D2b) and lower (D2a, D2c and D2d) percentages of mud. Variability in sediment composition, as revealed by values of MVDISP, was highest for samples belonging to D2c and lowest for samples belonging to B1a and B1b (Table 3).

**Table 3:** Mean percentage composition of sediments by Wentworth size class for each faunal cluster group.

Bio Cluster		*n*	% Mud	% Sand				% Gravel				Description	MVDISP
			Sum	fS	mS	cS	Sum	fG	mG	cG	Sum		
	A1	290	4	16	16	13	46	17	12	21	50	v slightly muddy sandy gravel	0.74
	A2a	458	8	29	21	13	63	14	7	7	28	slightly muddy gravelly sand	0.73
	A2b	731	7	11	14	15	40	15	12	25	52	slightly muddy sandy gravel	0.81
	B1a	1010	1	5	24	27	56	23	12	8	43	v slightly muddy gravelly sand	0.44
	B1b	774	2	5	24	25	54	23	13	9	44	v slightly muddy gravelly sand	0.44
	C1a	1327	5	12	18	17	46	16	13	20	49	slightly muddy sandy gravel	0.90
	C1b	1018	10	17	22	16	55	16	10	10	36	slightly muddy gravelly sand	0.80
	D1	145	15	44	22	9	75	6	2	2	10	slightly gravelly slightly muddy sand	0.99
	D2a	1522	3	10	27	31	68	16	7	6	29	v slightly muddy gravelly sand	0.94
	D2b	652	17	48	20	9	77	3	1	2	6	slightly gravelly slightly muddy sand	1.05
	D2c	3485	4	22	44	18	84	6	3	3	12	v slightly muddy slightly gravelly sand	1.15
	D2d	1014	3	60	27	6	93	2	1	1	4	v slightly gravelly v slightly muddy sand	0.82

Sediment descriptions are in accordance with Blott and Pye (2011)^55^. Number of samples is shown by column *n*. Variability in sediment composition is indicated by the Multivariate Index of Dispersion (MVDISP).

Cumulative sediment distribution plots for each faunal-physical cluster group show a high degree of similarity, but also some differences (Fig. 9b). In most cases the more extreme values can be explained by a low number of replicates (e.g. A1_9, A2a_4, A2a_10, D1_6). Details of the mean sediment composition for each faunal-physical cluster group are shown in Supplementary Table S2.

#### Relationship between gravel and taxon richness/total abundance

**Figure 10:**
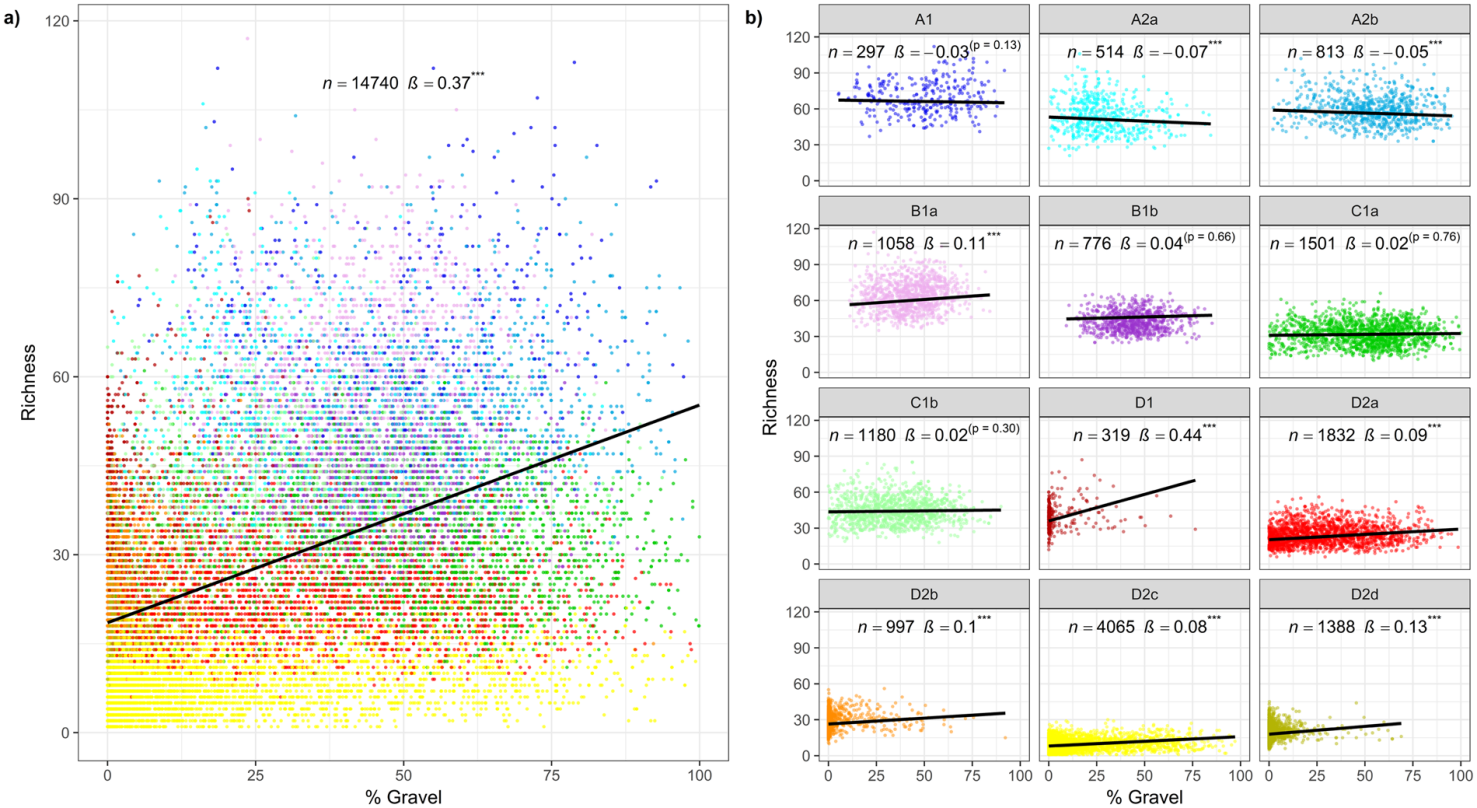
Scatter plots for taxon richness by percentage gravel for all samples (**a**) and by faunal cluster group (**b**). Black lines show the estimated slope for the relationship between richness and percentage gravel. Line intercepts relate to the median survey intercept for each plot. Plot annotations are provided for number of samples (*n*) and slope (*ß*). Slope values include statistical significance (***p < 0.001).

Using all available data, a plot of percentage gravel versus taxon richness shows a moderate positive relationship between the two variables (Fig. 10a). However, when the data are plotted by faunal cluster group the same relationship no longer applies (Fig. 10b), with the possible exception of faunal group D1. Whilst the slopes of some groups are significantly different from zero, this is a reflection of the large number of samples rather than a meaningful trend. This suggests the positive correlation seen in Fig. 10a results from the regression across cluster groups. Similar results were obtained for percentage gravel versus total abundance.

### Assessing the ecological significance of sediment change

The present study indicated that test sample ‘EEC2010_Site 102’ had a baseline faunal-physical cluster group identity of B1a_4. The sediment means and covariance matrix for this group are shown in Table 4. The test sample failed the Mahalanobis distance test (*p* < 0.05), and comparison of the test values with the means of the cluster group suggested the failure resulted principally from an excess of fine sand (fS, Table 4). Further testing of the approach at a former aggregate extraction site in the southern North Sea^13^ can be found in the Supplementary Note 1.

**Table 4:** Summary of a Mahalanobis distance test for the sediment composition of sample ‘EEC2010_Site 102’.

Test sample		S/C	fS	mS	cS	fG	mG	cG
Values (v)		1.9	17.8	36.3	23.7	13.4	3.9	3.1
Distribution data (B1a_4)		S/C	fS	mS	cS	fG	mG	cG
Means $(\bar{{\rm{x}}})$		1.0	4.3	24.2	26.8	22.8	12.4	8.6
Covariance Matrix	**S/C**	2.1						
	**fS**	0.9	6.4					
	**mS**	−1.4	9.2	58.5				
	**cS**	−2.7	−10.2	1.8	106.6			
	**fG**	−0.6	−4.1	−15.7	5.9	34.7		
	**mG**	1.2	0.3	−15.7	−43.4	6.6	41.2	
	**cG**	0.6	−2.6	−36.8	−58.0	−26.8	9.9	113.7
Difference		S/C	fS	mS	cS	fG	mG	cG
Difference $({\rm{v}}-\bar{{\rm{x}}})$		+0.9	+13.5	+12.1	−3.1	−9.4	−8.5	−5.5
% Difference $({\rm{v}}-\bar{{\rm{x}}}/\bar{{\rm{x}}})$∗100		+90	+314	+50	−12	−41	−69	−64

Table shows the sediment composition of the test sample, and the sediment means and covariance matrix for the wider distribution data (group B1a_4, *n* = 948). The *p*-value from the test was <0.05, indicating the test sample was unlikely to be associated with wider cluster group distribution. Sediment fractions responsible for the failure are identified by subtracting test values for each fraction from the wider cluster group means.

## Discussion

This study had 3 main objectives: (i) to produce a baseline assessment of the UK’s macrobenthic infauna, with a particular focus around sites and regions of marine aggregate dredging, (ii) to identify the range of sediment composition found in association with the different baseline faunal assemblages, and (iii) to develop a method for assessing the likely ecological significance of sediment change. Achieving these objectives is an important step in the development of the RSMP approach to monitoring^23,31^. This approach aims to improve sustainability by ensuring the sediment habitat, within the footprint of dredging effect, is able to support the return of the original faunal assemblage type after cessation of activities.

We identified 12 faunal assemblages and their distribution around the UK. Some of these assemblages were geographically isolated, whilst others were more widespread. With the exception of D2b, all assemblage types were represented, in at least one location, within areas of aggregate dredging interest. Spatial patterns in faunal assemblages were broadly consistent over time (by year and season), and were largely driven, as far as could be explained, by sediment composition and hydrodynamics. Notable differences in the composition of sediments were observed between samples from different faunal assemblage groups. Within these groups, some small differences in sediment composition were evident between samples from the different physical cluster regions (Fig. 9b), thus supporting the assessment of sediment change based on samples from the same faunal and physical cluster region.

The approach taken to assessing whether sediments remained within an acceptable condition (i.e. Mahalanobis distance test) was successful in identifying known problems at an existing monitoring site in the eastern English Channel dredging region^32^, and at a former aggregate extraction site in the southern North Sea^13^ (see Supplementary Information). Furthermore, in both examples a comparison of the sediment composition of the test samples with that of the wider cluster group(s) correctly identified the sediment fractions known to be responsible for the problem. A comparison of taxon richness by percentage gravel for individual faunal groups (Fig. 10) suggests that a reduction in the proportion of gravel would not necessarily lead to a decline in richness, so long as sediments remained within the limits defined by the Mahalanobis distance test.

Findings from this study are broadly consistent with those of other workers in terms of broadscale patterns in univariate faunal metrics^33^, the number and nature of faunal assemblages^34–36^, the consistent temporal patterns in faunal assemblages^34,37^, and the existence of the same faunal assemblage types on both the east and west sides of the UK^36^. In addition, other studies highlighted a similar set of environmental variables as influencing the distribution of faunal communities^31,37–40^, and identified a need to account for hydrodynamic factors where assessing animal-sediment relationships^38^. Finally, in contrast to earlier work^28,29^, we did not set out predefined sediment limits by faunal cluster group. We contend that the setting of sediment limits, as defined by the full sediment envelope, is statistically questionable given that individual sediment fractions are not independent. The approach taken in the present study also allows for the continual addition of new data, meaning decisions will always be based on the most comprehensive dataset available.

This study is important for a number of reasons. Firstly, it highlights the considerable quantity of benthic data which exists across government and industry. Through integration, standardisation and analysis of the combined dataset, this study has produced a faunal baseline, reducing the need for reliance on modelled data^41^. This work has been possible due to improvements in access to data, and a willingness among data owners to share information. Secondly, the study represents a significant step forward in our understanding of how sediment change is likely to affect benthic faunal assemblages. Whilst it has long been recognised that faunal assemblages differ in their sensitivity to changes in sediment composition^11^, and that a faunal recovery is often predicated on a physical recovery^13^, it has hitherto not been possible to quantify such relationships. Both the close relationship between sediments and the benthos, reported here and in the literature^38^, and the consistent temporal patterns in faunal assemblage distribution support the development of the RSMP approach.

The consistent spatio-temporal patterns in macrofauna are, perhaps, unsurprising given the key role of sediments in structuring faunal assemblages, and the fact that sediment distribution is moderated by hydrodynamic forces^37^. In addition, our analyses were undertaken at the family level, and this could obscure any changes at the lower taxonomic levels of genus and species. Within faunal cluster groups, the lack of a strong relationship between taxon richness/total abundance and percentage gravel requires some explanation. One possibility, at least within the mobile sandy areas, is that taxa are unable to colonise any gravel effectively due to the abrasive nature of sand in suspension.

It is important to acknowledge limitations associated with the present study. For instance, the acceptability of changes in sediment composition is fundamentally linked to the number and identity of faunal assemblages. In the present study there was no obvious clustering solution, and we justify our selection of 12 groups as a balance between capturing biological complexity, whilst ensuring an adequate number of replicates for analysis of sediment composition. With 12 groups we were able to capture approximately 70% of the inherent variability (Fig. 3a) and whilst the number of replicates was generally high, for some faunal-physical groups numbers were still low (see Supplementary Table S2). In future, as more data become available, the analyses could be repeated with a greater number of cluster groups. Whilst a more objective means of identifying cluster groups is available (i.e. SIMPROF)^42^, this approach is not suitable for use with k-means. Further, we contend that statistical significance is less important than deciding on an operationally good number for the purpose in question. The lack of an obvious faunal clustering solution supports the idea of there being a continuum rather than wholly distinct faunal assemblages. This is presumably the result of taxa which occur across multiple groups (e.g. *Spionidae*, *Nemertea*, *Capitellidae*). We also recognise that the faunal baseline presented in this study is based not on a one-off snapshot survey, but rather the accumulated picture from 48 years of macrobenthic surveys. Whilst this could be considered a weakness, the majority of samples (96%) were acquired post 2000, and the consistent spatio-temporal patterns identified in this study provide confidence in the results. Furthermore, by incorporating an element of temporal variation, the baseline is, arguably, more robust for detecting anthropogenic change against a background of natural variability. The analysis of data at family level might also be criticised. However, there is precedence for working at this level^43^, and we considered it to be the only pragmatic way to address the inevitable differences in taxonomic resolution between surveys and thus to create a standardised dataset. That this was necessary highlights the need for greater adoption of quality control measures for macrobenthic sample processing. There are also issues regarding the comparability of sediment data generated through a combination of sieving and laser sizing versus sieving only, and this requires further investigation.

This study provides a faunal baseline and methodology for assessing the ecological significance of sediment change. The approach will be now be used by the marine aggregates industry to assess the status of the seabed as part of their new Regional Seabed Monitoring Programme (RSMP)^23,31^. Where sediment samples fail the Mahalanobis distance test this should lead to further investigation to determine the most likely cause (e.g. natural variability, sampling issues, other human activities or aggregate dredging). Whilst it was developed for the UK marine aggregate industry, this approach could also be relevant for other offshore activities which can affect the composition of seabed sediments (e.g. dredge material disposal, offshore windfarms, pipelines, drilling for oil and gas). As recently highlighted^31^, the harmonisation and integration of offshore monitoring programmes could deliver significant benefits for industry and ﻿government. More widely, big data applications can help identify the likely significance of human-induced changes in the environment through an improved understanding of natural variability. This will lead to better management and improved sustainability.

It remains a hypothesis that faunal recovery will occur after dredging assuming that sediments are left within an acceptable condition, as assessed using a Mahalanobis distance test. As such, there is a need to test this hypothesis as and when opportunities arise. Additional work would also be useful to address the issue of comparability between sediment data generated through sieving and a combination of laser sizing and sieving (see above). Work is also required to understand the implications of the failure of sediment data to meet the assumption of multivariate normality. It is recommended that new data continue to be added to the existing dataset so that future analyses are always based on the best available evidence.

## Conclusion

A big data approach offers valuable insights into the natural variability inherent within different ecosystems. This understanding increases our ability to identify which human induced impacts are likely to have long-term ecological significance. This leads to more effective management, innovative and cheaper monitoring solutions, and ultimately, better environmental sustainability.

## Methods

### The dataset

**Figure 11:**
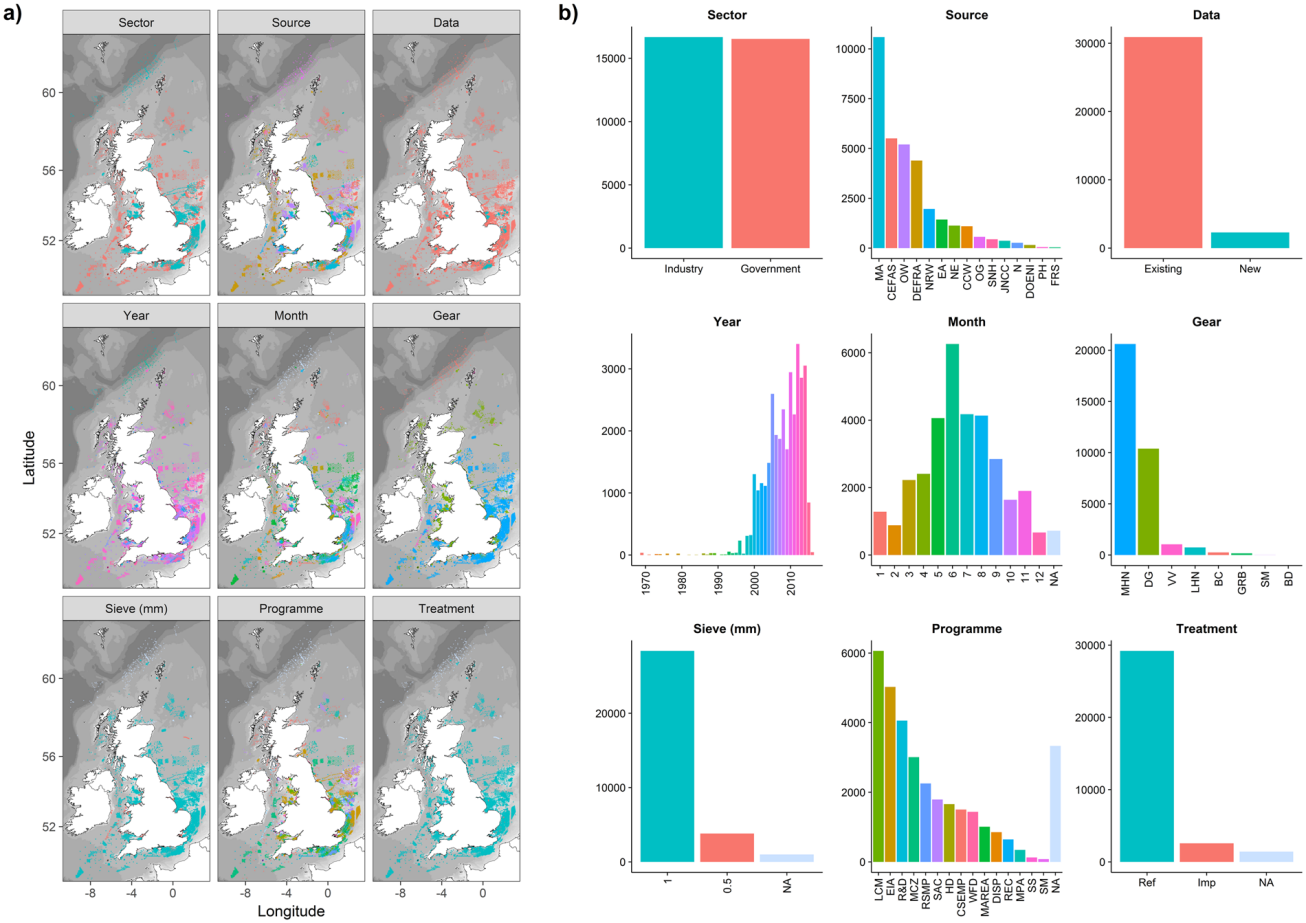
Dataset summary maps (**a**) and associated histograms (**b**) for Sector (industry or government data), Source (data owner), Data (newly acquired or existing data), Year (year of sample collection), Month (month of sample collection), Gear (device used to obtain sample), Sieve (mm) (size of sieve used for macrofaunal sample processing), Programme (reason for sample collection) and Treatment (sample relates to known ‘impact’ or ‘reference’ condition). See Supplementary Table S1 for list of abbreviations. Maps created in RStudio (version 1.0.143, https://www.rstudio.com/).

The dataset compiled for this study comprises of 33,198 macrofaunal samples (83% with associated data on sediment particle size composition) covering large parts of the UK continental shelf (Fig. 1b). Whilst the majority of samples come from existing datasets, also included are 2,500 new samples collected specifically for the purpose of this study (Fig. 11). These new samples were acquired during 2014–2016 from the main English aggregate dredging regions (Humber, Anglian, Thames, Eastern English Channel and South Coast) and at four individual, isolated extraction sites where the RSMP methodology is also being adopted (e.g. Area 457, North-West dredging region; Area 392, North-West dredging region; Area 376, Bristol Channel dredging region; Goodwin Sands, English Channel). This work was funded by the developers, and carried out by contractors on their behalf. Samples were collected in accordance with a detailed protocols document^44^ which included control measures to ensure the quality of faunal and sediment sample processing. Additional samples were acquired to fill in gaps in spatial coverage and to provide a contemporary baseline for sediment composition.

Sources of existing data include both government and industry, with contributions from the marine aggregate dredging, offshore wind, oil and gas, nuclear and port & harbour sectors. Samples have been collected over a period of 48 years from 1969 to 2016, although the vast majority (96%) were acquired since 2000. Samples have been collected during every month of the year, although there is a clear peak during summer months when weather conditions are generally more favourable for fieldwork. A variety of gear types have been used for sample collection including grabs (0.1 m^2^ Hamon, 0.2 m^2^ Hamon, 0.1 m^2^ Day, 0.1 m^2^ Van Veen and 0.1 m^2^ Smith McIntrye) and cores. Of these various devices, 93% of samples were acquired using either a 0.1 m^2^ Hamon grab or a 0.1 m^2^ Day grab. Sieve sizes used in sample processing include 1mm and 0.5mm, reflecting the conventional preference for 1mm offshore and 0.5mm inshore (see Fig. 11). Of the samples collected using either a 0.1 m^2^ Hamon grab or a 0.1 m^2^ Day grab, 88% were processed using a 1mm sieve.

Taxon names were standardised according to the WoRMS (World Register of Marine Species) list using the Taxon Match Tool (http://www.marinespecies.org/aphia.php?p=match). Of the initial 13,449 taxon names, only 4,248 remained after correction. The output from this tool also provides taxonomic aggregation information, allowing data to be analysed at different taxonomic levels - from species to phyla. Macrofaunal data were collated using the Primer 6® software package^45^, which allows for merging of individual datasets to produce a single taxon/sample matrix. Metadata pertaining to each sample were stored in a related ‘factors’ sheet within the Primer workspace. Due to the size of the dataset, it was split into multiple Primer workbooks to allow for ease of working. The final dataset comprises of a single sheet comma-separated values (.csv) file. Colonials accounted for less than 20% of the total number of taxa and, where present, were given a value of 1 in the dataset. This component of the fauna was missing from 325 out of the 777 surveys, reflecting either a true absence, or simply that colonial taxa were ignored by the analyst. Sediment particle size data were provided as percentage weight by sieve mesh size, with the dataset including 99 different sieve sizes. Sediment samples have been processed using sieve, and a combination of sieve and laser diffraction techniques. Key metadata fields include: Sample coordinates (Latitude & Longitude), Survey Name, Gear, Date, Grab Sample Volume (litres) and Water Depth (m). A number of additional explanatory variables (Table 5, variables 1–8) were acquired through interrogation of raster data layers using the *over* function in the statisical package R^46^. In total, the dataset dimensions are 33,198 rows (samples) × 13,588 columns (variables/factors), yielding a matrix of 451,094,424 individual data values. The dataset and associated files used in this study are available from the Cefas Data Hub (https://doi.org/10.14466/CefasDataHub.34), with the R script provided in the Supplementary Information.

**Table 5:** Explanatory variables used in the study.

Variable		Detail	Data Source
1	Sal	Mean annual near-bed salinity (ppt)	A
2	Temp	Mean annual near-bed temperature (°C)	A
3	Chl a	Mean annual chlorophyll-a concentration (2002–2010)	B
4	SPM	Mean annual concentration of mineral origin suspended matter (g·m^−3^) (2002–2010)	B
5	Depth	Bathymetry (m)	C
6	WOV	Peak wave orbital velocity (m·s^−1^)	D
7	AvCur	Average current velocity (m·s^−1^)	D
8	Stress	Peak wave/current stress (N·m^−2^)	D
9	Gravel	Proportion of gravel (%) from sediment particle size data	E
10	Sand	Proportion of sand (%) from sediment particle size data	E
11	Mud	Proportion of silt/clay (%) from sediment particle size data	E

Data sources: A (ICES climatology of surface and near-bed temperature and salinity 1971–2000)^56^; B (Data from MODIS satellite sensor)^57^; C (Defra DEM 500 m pixel resolution)^54^; D (POLCOM model)^58,59^; E (data from sediment particle size analysis).

In seeking to understand the relationship between fauna and sediments, it was important to identify and exclude any samples taken in previously impacted areas where faunal composition may not reflect a ‘natural’ state. For example, those sites which have been subject to aggregate dredging where the period of time between sample collection and last dredging is insufficient for recovery. To do this, sample locations were overlaid onto GIS layers (polygons) detailing the location of dredging in each year (available data from 1993 to 2015). An R script was used to calculate the number of years between sample collection and the time of last dredging. These values were then compared to the known faunal recovery times for different seabed landscapes^22^. For stations which had been subject to dredging, and where the time between last dredging and sampling was less than the predicted recovery time, the sample was flagged as ‘impacted’. Other samples known to have been taken within differently impacted areas (e.g. dredge material disposal sites) were similarly flagged. In this study we made no attempt to account for the effects of demersal fishing due to the widespread nature of the pressure, and a current lack of understanding of how it affects macroinfaunal assemblages.

### Faunal data analysis

#### Data subset

A subset of macrofaunal data (27,432 samples −83% of the dataset) was taken forward for analysis. This subset was based on comparable gears (all 0.1 m^2^ grabs), and where the macrofaunal sample had been sieved over a 1mm mesh. Whilst the different grab types will sample differently, it was considered that such differences were likely to be minor in relation to the spatial distribution patterns of interest. Aggregation data were used to output data at the family level, to take account of differences in taxonomic discrimination between surveys, and to reduce any noise associated with seasonal and inter-annual changes at the level of genus and species. Taxon records for fish, diatoms, parasites, zooplankton, seeds and eggs were excluded from the dataset. Samples with and without colonial taxa were included to achieve the best possible spatial coverage. Whilst this introduces a source of error into the dataset, the effect is limited by the relatively small contribution that colonial taxa make to the total number of families in the dataset.

#### Univariate indices

Univariate summary measures for taxon richness and total abundance were calculated for all samples using the vegan package^47^ in R. Plots were produced for each measure based on rank values, thereby showing the relative importance of different areas.

#### Community analysis

Data were subjected to a fourth-root transformation to ensure the appropriate weighting of colonial and rarer taxa^48^ in the analyses. Clustering was undertaken using the k-means (R function *kmeans*) approach with the MacQueen algorithm^49^. This clustering method works by choosing the cluster solution that minimises the within cluster sum of squares, summed over all variables and clusters. A k-means clustering approach was specifically chosen due to (i) its utility for analysing large datasets (hierarchical clustering approaches would not work with a dataset of this size), and (ii) the ability to, in future, match new sample data to existing cluster groups. This is vital for the method under development (to allow new extraction sites to adopt the approach), and is similarly not possible using hierarchical clustering approaches. The number of cluster groups was decided through reference to an ‘elbow plot’^50^ and represents a balance between representing biological complexity, whilst also ensuring adequate replication for confidently assessing faunal-sediment relationships (see below).

To establish the relationship (i.e. similarity/dissimilarity) between the different faunal cluster groups, we computed the absolute distances between each of the cluster centres across all variables (R function *dist*). The resulting dissimilarity matrix was then used to generate a dendrogram based on group average hierarchical clustering (R function *hclust*). Informed by the dendrogram, each group was assigned a code (and colour) to show the relatedness of the groups.

The SIMPER routine in Primer 6® was used to identify characterising taxa associated with each faunal cluster group. Mean and standard deviations were calculated for the univariate measures of taxon richness and total abundance by faunal cluster group. An indication of the relative size of values (high, medium or low) was determined by division of the full range of mean values across all cluster groups into 3 tertiles.

#### Temporal assessment

A subjective assessment of the temporal stability of spatial patterns in faunal assemblages was made by plotting samples by year group and season.

#### Explaining patterns in faunal distribution

The extent to which variation in the macrofaunal data could be explained by sediment composition and other environmental variables (see Table 5) was explored using the *best* and *adonis* functions from the vegan^47^ package in R. The *best* function identifies the subset of environmental variables with maximum (rank) correlation with community dissimilarities (i.e. those that ‘best explain’ the faunal data). The *adonis* function performs a permutational multivariate analysis of variance using distance matrices, to determine how much of the variability in the macrofaunal data can be explained by the predictor variables. A distance based redundancy analysis (dbRDA) ordination plot was used to visualise the relationship between macrofaunal data (samples) and predictor variables.

Analyses were based on a 3,564-sample subset of the data, comprising 297 randomly chosen samples from each of the 12 faunal cluster groups. We chose the same number of available samples across all the groups to avoid any bias in the results. Macrofaunal data were fourth-root transformed, to down-weight the influence of highly abundant taxa, and resemblances calculated using the Bray-Curtis measure. Corresponding environmental variables (see Table 5) for each sample were initially assessed for collinearity through examination of Variation Inflation Factors (VIFs)^51^. VIFs were calculated for each variable using the function *vif* from the usdm package in R. The variable with the highest VIF score above 2.5 was deleted before the VIF scores for remaining variables was recalculated. This process was repeated until all remaining variables had VIF scores of <2.5. This process led to the exclusion of Temp, Gravel, Chl a and Stress. These variables were highly correlated with Latitude (Spearman Rank correlation (ρ) = −0.8), Sand (ρ = −0.8), SPM (ρ = 0.9) and AvCur (ρ = 0.8) respectively. Log(x + 0.1) transformations were applied to SPM, Depth and Mud to address right skewness, before all selected environmental variables were normalised to bring them on to a common scale. Resemblances for the environmental data were calculated using Euclidean distance. To gain additional insight into faunal distribution patterns, heat maps were produced for each explanatory variable, with stations coloured according to their ranked value.

#### Faunal distribution within areas of aggregate industry interest

To fully satisfy the study’s first objective (to create a baseline assessment of benthic macrofauna, with a particular focus around sites and regions of marine aggregate dredging), a series of more detailed maps were produced for faunal cluster identity and taxon richness across regions of aggregate dredging interest. Due to the high correlation between taxon richness and total abundance, and for the sake of brevity, maps of total abundance are not presented. All stations within the footprint of potential dredging effect, and a subset of stations within the wider region, will be monitored under the RSMP approach^31^ to assess for changes in sediment composition.

### Faunal-sediment relationships

This section addresses the study’s second objective: to assess the composition of sediments found in association with different faunal cluster groups. It is recognised that the precise range of sediment composition found in association with any particular faunal group may vary in different parts of UK waters due to the influence of other variables (see section ‘Explaining Patterns in faunal distribution’). To control for this, and to ensure that any assessment of sediment change (objective 3) is based only on relevant data, we assessed the composition of sediments by a combination of faunal and faunal-physical cluster group. Physical cluster groups, established through a k-means clustering of the non-sediment variables shown in Table 5, were intended to show samples which were subject to similar environmental conditions. In this section, we also assess the likely implications of a reduction in the proportion of gravel for taxon richness and total abundance.

#### Data subset

A slightly reduced dataset (13,392 samples, 49% of samples selected for faunal analysis) was available for sediment analysis as only samples collected using a 0.1 m^2^ Hamon grab were selected; this is the device routinely used by the aggregates industry for monitoring. In addition, samples were excluded where: (i) there was no sediment data, (ii) the sediment data came from a different grab deployment to that of the fauna, (iii) the total sediment percentage was not 100% ± 1, (iv) the top sieve was not empty (allowing for the possibility that some material could have been retained on a larger sieve had it been used), (v) the sample had been taken from a known impacted site, and (vi) the associated faunal data did not include colonial taxa. The sediment data were available as percentage weights across 99 sieve classes. These classes were initially collapsed into a comparable set of 12 standard sieve sizes, before data were further summarised according to the sediment classes defined by Wentworth^52^ (Table 6).

**Table 6:** Standard sieve classes and sediment descriptions based on the Wentworth scale^52^.

Sieve (mm)	Wentworth Size Class	
64	Cobbles	**GRAVEL**
32	Coarse gravel (cG)	
16		
8	Medium gravel (mG)	
4	Fine gravel (fG)	
2		
1	Coarse sand (cS)	**SAND**
0.5		
0.25	Medium sand (mS)	
0.125	Fine sand (fS)	
0.063		
Pan	Silt/Clay (S/C)	**MUD**

#### Identification of physical cluster groups

Prior to the clustering, log(x + 0.1) transformations were applied to individual environmental variables (SPM, Depth and Stress) to address right skewness, before normalisation to bring them on to a common scale. As before, the number of cluster groups was decided through reference to an ‘elbow plot’. A dendrogram, based on the between group distances, was produced to reveal the relationship (similarity/dissimilarity) between the different physical cluster groups. This information will be useful where the number of replicates with which to assess sediment composition (see section 2.4) is low, allowing for additional samples from a closely related cluster group to be included in the analysis. A box and whisker plot was produced to show how variable values differ across physical cluster groups.

#### Sediment composition by faunal and faunal-physical cluster groups

Cumulative distribution plots were produced to show the mean sediment composition across the standard sieve sizes (see Table 6) for each faunal cluster group and each faunal-physical cluster group. The faunal cluster group plots included a histogram showing the proportion of sediment across each sieve size. The mean sediment composition by Wentworth size class was also tabulated. The Index of Multivariate Dispersion (MVDISP)^45^ was used to indicate the variability in sediment composition within cluster groups; higher values of MVDISP indicate higher variability.

#### Relationship between gravel and taxon richness/total abundance

As gravel rich sediments are generally associated with higher biodiversity^33^, there is a need to assess whether a reduction in gravel content could potentially lead to a decline in taxon richness and total abundance of the benthos. To investigate this, taxon richness and total abundance (response variables) were plotted against percentage gravel (explanatory variable), first using all the data and then by faunal cluster group. We initially fitted a standard linear regression model to these data. We recognised that such models are only approximations to the true data as richness is a discrete variable and because it cannot go below zero. However, because the richness values were generally high (mean overall of 30) we considered that the linear regression assumptions of Normally distributed errors with constant variance would be well approximated. Analysis of the residuals (Q-Q plots and residuals plotted against fitted values) supported our prior views. However, the residuals did exhibit autocovariance of observations with preceding observations. This suggests a lack of independence between observations.

An obvious potential reason for this dependence is that our dataset is an amalgam of data from hundreds of different surveys. Clearly, richness values from the same survey are likely to be more similar than those from different surveys. We thus fitted Survey as a factor in our linear regression models. Effectively, we are then fitting a different intercept value for each survey, but with the slope remaining constant. Analysis of the residuals from these “Gravel + Survey” models showed that the autocorrelations had indeed been taken account of by the Survey variable.

### Assessing the ecological significance of sediment change

This section addresses the final study objective: to develop a method for assessing the likely ecological significance of sediment change. Under the new monitoring regime being adopted by the aggregates industry (i.e. the RSMP), the composition of sediments at individual monitoring stations will be routinely checked. This will ensure that conditions remain ‘favourable’ for the return of the original faunal assemblage type after cessation of dredging. Here we define favourable as meaning the sediment composition of the monitoring sample is, in a statistical sense, likely to be associated with the wider sediment distribution for the relevant cluster group (faunal or faunal-physical). This assessment was made using a Mahalanobis distance test^53^, which assesses departure of the test sample from the cluster group distribution, assuming multivariate normality. The test, often used to identify outliers in a dataset, is equivalent in one dimension to identifying values which are greater than 1.96 standard deviations from the mean. Input data are required from the monitoring sample (percentages of silt/clay, fine sand, medium sand, coarse sand, fine gravel, medium gravel and coarse gravel) and the wider distribution (mean values for each sediment fraction and a covariance matrix). The test returns a value for *p* which is the probability that the test sample belongs to the wider set. Where a sample has failed (i.e. *p* < 0.05), the reason for the failure (i.e. which sediment fraction(s) is/are responsible) was determined by comparing the test sample values with those of the wider distribution means. We demonstrate this method using a sample (EEC2010_Site 102) from the aggregate industry’s eastern English Channel monitoring program. This sample was acquired during the 2010 monitoring survey from site 102, located within extraction area 473/2. Earlier studies had suggested the sediments at this site had been modified by dredging activity^32^ and that, were the changes to persist, they were likely to have implications for faunal recovery^29^. Further testing of the Mahalanobis approach, using data from a seabed recovery study^13^, is provided with the Supplementary Information accompanying this paper.

## Electronic supplementary material

Supplementary Information
