# Supplementary material for: A big data approach to macrofaunal baseline assessment, monitoring and sustainable exploitation of the seabed
Source: Sci Rep. 2017 Sep 29;7:12431. doi: 10.1038/s41598-017-11377-9 (PMC5622093; doi:10.1038/s41598-017-11377-9)
Supplement: Supplementary file 1 — Supplementary Information [file 41598_2017_11377_MOESM1_ESM.pdf]

SUPPLEMENTARY INFORMATION

A big data approach to macrofaunal baseline assessment, monitoring and sustainable exploitation of the seabed

Cooper, K.M. and Barry, J.

Supplementary Table S1. Abbreviations used in Figure 11.

Factor	Abbreviation	Detail
Source	MA	Marine Aggregates
	CEFAS	Centre for Environment, Fisheries and Aquaculture Science
	OW	Offshore Wind
	DEFRA	Department for Environment, Food and Rural Affairs
	NRW	Natural Resources Wales
	EA	Environment Agency
	NE	Natural England
	CCW	Countryside Council for Wales
	OG	Oil and Gas
	SNH	Scottish Natural Heritage
	JNCC	Joint Nature Conservation Committee
	N	Nuclear
	DOENI	Department of Environment Northern Ireland
	PH	Ports & Harbours
	FRS	Fisheries Research Services
Gear	MHN	0.1m^2 Hamon grab
	DG	0.1m^2 Day grab
	VV	0.1m^2 Van Veen grab
	LHN	0.2m^2 Hamon grab
	BC	Box Core, unknown dimensions
	GRB	unspecified grab
	SM	0.1m^2 Smith McIntyre grab
	BD	Box Dredge
Programme	LCM	Licence Compliance Monitoring
	EIA	Environmental Impact Assessment
	R & D	Research and development
	MCZ	Marine Conservation Zone
	RSMP	Regional Seabed Monitoring Programme
	SAC	Special Area of Conservation
	HD	Habitats Directive
	CSEMP	Clean Seas Environmental Monitoring Programme
	WFD	Water Framework Directive
	MAREA	Marine Aggregate Regional Environmental Assessment
	DISP	Dredge Material Disposal
	REC	Regional Environmental Characterisation
	MPA	Marine Protected Area
	SS	Sewage Sludge Disposal
	SM	Statutory Monitoring
	NA	unknown

Supplementary Table S2. Mean percentage composition of sediments by Wentworth size class for each faunal-physical cluster group. Sediment descriptions are in accordance with Blott and Pye (2011)[55]. Number of samples is shown by column *n*. Variability in sediment composition is indicated by the Multivariate Index of Dispersion (MVDISP).

Cluster			% Mud	% Sand				% Gravel					
Bio	Phy	*n*	Sum	fS	mS	cS	Sum	fG	mG	cG	Sum	Description	MVDISP
A1	1	2	9	9	5	21	35	18	10	27	55	slightly muddy sandy gravel	1.23
	3	7	5	10	15	14	39	14	11	31	55	slightly muddy sandy gravel	1.11
	4	4	5	21	10	17	48	22	7	18	47	slightly muddy gravelly sand	1.13
	6	10	4	10	13	9	32	13	14	37	64	v slightly muddy sandy gravel	0.87
	7	260	4	17	17	13	47	18	12	20	50	v slightly muddy sandy gravel	0.95
	8	2	3	12	35	5	52	9	13	22	44	v slightly muddy gravelly sand	0.00
	9	2	41	15	19	5	39	6	6	8	20	gravelly sandy mud	1.93
A2a	2	14	12	15	22	17	55	19	10	5	33	slightly muddy gravelly sand	0.39
	3	23	6	27	22	14	63	16	7	8	31	slightly muddy gravelly sand	0.84
	4	1	1	1	5	12	17	6	7	68	81	v slightly muddy slightly sandy gravel	NA
	5	25	13	27	12	12	52	15	11	10	36	slightly muddy gravelly sand	0.86
	6	11	7	17	24	21	62	12	8	11	31	slightly muddy gravelly sand	1.16
	7	262	7	33	20	12	64	15	7	7	28	slightly muddy gravelly sand	0.88
	8	107	8	26	25	15	66	10	7	8	26	slightly muddy gravelly sand	1.03
	9	9	14	11	24	28	63	12	5	6	23	slightly muddy gravelly sand	0.64
	10	1	2	5	4	17	26	32	22	18	72	v slightly muddy sandy gravel	NA
A2b	1	11	14	11	20	28	59	14	6	7	26	slightly muddy gravelly sand	0.66
	3	22	7	9	21	29	59	18	8	8	34	slightly muddy gravelly sand	0.80
	4	102	3	8	13	16	37	15	11	34	59	v slightly muddy sandy gravel	1.01
	5	8	5	19	9	12	40	16	14	25	55	v slightly muddy sandy gravel	0.34
	6	362	4	13	13	11	37	16	14	29	58	v slightly muddy sandy gravel	0.96
	7	28	5	12	12	12	35	19	12	29	60	v slightly muddy sandy gravel	0.98
	8	129	15	8	15	16	39	12	13	21	46	slightly muddy sandy gravel	0.97
	9	57	12	8	20	30	58	12	7	11	30	slightly muddy gravelly sand	0.78
	10	8	10	10	11	39	60	27	3	0	30	slightly muddy gravelly sand	0.81
B1a	1	18	7	10	13	43	66	17	5	5	27	slightly muddy gravelly sand	0.84
	3	29	4	8	26	36	70	17	5	5	27	v slightly muddy gravelly sand	0.56
	4	948	1	4	24	27	55	23	12	9	44	v slightly muddy gravelly sand	0.61
	7	1	2	7	12	28	47	25	13	14	52	v slightly muddy sandy gravel	NA
	8	5	12	12	32	14	57	14	5	12	30	slightly muddy gravelly sand	1.20

Supplementary Table S2. Mean percentage composition of sediments by Wentworth size class for each faunal-physical cluster group. Sediment descriptions are in accordance with Blott and Pye (2011)[55]. Number of samples is shown by column *n*. Variability in sediment composition is indicated by the Multivariate Index of Dispersion (MVDISP).

Cluster		*n*	% Mud	% Sand				% Gravel				Description	MVDISP
Bio	Phy		Sum	fS	mS	cS	Sum	fG	mG	cG	Sum		
	9	1	18	4	9	35	48	25	10	0	35	slightly muddy gravelly sand	NA
	10	6	5	2	4	38	44	34	10	7	51	v slightly muddy sandy gravel	0.92
B1b	1	4	5	9	18	36	64	18	7	6	31	slightly muddy gravelly sand	0.54
	3	12	1	4	11	28	43	31	13	12	56	v slightly muddy sandy gravel	0.72
	4	756	2	5	24	25	54	22	13	9	44	v slightly muddy gravelly sand	0.62
	8	1	7	4	9	35	47	17	17	12	45	slightly muddy gravelly sand	NA
	9	1	8	1	9	35	46	29	12	4	46	slightly muddy sandy gravel	NA
C1a	1	22	5	13	20	31	65	12	7	11	30	slightly muddy gravelly sand	0.79
	2	12	15	20	23	14	57	13	7	7	27	slightly muddy gravelly sand	1.05
	3	43	4	12	24	20	57	22	8	10	40	v slightly muddy gravelly sand	0.87
	4	181	2	5	17	22	44	18	13	22	53	v slightly muddy sandy gravel	1.01
	5	164	5	19	11	14	44	18	16	18	51	v slightly muddy sandy gravel	0.78
	6	329	3	10	14	14	37	17	15	28	60	v slightly muddy sandy gravel	1.02
	7	210	3	18	18	14	49	18	11	18	47	v slightly muddy gravelly sand	1.21
	8	301	10	11	24	16	51	13	12	15	39	slightly muddy gravelly sand	1.20
	9	49	7	8	24	32	64	14	6	9	29	slightly muddy gravelly sand	0.89
	10	4	2	4	5	29	39	28	16	15	59	v slightly muddy sandy gravel	0.87
C1b	1	74	9	18	20	22	61	14	6	10	31	slightly muddy gravelly sand	0.99
	2	6	3	18	30	9	58	21	9	9	39	v slightly muddy gravelly sand	0.86
	3	199	7	21	22	17	60	20	7	6	33	slightly muddy gravelly sand	0.79
	4	61	6	13	31	18	63	16	8	7	31	slightly muddy gravelly sand	0.94
	5	29	6	26	10	12	48	16	12	18	45	slightly muddy gravelly sand	0.85
	6	52	5	20	16	13	48	17	12	17	46	slightly muddy gravelly sand	1.00
	7	46	5	26	21	12	59	21	9	7	36	v slightly muddy gravelly sand	1.02
	8	483	13	13	21	15	49	13	13	12	39	slightly muddy gravelly sand	1.06
	9	54	7	18	30	26	74	13	4	2	19	slightly gravelly slightly muddy sand	0.79
	10	4	7	13	18	40	71	18	4	1	23	slightly muddy gravelly sand	0.59
D1	1	22	11	63	17	6	85	2	1	2	4	v slightly gravelly slightly muddy sand	0.82
	3	37	17	55	13	8	76	5	2	0	7	slightly gravelly slightly muddy sand	1.20
	4	1	2	39	23	11	72	11	7	7	26	v slightly muddy gravelly sand	NA

Supplementary Table S2. Mean percentage composition of sediments by Wentworth size class for each faunal-physical cluster group. Sediment descriptions are in accordance with Blott and Pye (2011)[55]. Number of samples is shown by column *n*. Variability in sediment composition is indicated by the Multivariate Index of Dispersion (MVDISP).

Cluster			% Mud	% Sand				% Gravel					
Bio	Phy	*n*	Sum	fS	mS	cS	Sum	fG	mG	cG	Sum	Description	MVDISP
	5	4	16	28	16	17	61	12	5	6	23	slightly muddy gravelly sand	0.83
	6	1	0	24	6	7	36	15	20	30	64	sandy gravel	NA
	7	13	15	22	29	15	66	15	3	1	19	slightly gravelly slightly muddy sand	1.06
	8	30	21	29	29	8	66	7	4	3	13	slightly gravelly muddy sand	1.26
	9	29	12	41	31	11	83	4	1	0	6	slightly gravelly slightly muddy sand	0.96
	10	5	20	48	8	8	64	12	3	1	16	slightly gravelly slightly muddy sand	1.37
D2a	1	275	7	16	27	34	76	11	3	3	17	slightly gravelly slightly muddy sand	1.12
	2	3	0	8	39	34	81	16	1	3	19	slightly gravelly sand	0.41
	3	328	2	10	21	32	63	19	8	8	35	v slightly muddy gravelly sand	1.15
	4	362	1	5	31	33	70	18	6	4	29	v slightly muddy gravelly sand	0.91
	5	26	3	17	14	23	54	16	14	13	43	v slightly muddy gravelly sand	1.02
	6	41	2	9	17	18	45	22	17	15	54	v slightly muddy sandy gravel	1.15
	7	114	2	14	24	27	64	21	7	6	34	v slightly muddy gravelly sand	1.22
	8	281	5	11	32	25	68	13	8	7	27	v slightly muddy gravelly sand	1.22
	9	42	4	7	35	37	79	11	4	2	17	v slightly muddy slightly gravelly sand	1.05
	10	42	2	5	17	51	73	19	3	3	25	v slightly muddy gravelly sand	1.25
D2b	1	476	15	49	21	9	80	3	1	2	5	slightly gravelly slightly muddy sand	1.24
	2	4	10	35	16	15	66	14	6	3	23	slightly muddy gravelly sand	0.69
	3	73	22	41	19	12	71	5	1	1	7	slightly gravelly muddy sand	1.22
	4	2	27	16	50	4	70	2	1	0	3	v slightly gravelly muddy sand	1.74
	5	3	15	23	12	18	53	11	10	10	32	slightly muddy gravelly sand	1.27
	6	10	9	25	19	13	57	14	8	12	34	slightly muddy gravelly sand	1.29
	7	3	9	46	15	7	68	10	5	9	23	slightly muddy gravelly sand	1.31
	8	9	21	28	14	13	55	11	9	4	24	gravelly muddy sand	1.34
	9	10	10	40	35	11	87	1	0	2	3	v slightly gravelly slightly muddy sand	1.29
	10	6	35	56	6	1	64	0	0	0	1	muddy sand	1.43
D2c	1	194	9	32	39	16	88	2	1	1	3	v slightly gravelly slightly muddy sand	1.13
	2	94	14	39	32	6	76	4	2	4	10	slightly gravelly slightly muddy sand	1.53
	3	291	3	24	43	19	86	5	3	3	11	v slightly muddy slightly gravelly sand	1.25
	4	305	1	9	57	21	87	7	3	2	12	slightly gravelly sand	1.12

Supplementary Table S2. Mean percentage composition of sediments by Wentworth size class for each faunal-physical cluster group. Sediment descriptions are in accordance with Blott and Pye (2011)[55]. Number of samples is shown by column *n*. Variability in sediment composition is indicated by the Multivariate Index of Dispersion (MVDISP).

Cluster			% Mud	% Sand				% Gravel					
**Bio**	**Phy**	***n***	**Sum**	**fS**	**mS**	**cS**	**Sum**	**fG**	**mG**	**cG**	**Sum**	**Description**	**MVDISP**
	5	202	9	46	22	12	80	5	3	4	11	slightly gravelly slightly muddy sand	1.52
	6	300	3	25	33	19	77	8	5	7	20	v slightly muddy gravelly sand	1.52
	7	820	1	21	47	18	86	6	3	4	13	v slightly muddy slightly gravelly sand	1.25
	8	1098	3	16	49	19	84	6	4	3	12	v slightly muddy slightly gravelly sand	1.21
	9	73	9	8	48	28	84	4	2	1	7	slightly gravelly slightly muddy sand	1.21
	10	82	1	33	34	29	96	2	0	0	3	v slightly gravelly sand	1.20
D2d	1	202	3	69	19	5	93	2	1	1	4	v slightly gravelly v slightly muddy sand	0.57
	2	12	1	30	42	9	80	8	5	5	19	v slightly muddy slightly gravelly sand	1.25
	3	607	3	63	24	6	93	2	1	0	4	v slightly gravelly v slightly muddy sand	0.88
	4	32	2	26	64	7	96	1	0	0	2	v slightly gravelly v slightly muddy sand	0.68
	5	12	7	68	16	4	88	2	1	1	5	v slightly gravelly slightly muddy sand	1.34
	7	11	2	61	29	5	95	2	0	1	3	v slightly gravelly v slightly muddy sand	1.19
	8	77	4	30	55	4	89	3	2	2	7	v slightly muddy slightly gravelly sand	1.19
	9	18	2	28	53	16	97	1	0	0	1	v slightly gravelly v slightly muddy sand	1.00
	10	36	5	68	23	3	94	1	0	0	1	v slightly muddy sand	1.18

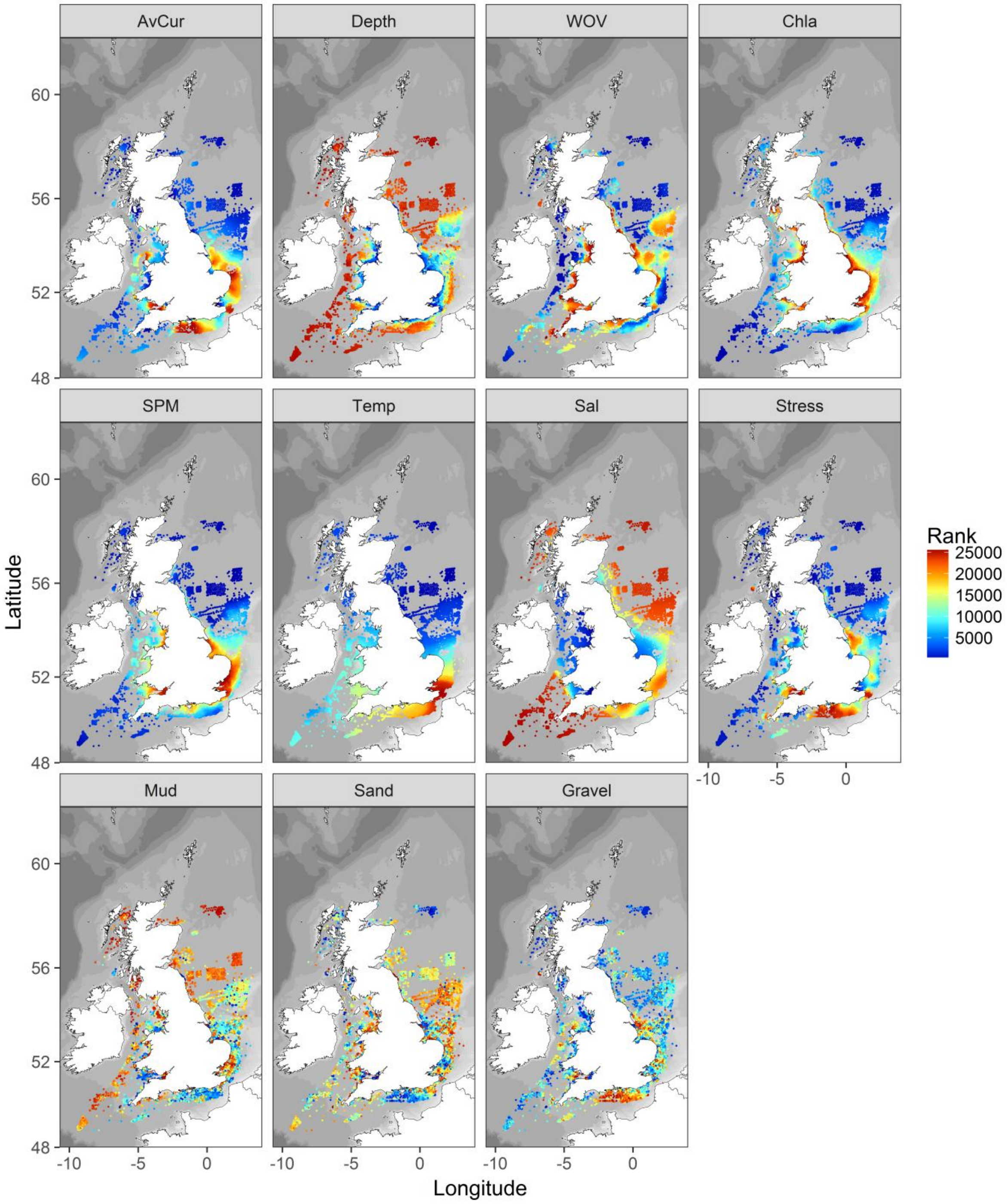

Supplementary Figure S1. Heatmaps indicating the relative values for each environmental varaible across all sample locations. Maps created in RStudio (version 1.0.143, https://www.rstudio.com/).

Supplementary Note 1: Further testing of Mahalanobis

Data from a seabed recovery study[13] offers a further opportunity for testing of the Mahalanobis distance test approach. In this study, ten grab samples (analysed for macrofauna and sediment particle size) were collected from both a dredged and reference condition at 5, 6, 7, 8, 11 and 15 years after cessation of aggregate dredging. Results showed initially elevated levels of coarse sand within the dredged area. This material gradually dissipated, although proportions of coarse sand did not return to background (reference) levels until 11 years after dredging. This persistent physical impact was thought to be responsible for the prolonged faunal recovery, which was not observed until year 15.

Based on these findings, it would be expected that many of the samples from the dredged site would fail a Mahalanobis distance test in years 5-8, with coarse sand identified as the most likely cause. Whilst the baseline faunal identity of samples from this study was not known, the reference site suggests the most likely assemblages were groups A2b_8, C1a_8 and C1b_8. As such, the composition of sediments from both dredged and reference sites was assessed, using Mahalanobis, to the sediment distribution of these combined groups. With the exception of one sample in 2011, all samples from the reference site passed the Mahalanobis distance test in all years of the study. In contrast, and, as predicated, the majority of samples from the dredged site failed the Mahalanobis distance test in year 5 (Figure S2). The number of failing samples at this site decreased over time, with all samples passing the test in years 11 and 15. In all cases of failure, coarse sand was identified as the most likely cause. These results suggest that sediment composition is a good predictor of macrofaunal recovery.

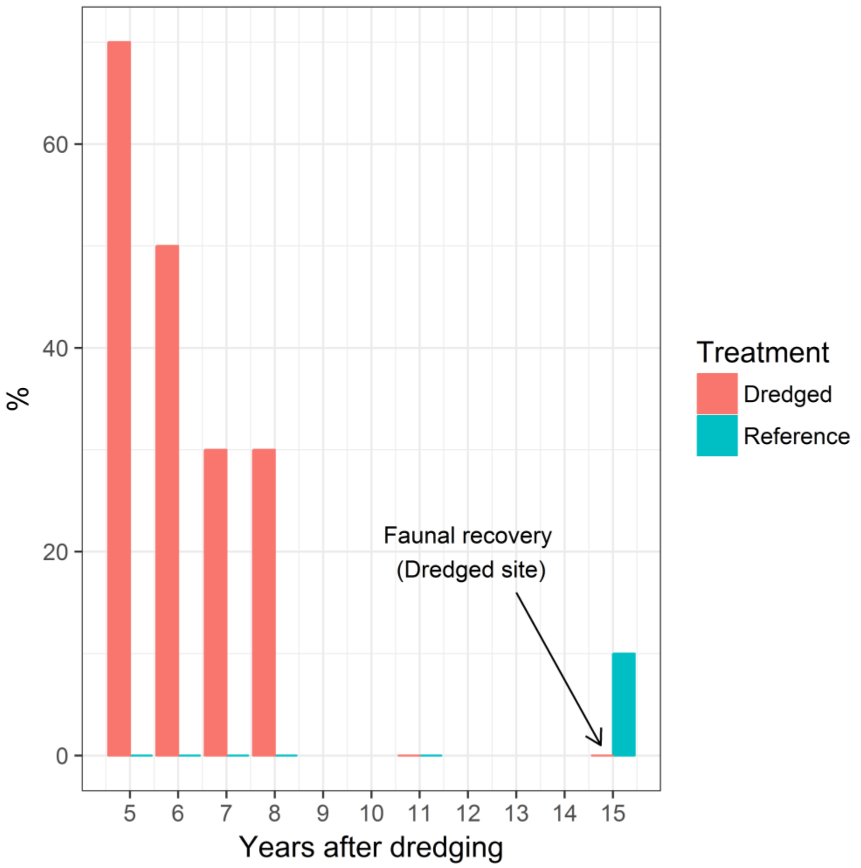

Supplementary Figure S2. Proportion of samples from dredged and reference sites which failed a Mahalanobis distance test over the course of a seabed recovery study undertaken at aggregate extraction Area 222 (southern North Sea)[13].

R Script

```
###############################################################################################
###############################################################################################
##                                                                                           ##
##                 R SCRIPT FOR ANALYSIS OF DATA IN COOPER & BARRY (2017)                    ##
##                                                                                           ##
###############################################################################################
###############################################################################################

## Reference:       Cooper, K.M., Barry, J. A big data approach to macrofaunal baseline
#                   assessment, monitoring and sustainable exploitation of the seabed. Scientific
#                   Reports (2017).

## Required Files:
#                   1.  C5922 FINAL SCRIPTV91.R (i.e. this script)
#                   2.  C5922DATASET13022017.csv (Raw data)
#                   3.  Dataset description.xlsx (Description of data in C5922DATASET13022017.csv)
#                   4.  PARTBAGG18112016.csv  (Faunal Aggregation data)
#                   5.  EUROPE.shp (European Coastline)
#                   6.  EuropeLiteScoWal.shp (European Coastline with UK boundaries)
#                   7.  Aggregates_Licence_20151112.shp (Aggregates Licensed extraction areas)
#                   8.  Aggregates_Application_20150813.shp (Aggregates Application areas)
#                   9.  HUMBERLICANDAPP.shp (Licensed Extraction and Application Areas - Humber)
#                   10. H_SIZ_PSD_POLYGONS_UNION_2014.shp (Humber SIZs)
#                   11. H_492_PIZ_APP.shp (Area 492 Application Area)
#                   12. ANGLIANLICANDAPP.shp (Licensed Extraction and Application Areas - Anglian)
#                   13. A_SIZ_PSD_POLYGONS_UNION.shp (Anglian SIZs)
#                   14. THAMESLICANDAPP.shp (Licensed Extraction and Application Areas - Thames)
#                   15. T_SIZ_PSD_POLYGONS_UNION_REV_2014.shp (Thames SIZs)
#                   16. T_501_1_2_SIZ_PSD.shp (Area 501 1/2 SIZ)
#                   17. EECLICANDAPP.shp (Licensed Extraction and Application Areas-East Channel)
#                   18. EC_SIZ_PSD_POLYGONS_UNION_REV.shp (East Channel SIZs)
#                   19. SCOASTLICANDAPP.shp (Licensed Extraction and Application Areas - South
#                       Coast)
#                   20. SC_SIZ_PSD_POLYGONS_UNION.shp (South Coast SIZs)
#                   21. BRISTOLCHANNELLICANDAPP.shp (Licensed Extraction and Application Areas -
#                       Bristol Channel)
#                   22. BC_SIZ2.shp (Bristol Channel/Severn Estuary SIZs)
#                   23. NORTHWESTLICANDAPP.shp(Licensed Extraction and Application Areas -
#                       North West)
#                   24. NW_392_SIZ_PSD_LICENCE_EXISTING.shp (Area 392 SIZ)
#                   25. AREA_457_PSD.shp (Area 457 SIZ)
#                   26. GOODWIN LICENCE FINAL POLYGON.shp (Goodwin Sands Extraction area)
#                   27. GoodwinSIZ.shp (Goodwin Sands SIZ)
#                   28. DEFRADEMKC8.shp (Seabed bathymetry)

## Required folder structure:
#                   C:\C5922 FINAL\R
#                                 \DATA
#                                 \OUTPUTS

## Folder Contents:
#                   C:\C5922 FINAL\R (file 1)
#                   C:\C5922 FINAL\DATA (files 2-28)
#                   C:\C5922 FINAL\OUTPUTS (.png and .csv files resulting from script)

## Set working directory
setwd('C://C5922 FINAL')

## Script Structure:
#                   The script will generate the figures and content of tables in Cooper & Barry
#                   (2017). The script is divided into 8 sections (see below) and 68 individual
#                   steps.  Each numbered step has a header box with a reference to the approriate
#                   section in the paper, a task description and a notes section. Note that steps
#                   33 and 54 are undertaken using the Primer 6 software.  The first section of the
#                   script deals with the creation of a dataset at the family level (Steps 1-6).
#                   This only needs to be done once, as the resulting file can be loaded during
#                   subsequent use of the script (i.e. start at step 7).

## Script Sections:
#                   A)  BUILD DATASET                  (Step 1:6)
#                   B)  LOAD DATASET                   (Step 7)
```

```
#                   C)  MAPPING LAYERS                (Step 8)
#                   D)  DATASET SUMMARY               (Step 9:15)
#                   E)  FAUNAL ANALYSIS               (Step 16:41)
#                   F)  SEDIMENT ANALYSIS             (Step 42:59)
#                   G)  GRAVEL/RICHNESS CHECK         (Step 60:65)
#                   H)  ASSESSING SEDIMENT CHANGE     (Step 66:68)

###############################################################################################
# # #                                  (A) BUILD DATASET                                  # # #
###############################################################################################

# The underlying dataset (C5922DATASET13022017.csv) used in this script is a station by variable
# matrix, with faunal data at the level of species and above. As the data will be analysed at the
# family level it is first necessary to create a sample by variable matrix with faunal data at
# the # family level. To do this the raw data are imported and converted to family level using an
# aggregatrion file. The large size of the raw data matrix means that it cannot be easily
# manipulated in R. Therefore, following loading, the data is split into 2 chunks.  Each chunk is
# seperatley manipulated before the 2 resulting dataframes are combined to produce the single
# sample by variables (fauna at family level) matrix.

# Where family level dataset already created then proceed to Step 7

###############################################################################################
### STEP REF:       1                                                                      ###
###                                                                                        ###
### PAPER SECTION:  ALL                                                                    ###
###                                                                                        ###
### TASK:           Load the raw dataset                                                   ###
###############################################################################################

## Load data (created using script in 'C5922 FINAL SCRIPT BUILD DATASETV8.R')
dataall2=read.csv("DATA/C5922DATASET13022017.csv", header=T,na.strings=c("NA", "-","?","<null>"),
                  stringsAsFactors=F,check.names=FALSE)

## View dataset
View(dataall2)

## Remove 1st column (repeat of row labels)
dataall2 = dataall2[,-1]

## View dataset
View(dataall2)# check 1st column removed

## Check dataset dimensions
dim(dataall2)# 33198 13587

## Check column order
options(max.print=1000000)# to see all column names
names(dataall2) # Structure: Faunal data (A & B) followed by other variables

## Select only variables of interest
# 1:13450 Faunal data
# 13451 Order
# 13452 SampleCode
# 13453 SurveyName
# 13455 Latitude_WGS84
# 13456 Longitude_WGS84
# 13458 Gear
# 13460 Sieve
# 13461 Year
# 13462 Month
# 13465:13563 Sed data
# 13564 Sector
# 13565 Source
# 13569 PSASubSample
# 13572 Treatment
# 13575 Sal
# 13576 Temp
# 13577 Chla
# 13578 SPM
# 13579 Depth
# 13580 WOV
# 13581 AvCur
# 13582 Stress
# 13583 IncCol
# 13584 TopSieveEmpty
```

```
# 13585 Data
# 13586 Programme
# 13587 WentworthSuitability
dataall3=dataall2[,c(1:13450,13465:13563,13451:13453,13455:13456,13458,13460:13462,13564:13565,
                     13569,13572,13575:13587)]

## Check column names
names(dataall3)

## Check dataset dimensions
dim(dataall3) # 33198 (samples) 13575 (variables)

###################################################################################################
### STEP REF:       2                                                                           ###
###                                                                                             ###
### PAPER SECTION:  ALL                                                                         ###
###                                                                                             ###
### TASK:           Split Raw dataset (selected variables) into sections A & B                  ###
###                                                                                             ###
### NOTES:          The dataset is too large to manipulate as a whole, so it must be split      ###
###                 into2 chunks(A & B)                                                         ###
###################################################################################################

## Create a dataframe object 'dataparta' for Part A
dataparta=dataall3[1:21101,]# samples 1:21101

## Create a dataframe object 'datapartb' for Part B
datapartb=dataall3[21102:33198,]# samples 21102:33198

## Save parts A and B as .csv files
write.csv(dataparta,file = "DATA/dataparta.csv",row.names=TRUE)
write.csv(datapartb,file = "DATA/datapartb.csv",row.names=TRUE)

## Remove objects that are no longer required to free up memory
rm(dataall2,dataall3)
gc()
rm(dataparta,datapartb)
gc()

###################################################################################################
### STEP REF:       3                                                                           ###
###                                                                                             ###
### PAPER SECTION:  ALL                                                                         ###
###                                                                                             ###
### TASK:           Generate a taxon matrix (family level) for Part A                           ###
###                                                                                             ###
### NOTES:          If you experience memory problems close R and other programmes and start    ###
###                 from Step 3 (having set the working directory)                              ###
###################################################################################################

## Load the data for Part A
dataparta=read.csv("DATA/dataparta.csv", header=T,na.strings=c("NA", "-", "?","<null>"),
                   stringsAsFactors=F)

## View df 'dataparta'
View(dataparta)

## Remove 1st column
dataparta$X=NULL

## Check dimensions of Part A - should be 21101 13575
dim(dataparta)# it is

## View names for df 'dataparta'
options(max.print=1000000)# to see all column names
names(dataparta)

## Select only the faunal data. Note that this includes columns for additional taxa from part B
# (all with zero abundance)
dataparta2=dataparta[,1:13450]

## View df 'dataparta2
View(dataparta2)

## Change NAs to 0. NAs occur for taxa relating to part B
dataparta2[is.na(dataparta2)] <- 0
```

```
## Transpose the faunal data (keep column names)
t_dataparta = setNames(data.frame(t(dataparta2[,-1])), dataparta2[,1])

## Bring in aggregation data. Note that Part A includes full taxon list, hence use of the Part B
# Aggregation file
agg=read.csv("DATA/PARTBAGG05022017.csv", header=T,
             na.strings=c("NA", "-","?","<null>"),stringsAsFactors=F,check.names=FALSE)

## View aggreation file
View(agg)

## Join the aggregation file to data
alla=cbind(agg,t_dataparta)

## Remove unwanted taxa
alla2 = subset(alla, Include=='Y')

## Identify dimensions of df 'alla2'
dim(alla2)# note the total number of columns

## View df 'alla2'
View(alla2)# identify the col nos for Family and samples

## Take only the Family and sample columns
alla2fam=alla2[,c(19,31:21131)]

## Remove objects that are no longer required to free up memory
rm(alla)
gc()
rm(alla2)
gc()
rm(t_dataparta)
gc()
rm(dataparta)
gc()
rm(dataparta2)
gc()

## Aggregate data (sum) by family for multiple samples
famabundPARTA=aggregate(. ~ Family, alla2fam, sum)

## Remove objects that are no longer required to free up memory
rm(alla2fam)
gc()
rm(agg)
gc()

## Write file and close
write.csv(famabundPARTA,file = "DATA/famabundPARTA.csv",row.names=TRUE)

###################################################################################################
### STEP REF:       4                                                                           ###
###                                                                                             ###
### PAPER SECTION:  ALL                                                                         ###
###                                                                                             ###
### TASK:           Generate a taxon matrix (family level) for Part B                           ###
###################################################################################################

## Load the data for Part B
datapartb=read.csv("DATA/datapartb.csv", header=T,na.strings=c("NA", "-", "?","<null>"),
                   stringsAsFactors=F)

## Remove 1st column
datapartb$X=NULL

## View names for df 'datapartb'
options(max.print=1000000)# to see all column names
names(datapartb)

## Select only the faunal data
datapartb2=datapartb[,1:13450]

## Transpose the data (keep column names)
t_datapartb = setNames(data.frame(t(datapartb2[,-1])), datapartb2[,1])
```

```
## Bring in aggregation data
agg=read.csv("DATA/PARTBAGG05022017.csv", header=T,na.strings=c("NA", "-", "?","<null>"),
             stringsAsFactors=F)

## Join aggregation file to data
allb=cbind(agg,t_datapartb)

## Remove unwanted taxa
allb2 = subset(allb, Include=='Y')
names(allb2)

## Take only the Family col and the sample cols (NB Impossible to aggregate other factor cols)
allb2fam=allb2[,c(19,31:12127)]

## Remove objects that are no longer required to free up memory
rm(agg,allb,allb2,datapartb,datapartb2,t_datapartb)
gc()

## Sum by family for multiple samples
famabundPARTB=aggregate(. ~ Family, allb2fam, sum)

## Remove objects that are no longer required to free up memory
rm(allb2fam)
gc()

## Write file
write.csv(famabundPARTB,file = "DATA/famabundPARTB.csv",row.names=TRUE)

## Remove objects that are no longer required to free up memory
rm(famabundPARTA,famabundPARTB)
gc()

###################################################################################################
### STEP REF:       5                                                                           ###
###                                                                                             ###
### PAPER SECTION:  ALL                                                                         ###
###                                                                                             ###
### TASK:           Build family abundance matrix from Parts A & B                              ###
###################################################################################################

## Load data from Part A
famabundPARTA=read.csv("DATA/famabundPARTA.csv", header=T,na.strings=c("NA", "-","?","<null>"),
                       stringsAsFactors=F,check.names=FALSE)

## View df 'famabundPARTA'
View(famabundPARTA)

## Remove 1st column
famabundPARTA[1]=NULL

## Check dimensions of df 'famabundPARTA'
dim(famabundPARTA)#774 21102

## Load data from Part B
famabundPARTB=read.csv("DATA/famabundPARTB.csv", header=T,na.strings=c("NA", "-","?","<null>"),
                       stringsAsFactors=F,check.names=FALSE)

## Remove 1st column
famabundPARTB[1]=NULL

## View df 'famabundPARTB'
View(famabundPARTB)

## Check dimensions of df 'famabundPARTB'
dim(famabundPARTB)#774 12098

## Merge the dataframes 'famabundPARTA' and 'famabundPARTB'
total <- merge(famabundPARTA,famabundPARTB,by="Family")

##Check dimensions of df 'total'
dim(total)#774 33199

## View df 'total'. Note samples as columns
View(total)

## Transpose df 'total' so samples as rows and variables as columns
```

```
t_total = setNames(data.frame(t(total[,-1])), total[,1])

## Check orientation of df 't_total'
View(t_total)

## Check names of df 't_total'
names(t_total)

## Change row names (sample codes) to a column
t_total <- cbind(Sample = rownames(t_total), t_total)
rownames(t_total) <- NULL

## Check new column (Sample) added and old row names (Sample codes)removed
View(t_total)# all correct

## Write file
write.csv(t_total,file = "DATA/t_total.csv",row.names=TRUE)

###############################################################################################
### STEP REF:       6                                                                       ###
###                                                                                         ###
### PAPER SECTION:  ALL                                                                     ###
###                                                                                         ###
### TASK:           Add other data (sediments/metadata/other variables) to family abundance ###
###                 matrix                                                                  ###
###############################################################################################

## Load required dfs
dataparta=read.csv("DATA/dataparta.csv", header=T,na.strings=c("NA", "-", "?","<null>"),
                   stringsAsFactors=F)
datapartb=read.csv("DATA/datapartb.csv", header=T,na.strings=c("NA", "-", "?","<null>"),
                   stringsAsFactors=F)

## Remove 1st columns of dfs 'dataparta' and 'datapartb'
dataparta$X=NULL
datapartb$X=NULL

## View names of df 'dataparta'
names(dataparta)
names(datapartb)

## Select only the non-faunal data (i.e. meta and sediment data)
dataparta2other=dataparta[,c(1,13451:13575)]
datapartb2other=datapartb[,c(1,13451:13575)]

## Join together df 'dataparta2other' and 'datapartb2other' (other variables -  parts A and B)
datapartab2other=rbind(dataparta2other,datapartb2other)

## Merge faunal (family) matrix with other variables
data <- merge(t_total,datapartab2other,by="Sample")

## Check dimensions of df 'data1'
dim(data)# 33198 900

## Check names of df 'data1'
names(data)

## View df 'data1'
View(data)

## Save df 'data1' (Sample/variable matrix, Family level)
write.csv(data,file = "DATA/C5922DATASETFAM13022017.csv",row.names=TRUE)

## Remove objects that are no longer required to free up memory
rm(data,dataparta,dataparta2other,datapartab2other,datapartb,datapartb2other,famabundPARTA,
   famabundPARTB,t_total,total)
gc()

###############################################################################################
# # #                                   (B) LOAD DATASET                                  # # #
###############################################################################################

###############################################################################################
### STEP REF:       7                                                                       ###
###                                                                                         ###
### PAPER SECTION:  ALL                                                                     ###
```

```
###                                                                                          ###
### TASK:           Load dataset                                                             ###
###                                                                                          ###
### NOTES:          The dataset is a sample by variable matrix. Variables include faunal taxa ###
###                  (family), sediment data, metadata and other explanatory variables        ###
################################################################################################

## Read in data
data=read.csv("DATA/C5922DATASETFAM13022017.csv", header=T,na.strings=c("NA", "-","?","<null>"),
              stringsAsFactors=F,check.names=FALSE)

## Remove 1st col
data[1] <- NULL

## Check dataset dimensions
dim(data)#33198 900

## View dataset
View(data)

## Change variable data types (factors required for plotting).
data$Sieve = as.factor(data$Sieve)
data$Year2 = as.factor(data$Year)
data$Month = as.factor(data$Month)
data$Source = as.factor(data$Source)
data$Gear = as.factor(data$Gear)
data$Data = as.factor(data$Data)

## Number of Surveys
length(rle(sort(data$Survey))$values)# 777

## Number of samples by Survey
table(data$Survey)

################################################################################################
# # #                              (C) PREPARE MAPPING LAYERS                              # # #
################################################################################################

################################################################################################
### STEP REF:       8                                                                        ###
###                                                                                          ###
### PAPER SECTION:  ALL                                                                      ###
###                                                                                          ###
### TASK:           Create layers for use in maps                                            ###
################################################################################################
## Install packages
#install.packages("ggplot2")
#install.packages("rgdal")
#install.packages("maptools")
#install.packages("plyr")

## Call packages
library(ggplot2)
library(rgdal)
library(maptools)
library(plyr)

## Produce a high definition european coast map
# Load shapefile
eu <- readOGR("DATA","EUROPE")
eu@data$id <- rownames(eu@data)

## Create a data.frame from our spatial object
euDF <- fortify(eu, region = "id")

## Merge the "fortified" data with the data from our spatial object. Object euDF
# is a dataframe of the polygon coordinates.  This is combined with the other attribute
# data from object eu
euDF2 <- merge(euDF, eu@data, by = "id")

## Create layer for coastline including borders between UK countries
eubound = readOGR("DATA","EuropeLiteScoWal")
eubound@data$id = rownames(eubound@data)
eubound.points = fortify(eubound, region="id")
eubound.df = join(eubound.points, eubound@data, by="id")
```

```
## Create layer for 'Licensed' polygons
licence = readOGR("DATA","Aggregates_Licence_20151112")
licence@data$id = rownames(licence@data)
licence.points = fortify(licence, region="id")
licence.df = join(licence.points, licence@data, by="id")

## Create layer for 'Application' polygons
appl = readOGR("DATA","Aggregates_Application_20150813")
appl@data$id = rownames(appl@data)
appl.points = fortify(appl, region="id")
appl.df = join(appl.points, appl@data, by="id")

## Create layer for HUMBER AGGREGATE INTEREST
hum = readOGR("DATA","HUMBERLICANDAPP")
hum@data$id = rownames(hum@data)
hum.points = fortify(hum, region="id")
hum.df = join(hum.points, hum@data, by="id")

## Create layer for HUMBER SIZ
humSIZ = readOGR("DATA","H_SIZ_PSD_POLYGONS_UNION_2014")
humSIZ@data$id = rownames(humSIZ@data)
humSIZ.points = fortify(humSIZ, region="id")
humSIZ.df = join(humSIZ.points, humSIZ@data, by="id")

## Create layer for HUMBER APP AREA 492
humapp492 = readOGR("DATA","H_492_PIZ_APP")
humapp492@data$id = rownames(humapp492@data)
humapp492.points = fortify(humapp492, region="id")
humapp492.df = join(humapp492.points, humapp492@data, by="id")

## Create layer for ANGLIAN AGGREGATE INTEREST
ang = readOGR("DATA","ANGLIANLICANDAPP")
ang@data$id = rownames(ang@data)
ang.points = fortify(ang, region="id")
ang.df = join(ang.points, ang@data, by="id")

## Create layer for ANGLIAN SIZ
angSIZ = readOGR("DATA","A_SIZ_PSD_POLYGONS_UNION")
angSIZ@data$id = rownames(angSIZ@data)
angSIZ.points = fortify(angSIZ, region="id")
angSIZ.df = join(angSIZ.points, angSIZ@data, by="id")

## Create layer for THAMES AGGREGATE INTEREST
tha = readOGR("DATA","THAMESLICANDAPP")
tha@data$id = rownames(tha@data)
tha.points = fortify(tha, region="id")
tha.df = join(tha.points, tha@data, by="id")

## Create layer for THAMES SIZ
thaSIZ = readOGR("DATA","T_SIZ_PSD_POLYGONS_UNION_REV_2014")
thaSIZ@data$id = rownames(thaSIZ@data)
thaSIZ.points = fortify(thaSIZ, region="id")
thaSIZ.df = join(thaSIZ.points, thaSIZ@data, by="id")

## Create layer for THAMES AREA 501/2 SIZ
tha5012SIZ = readOGR("DATA","T_501_1_2_SIZ_PSD")
tha5012SIZ@data$id = rownames(tha5012SIZ@data)
tha5012SIZ.points = fortify(tha5012SIZ, region="id")
tha5012SIZ.df = join(tha5012SIZ.points, tha5012SIZ@data, by="id")

## Create layer for EEC AGGREGATE INTEREST
eec = readOGR("DATA","EECLICANDAPP")
eec@data$id = rownames(eec@data)
eec.points = fortify(eec, region="id")
eec.df = join(eec.points, eec@data, by="id")

## Create layer for EEC SIZs
eecSIZ = readOGR("DATA","EC_SIZ_PSD_POLYGONS_UNION_REV")
eecSIZ@data$id = rownames(eecSIZ@data)
eecSIZ.points = fortify(eecSIZ, region="id")
eecSIZ.df = join(eecSIZ.points, eecSIZ@data, by="id")

## Create layer for S. COAST AGGREGATE INTEREST
sc = readOGR("DATA","SCOASTLICANDAPP")
sc@data$id = rownames(sc@data)
sc.points = fortify(sc, region="id")
```

```
sc.df = join(sc.points, sc@data, by="id")

## Create layer for S. COAST SIZs
scSIZ = readOGR("DATA","SC_SIZ_PSD_POLYGONS_UNION")
scSIZ@data$id = rownames(scSIZ@data)
scSIZ.points = fortify(scSIZ, region="id")
scSIZ.df = join(scSIZ.points, scSIZ@data, by="id")

## Create layer for BRISTOL CHANNEL AGGREGATE INTEREST
bc = readOGR("DATA","BRISTOLCHANNELLICANDAPP")
bc@data$id = rownames(bc@data)
bc.points = fortify(bc, region="id")
bc.df = join(bc.points, bc@data, by="id")

## Create layer for BRISTOL CHANNEL SIZ
bcSIZ = readOGR("DATA","BC_SIZ2")
bcSIZ@data$id = rownames(bcSIZ@data)
bcSIZ.points = fortify(bcSIZ, region="id")
bcSIZ.df = join(bcSIZ.points, bcSIZ@data, by="id")

## Create layer for NORTH WEST AGGREGATE INTEREST
nw = readOGR("DATA","NORTHWESTLICANDAPP")
nw@data$id = rownames(nw@data)
nw.points = fortify(nw, region="id")
nw.df = join(nw.points, nw@data, by="id")

## Create layer for Area 457 SIZ (NORTH WEST REGION)
nw457SIZ = readOGR("DATA","AREA_457_PSD")
nw457SIZ@data$id = rownames(nw457SIZ@data)
nw457SIZ.points = fortify(nw457SIZ, region="id")
nw457SIZ.df = join(nw457SIZ.points, nw457SIZ@data, by="id")

## Create layer for Area 392 SIZ (PSD Zone)
nw392SIZ = readOGR("DATA","NW_392_SIZ_PSD_LICENCE_EXISTING")
nw392SIZ@data$id = rownames(nw392SIZ@data)
nw392SIZ.points = fortify(nw392SIZ, region="id")
nw392SIZ.df = join(nw392SIZ.points, nw392SIZ@data, by="id")

## Goodwin licence
goodwinlic = readOGR("DATA","GOODWIN LICENCE FINAL POLYGON")
goodwinlic@data$id = rownames(goodwinlic@data)
goodwinlic.points = fortify(goodwinlic, region="id")
goodwinlic.df = join(goodwinlic.points, goodwinlic@data, by="id")

## Create layer for Goodwin SIZ (10mg)
goodwinSIZ = readOGR("DATA","GoodwinSIZ")
goodwinSIZ@data$id = rownames(goodwinSIZ@data)
goodwinSIZ.points = fortify(goodwinSIZ, region="id")
goodwinSIZ.df = join(goodwinSIZ.points, goodwinSIZ@data, by="id")

## Defra DEM (bathy)
defra.dem = readOGR("DATA","DEFRADEMKC8")
defra.dem$id = rownames(defra.dem@data)
defra.dem.points = fortify(defra.dem, region="id")
defra.dem.df = join(defra.dem.points, defra.dem@data, by="id")
defra.dem.df$Depth = factor(defra.dem.df$Depth,levels(defra.dem.df$Depth)[c(1,2,5,8,9,10,3,6,11,
                                                                             4,7)])
levels(defra.dem.df$Depth)

## Create grey scale colour palette for use with bathy fill. 0=black, 1=white
grey=grey.colors(11, start = 0.95, end = 0.5,  alpha = NULL,gamma = 2.2)#, end=0.01

#################################################################################################
# # #                                  (D) DATASET SUMMARY                                  # # #
#################################################################################################

#################################################################################################
## STEP REF:        9                                                                         ###
##                                                                                            ###
## PAPER SECTION:  INTRODUCTION                                                               ###
##                                                                                            ###
## TASK:            Figure 1a (Study area showing locations of aggregate dredging interest)   ###
#################################################################################################

## Enter box coordinates
hum=data.frame(x1=c(0), x2=c(2.6),y1=c(53.1), y2=c(53.9),t=c('a'), r=c("A"))
```

```
ang=data.frame(x1=1.7, x2=2.5,y1=52.155, y2=52.9,t=c('a'), r=c("A"))
tha=data.frame(x1=1.3, x2=2.5,y1=51.5, y2=52.14,t=c('a'), r=c("A"))
good=data.frame(x1=1.3, x2=1.8,y1=51.0, y2=51.4,t=c('a'), r=c("A"))
sc=data.frame(x1=-1.9, x2=0.85,y1=50.3, y2=50.9,t=c('a'), r=c("A"))
ec=data.frame(x1=0, x2=1,y1=50.2, y2=50.6,t=c('a'), r=c("A"))
bc=data.frame(x1=-2.55, x2=-4.8,y1=51.1, y2=51.65,t=c('a'), r=c("A"))
nw=data.frame(x1=-3.2, x2=-3.8,y1=53.3, y2=53.8,t=c('a'), r=c("A"))

## Plot map
studyarea=ggplot()+
  geom_polygon(data=defra.dem.df, aes(x=long, y=lat, group=group,fill=Depth), size=0.15)+
  scale_fill_manual(values=grey)+
  geom_polygon(data = euDF2, aes(x=long, y=lat, group = group),fill="white",colour="black",
               size=0.15)+
  geom_polygon(data=goodwinlic.df, aes(x=long, y=lat, group=group), colour="orange",
               fill="orange",size=0.3,fill=NA)+#Goodwin Sands Extraction Area
  geom_polygon(data=ang.df, aes(x=long, y=lat, group=group), colour="limegreen",fill="limegreen",
               size=0.3,fill=NA)+#Anlian interest areas
  geom_polygon(data=tha.df, aes(x=long, y=lat, group=group), colour="yellow",fill="yellow",
               size=0.3,fill=NA)+#Thames interest areas
  geom_polygon(data=hum.df, aes(x=long, y=lat, group=group), colour="blue",fill="blue",
               size=0.3,fill=NA)+#Humber interest areas
  geom_polygon(data=humapp492.df, aes(x=long, y=lat, group=group), colour="blue",fill="blue",
               size=0.3,fill=NA)+#Humber Area 492 Application Area
  geom_polygon(data=eec.df, aes(x=long, y=lat, group=group), colour="purple",fill="purple",
               size=0.3,fill=NA)+#EEC interest areas
  geom_polygon(data=sc.df, aes(x=long, y=lat, group=group), colour="red",fill="red",
               size=0.3,fill=NA)+#S. Coast interest areas
  geom_polygon(data=bc.df, aes(x=long, y=lat, group=group), colour="deeppink2",fill="deeppink",
               size=0.3,fill=NA)+#Bristol Channel interest areas
  geom_polygon(data=nw.df, aes(x=long, y=lat, group=group), colour="brown", fill="brown",
               size=0.3,fill=NA)+#North-west interest areas
  labs(x="Longitude",y="Latitude")+
  coord_map(xlim = c(-10.7, 4),ylim = c(48.6, 62.7))+
  theme_bw(base_size=24)+
  geom_rect(data=hum, mapping=aes(xmin=x1, xmax=x2, ymin=y1, ymax=y2), color="blue",alpha=0,
            linetype=3,size=0.8)+# Humber region bounding box
  geom_rect(data=ang, mapping=aes(xmin=x1, xmax=x2, ymin=y1, ymax=y2), color="limegreen",alpha=0,
            linetype=3,size=0.8)+# Anglian region bounding box
  geom_rect(data=tha, mapping=aes(xmin=x1, xmax=x2, ymin=y1, ymax=y2), color="yellow",alpha=0,
            linetype=3,size=0.8)+# Thames region bounding box
  geom_rect(data=good, mapping=aes(xmin=x1, xmax=x2, ymin=y1, ymax=y2), color="orange",alpha=0,
            linetype=3,size=0.8)+# South Coast region bounding box
  geom_rect(data=sc, mapping=aes(xmin=x1, xmax=x2, ymin=y1, ymax=y2), color="red",alpha=0,
            linetype=3,size=0.8)+# South Coast region bounding box
  geom_rect(data=ec, mapping=aes(xmin=x1, xmax=x2, ymin=y1, ymax=y2), color="purple",
            alpha=0,linetype=3,size=0.8)+# East Channelregion bounding box
  geom_rect(data=bc, mapping=aes(xmin=x1, xmax=x2, ymin=y1, ymax=y2), color="deeppink2",
            alpha=0,linetype=3,size=0.8)+# Bristol Channelregion bounding box
  geom_rect(data=nw, mapping=aes(xmin=x1, xmax=x2, ymin=y1, ymax=y2), color="brown",
            alpha=0,linetype=3,size=0.8)+# North-west region bounding box
  annotate("text",x=c(2.33,-6,-1.1,-4.75,1,-8.5,-5.5),y=c(54.9,51.3,50.19,53.9,56.5,50,59.25),
         label=c("Dogger \n Bank", "Celtic \nSea","English Channel","Irish Sea","North Sea",
                 "Celtic \nShelf","Hebridean \n Shelf"),color="white", size=8)+
  annotate("text",x=c(-1.5),y=c(52.5),label=c("UK"),color="black", size=9)# UK label


fig1a=studyarea+guides(fill=FALSE)# removes bathy legend

###################################################################################################
### STEP REF:       10                                                                          ###
###                                                                                             ###
### PAPER SECTION:  2. METHODS\2.1 The dataset                                                  ###
###                                                                                             ###
### TASK:           Figure 1b (Sample locations and extent of submaps used in Figure 7)        ###
###################################################################################################

## Enter box coordinates
d=data.frame(x1=c(-1,0.8,0.8,-0.6,-2.7,-5,-5), x2=c(1.5,3.3,3.3,1.9,-0.2,-2.5,-2.5),
             y1=c(52.8,52.9,51.5,50.1,50,50.5,53.2), y2=c(54.3,54.4,53.0,51.6,51.5,52,54.7),
             t=c('a','b','c','d','e','f','g'), r=c("a","b","c","d","e","f","g"))

## Plot map
sam.pos=ggplot()+
  geom_polygon(data=defra.dem.df, aes(x=long, y=lat, group=group,fill=Depth), size=0.15)+
  scale_fill_manual(values=grey)+
```

```
  geom_point(data=data,aes(Longitude_WGS84,Latitude_WGS84),col="red", size=0.1, alpha=0.8)+
  geom_polygon(data = eubound, aes(x=long, y=lat, group = group),fill="white",colour="black",
               size=0.15)+
  labs(x="Longitude",y="Latitude")+
  coord_map(xlim = c(-10.7, 4),ylim = c(48.6, 62.7))+
  theme_bw(base_size=24)+
  theme(axis.title.y = element_blank())+ # remove y-axis title
  geom_rect(data=d, mapping=aes(xmin=x1, xmax=x2, ymin=y1, ymax=y2), color="black",alpha=0,
            size=0.3)+
  geom_text(data=d, aes(x=x1+(x2-x1)/2, y=y1+(y2-y1)/2, label=r), size=9, colour="black")+
  annotate("text",x=c(-1,-3.9,-3.6,-6.8),y=c(52.2,56.8,52.55,54.6),
           label=c("England", "Scotland","Wales", "Northern \n Ireland"),color="dark grey",
           size=7.2, angle=c(0,0,90,0))# Country labels

fig1b=sam.pos+ theme(legend.key.size=unit(1,"cm"))+ labs(fill="Depth (m)")

#################################################################################################
### STEP REF:       11                                                                        ###
###                                                                                           ###
### PAPER SECTION:  INTRODUCTION                                                              ###
###                 METHODS \ The dataset                                                     ###
###                                                                                           ###
### TASK:           Figure 1 Parts a & b combined (study area and sample locations)           ###
###                                                                                           ###
### NOTES:          Use this code if a plot combining parts a and b is required               ###
#################################################################################################

require(cowplot)

## Save plot to an image file (png or tiff).
png("OUTPUTS/FIGURE 1.png",width=53, height=37, units="cm", res=600)
#tiff("OUTPUTS/FIGURE 1.tiff",width=53, height=37, units="cm", res=600)

plot_grid(fig1a,fig1b, labels = c("a)","b)"),nrow = 1,label_size = 24,rel_widths = c(1, 1.2))
dev.off()

#################################################################################################
### STEP REF:       12                                                                        ###
###                                                                                           ###
### PAPER SECTION:  METHODS \ The dataset                                                     ###
###                                                                                           ###
### TASK:           Prepare the data for Figure 11 (Dataset summary maps and histograms)      ###
#################################################################################################

## Identify required variables for faceting
names(data)

## Create a subset of the required data ("Latitude_WGS84","Longitude_WGS84","Sector","Source",
# "Data","Year","Month","Gear","Sieve"","Programme","Treatment")
facdat1=data[,c(878,879,884,885,898,882,883,880,881,899,887)]

## Check required variables are there
names(facdat1)# they are

## Melt data into form for faceting
library(reshape2)
facdat2=melt(facdat1,c("Latitude_WGS84","Longitude_WGS84"))

## Check col names of melted data
names(facdat2)

## Insert NAs into empty cells in col 'value'
facdat2$value[facdat2$value==""]<- NA

## Add new col for concatenated variable and value cols - this is necessary to ensure unique
# values in maps, otherwise a 1 for month and sieve size will have the same colour
facdat3=transform(facdat2,varval=paste0(variable,value))

## Check col names of DF 'facdat3'
#View(facdat3)

#################################################################################################
### STEP REF:       13                                                                        ###
###                                                                                           ###
### PAPER SECTION:  METHODS \ The dataset                                                     ###
###                                                                                           ###
```

```
### TASK:           Figure 11a (Dataset summary: Map of sample by factor)                    ###
################################################################################################

## Produce faceted maps based on variables in df 'facdat3'. Colours prescribed manually by
# identifying the number of levels in each plot (see dataframe factdata3$varval and histogram
# FIGURE 11b). Colours given using code below. Note that colours are ordered alpabetically by
# df factdata3$varval.  May need to change order of colours to match histograms.
levels(facdat3$varval)

## Return a specified number of colours.
scales::hue_pal()(12) # Enter required number of colours in brackets

## Use this code to allow facet labels to be modified (changing col 'Sieve' to 'Sieve (mm)')
labels=c(Sector="Sector",Source="Source",Data="Data",Year="Year",Month="Month",Gear="Gear",
         Sieve="Sieve (mm)",Programme="Programme",Treatment="Treatment")

## Plot maps
sam.fac=ggplot()+
  geom_polygon(data=defra.dem.df, aes(x=long, y=lat, group=group,fill=Depth), size=0.15)+
  scale_fill_manual(values=grey)+
  geom_point(data=facdat3,aes(Longitude_WGS84,Latitude_WGS84,col=factor(varval)), size=0.01,
             show.legend = FALSE, alpha=0.4)+
  geom_polygon(data = euDF2, aes(x=long, y=lat, group = group),fill="white",colour="black",
               size=0.15)+
  coord_map(xlim = c(-10.7, 3.6),ylim = c(48.6, 62.7))+
  labs(x="Longitude",y="Latitude")+
  theme_bw(base_size=17)+#was 15,16
  facet_wrap(~variable,labeller=labeller(variable=labels))+
  scale_colour_manual(values = c("#F8766D","#00BFC4","#F8766D","#CD9600","#7CAE00","#00BE67",
                                 "#00BFC4","#00A9FF","#C77CFF","#FF61CC","#F8766D","#C77CFF",
                                 "#F564E3","#FF64B0","#DE8C00","#B79F00","#7CAE00","#00BA38",
                                 "#00C08B","#00BFC4","#00B4F0","#619CFF","lightsteelblue1",
                                 "#F8766D","#E58700","#C99800","#A3A500","#6BB100","#00BA38",
                                 "#00BF7D","#00C0AF","lightsteelblue1","#00BCD8","#00B0F6",
                                 "#619CFF","#B983FF","#E76BF3","#FD61D1","#FF67A4","#F8766D",
                                 "#00BFC4","#F8766D","#00BFC4","lightsteelblue1","#F8766D",
                                 "#E58700","#C99800","#A3A500","#6BB100","#00BA38","#00BF7D",
                                 "#00C0AF","#00BCD8","#00B0F6","#619CFF","#B983FF","#E76BF3",
                                 "#FD61D1","#FF67A4","#F8766D","lightsteelblue1","#00BFC4",
                                 "#F8766D","#F17D52","#E9842C","#E08B00","#D69100","#CA9700",
                                 "#BC9D00","#ADA200","#9CA700","#87AC00","#6FB000","#4DB400",
                                 "#00B813","#00BB44","#00BD61","#00BF79","#00C08E","#00C1A2",
                                 "#00C0B4","#00BFC4","#00BDD4","#00B9E2","#00B5EE","#00AFF8",
                                 "#00A7FF","#4D9FFF","#7F96FF","#A18BFF","#BC81FF","#D177FF",
                                 "#E26EF7","#EE67EC","#F863DF","#FE61D0","#FF62BF","#FF65AD",
                                 "#FF6A9A","#FD7084","lightsteelblue1"))

sam.fac2=sam.fac+guides(fill=FALSE)# removes the bathy legend

## Save plot to an image file (png or tiff)
png("OUTPUTS/FIGURE 11a.png",width = 14.7,height = 18.8,units = "cm",res = 600, pointsize = 12)
#tiff("OUTPUTS/FIGURE 11a.tiff",width = 14.7,height = 18.2,units = "cm",res = 600,pointsize = 48)
sam.fac2
dev.off()

################################################################################################
### STEP REF:       14                                                                       ###
###                                                                                          ###
### PAPER SECTION:  METHODS \ The dataset                                                    ###
###                                                                                          ###
### TASK:           Figure 11b (Dataset summary: Histograms to accompany facet maps)         ###
################################################################################################

## Plot histograms to accompany above maps (samples by factor)
p1h= ggplot(data, aes(x=reorder(Sector, -table(Sector)[Sector]),fill=Sector))+
  geom_bar()+
  ggtitle("Sector")+
  guides(fill=FALSE)+
  scale_x_discrete(labels=c("Industry","Government"))+
  theme(plot.title = element_text(size=14))+
  theme(axis.title.y = element_blank())+ # remove y-axis title
  theme(axis.title.x = element_blank())+ # remove x-axis title
  theme(axis.text.x = element_text(hjust=0.5,vjust=0.5, size=12))+
  theme(plot.title = element_text(hjust = 0.5))+
  theme(axis.text.y = element_text(hjust=1, size=12))# centres the plot title
p2h= ggplot(data, aes(x=reorder(Source, -table(Source)[Source]),fill=Source))+
```

```
  geom_bar()+
  ggtitle("Source")+
  guides(fill=FALSE)+
  scale_x_discrete(labels=c("MA","CEFAS","OW","DEFRA","NRW","EA", "NE","CCW","OG","SNH","JNCC",
                            "N","DOENI","PH","FRS"))+
  xlab(label="Source")+
  theme(plot.title = element_text(size=14))+
  theme(axis.title.x = element_blank())+ # remove x-axis title
  theme(axis.title.y = element_blank())+ # remove y-axis title
  theme(axis.text.x = element_text(angle=90, hjust=1,vjust=0.5, size=12))+
  theme(axis.text.y = element_text(hjust=1, size=12))
p3h= ggplot(data, aes(x=reorder(Data, -table(Data)[Data]),fill=Data))+
  geom_bar()+
  ggtitle("Data")+
  guides(fill=FALSE)+
  scale_x_discrete(labels=c("Existing","New"))+
  xlab(label="New")+
  theme(plot.title = element_text(size=14))+
  theme(axis.title.x = element_blank())+ # remove x-axis title
  theme(axis.title.y = element_blank())+ # remove y-axis title
  theme(axis.text.x = element_text(hjust=0.5,vjust=0.5, size=12))+
  theme(axis.text.y = element_text(hjust=1, size=12))
p5h= ggplot(data, aes(Year, fill=Year2))+
  geom_bar()+
  ggtitle("Year")+
  guides(fill=FALSE)+
  xlab(label="Year")+
  theme(plot.title = element_text(size=14))+
  theme(axis.title.x = element_blank())+ # remove x-axis title
  theme(axis.title.y = element_blank())+ # remove y-axis title
  theme(axis.text.x = element_text(angle=90, hjust=1,vjust=0.5, size=12))+
  theme(axis.text.y = element_text(hjust=1, size=12))
p6h= ggplot(data, aes(Month, fill=Month))+
  geom_bar()+
  scale_fill_hue(na.value = "lightsteelblue1")+
  ggtitle("Month")+
  guides(fill=FALSE)+
  xlab(label="Month")+
  theme(plot.title = element_text(size=14))+
  theme(axis.title.x = element_blank())+ # remove x-axis title
  theme(axis.title.y = element_blank())+ # remove y-axis title
  theme(axis.text.x = element_text(angle=90, hjust=1,vjust=0.5, size=12))+
  theme(axis.text.y = element_text(hjust=1, size=12))
p7h= ggplot(data, aes(x=reorder(Gear, -table(Gear)[Gear]),fill=Gear))+
  geom_bar()+
  ggtitle("Gear")+
  guides(fill=FALSE)+
  xlab(label="Gear")+
  theme(plot.title = element_text(size=14))+
  theme(axis.title.x = element_blank())+ # remove x-axis title
  theme(axis.title.y = element_blank())+ # remove y-axis title
  theme(axis.text.x = element_text(angle=90, hjust=1,vjust=0.5, size=12))+
  theme(axis.text.y = element_text(hjust=1, size=12))
p8h= ggplot(data, aes(x=reorder(Sieve, -table(Sieve)[Sieve]),fill=Sieve))+
  geom_bar()+
  scale_fill_hue(na.value = "lightsteelblue1")+
  ggtitle("Sieve (mm)")+
  guides(fill=FALSE)+
  xlab(label="Sieve (mm)")+
  theme(plot.title = element_text(size=14))+
  theme(axis.title.x = element_blank())+ # remove x-axis title
  theme(axis.title.y = element_blank())+ # remove y-axis title
  theme(axis.text.x = element_text(angle=90, hjust=1,vjust=0.5, size=12))+
  theme(axis.text.y = element_text(hjust=1, size=12))
p9h= ggplot(data, aes(x=reorder(Programme, -table(Programme)[Programme]),fill=Programme))+
  geom_bar()+
  scale_fill_hue(na.value = "lightsteelblue1")+
  ggtitle("Programme")+
  guides(fill=FALSE)+
  xlab(label="Programme")+
  theme(plot.title = element_text(size=14))+
  theme(axis.title.x = element_blank())+ # remove x-axis title
  theme(axis.title.y = element_blank())+ # remove y-axis title
  theme(axis.text.x = element_text(angle=90, hjust=1,vjust=0.5, size=12))+
  theme(axis.text.y = element_text(hjust=1, size=12))
p10h= ggplot(data, aes(x=reorder(Treatment, -table(Treatment)[Treatment]),fill=Treatment))+
```

```
  geom_bar()+
  scale_fill_hue(na.value = "lightsteelblue1")+
  ggtitle("Treatment")+
  guides(fill=FALSE)+
  scale_x_discrete(labels=c("Ref","Imp","NA"))+
  xlab(label="Treatment")+
  theme(plot.title = element_text(size=14))+
  theme(axis.title.x = element_blank())+ # remove x-axis title
  theme(axis.title.y = element_blank())+ # remove y-axis title
  theme(axis.text.x = element_text(hjust=0.5,vjust=0.5, size=12))+
  theme(axis.text.y = element_text(hjust=1, size=12))

## Save plot to an image file (png of tiff)
png("OUTPUTS/FIGURE 11b.png", width =1000, height=991)
#tiff("OUTPUTS/FIGURE 11b.tiff", width =1000, height=991)

## Combine individual plots into one plot
require(cowplot)
histo=plot_grid(p1h,p2h,p3h,p5h,p6h,p7h,p8h,p9h,p10h, align = "hv")
histo
dev.off()

###################################################################################################
### STEP REF:       15                                                                          ###
###                                                                                             ###
### PAPER SECTION:  METHODS \ The dataset                                                       ###
###                                                                                             ###
### TASK:           Figure 11 Parts a & b combined (Combined plot of sample by factor with      ###
###                 accompanying histograms)                                                    ###
###                                                                                             ###
### NOTES:          Use this code if a plot combining parts a and b is required                 ###
###################################################################################################

require(cowplot)

## Save plot to an image file (png or tiff). Specify number of rows using 'nrow=4'
png("OUTPUTS/FIGURE 11.png",width=53, height=37, units="cm", res=600)
#tiff("OUTPUTS/FIGURE 11.tiff",width=53, height=37, units="cm", res=600)
plot_grid(sam.fac2,histo, labels = c("a)","b)"),nrow = 1,rel_widths = c(1, 1.2),scale = 0.98,
          label_size = 24,hjust = c(-2,1), vjust = c(0.5,0.5)) #SCALE 0.95, RW 1:1.4
dev.off()

###################################################################################################
# # #                                  (E) FAUNAL ANALYSIS                                    # # #
###################################################################################################

###################################################################################################
### STEP REF:       16                                                                          ###
###                                                                                             ###
### PAPER SECTION:  METHODS \ Faunal data analysis \ Data subset                                ###
###                 RESULTS \ Faunal data analysis                                              ###
###                                                                                             ###
### TASK:           Prepare data for faunal analysis                                            ###
###                                                                                             ###
### NOTES:          Create a subset of comparable data for subsequent faunal analyses           ###
###################################################################################################

## Subset data by gear (all 0.1m2 grabs)
data2 = subset(data, Gear=="MHN" | Gear=="DG" | Gear=="VV" | Gear=="SM")

## Check dimensions of df 'data2'
dim(data2)# 32044 900

## subset by sieve size (1mm only)
data3 = subset(data2, Sieve=='1')

## Check dimensions of df 'data3'
dim(data3)# 27622 900

## Check names of df 'data3'to identify faunal data columns
names(data3) # 2:775

## Remove samples from the faunal data (df data3) where no fauna present
data4 = data3[ rowSums(data3[,2:775])!=0, ]

## Check dimensions of df 'data4'
```

```
dim(data4)#27432 900

## Identify (usinig the .csv file) variables with no abundance
#write.csv(data4,file = "OUTPUTS/data4.csv",row.names=TRUE)

## Remove variables with no abund (got list from csv file). There will be taxa
# with no abund as some samples with taxa present have been deleted in gear step above
data4.5 <- data4[-c(4,21,32,40,47,57,100,153,157,167,172,175,177,200,204,207,213,237,246,
                    252,283,294,313,325,362,376,379,381,410,413,414,417,418,426,428,430,446,
                    448,459,460,469,474,485,486,489,505,508,509,528,556,558,575,578,598,601,
                    603,605,607,630,633,638,647,675,687,705,709,726,734,760,765,775)]

## Check dimensions of df 'data4.5'
dim(data4.5)# 27432 829

## Show names of df 'data4.5'
names(data4.5)

## Faunal subset (ie remove Sample,Latitude_WGS84, Longitude_WGS84, month and year)
data5=data4.5[,2:704]

## Check dimensions of df 'data5'
dim(data5) #27432 703

## Check df 'data5' is just the faunal data
names(data5)# it is

## Change class of df data5 to a matrix
data6=data.matrix(data5)

## Create a df 'pos' for Sample, Latitude_WGS84 and Longitude_WGS84
pos=data4.5[,c(1,807:808)]

## Check names of df 'pos'
names(pos)

###################################################################################################
### STEP REF:       17                                                                          ###
###                                                                                             ###
### PAPER SECTION:  METHODS \ Faunal data analysis \ Univariate indices                         ###
###                 RESULTS \ Faunal data analysis \ Univariate indices                         ###
###                                                                                             ###
### TASK:           Generate univariate faunal summary measures                                 ###
###################################################################################################

#install.packages("vegan")

## Call library
library(vegan)

## Calculate univariate summary measures based on faunal abundance data in df 'data5'
Richness = specnumber(data5) # Species Richness(S)
Abundance=rowSums(data5) # Abundance

####################################################################################################
### STEP REF:       18                                                                           ###
###                                                                                              ###
### PAPER SECTION:  METHODS \ Faunal data analysis \ Univariate indices                          ###
###                 RESULTS \ Faunal data analysis \ Univariate indices                          ###
###                                                                                              ###
### TASK:           Figure 2 (Richness and Abundance Maps)                                       ###
####################################################################################################

## Combine coordinates, Richness and Abundance into one dataframe
univmeascoord=cbind(pos,Richness,Abundance)

## Change Richness to numeric value
univmeascoord$Richness=as.numeric(univmeascoord$Richness)

## Drop the Sample column
univmeascoord$Sample <- NULL

## Change values to rank order for each column
univmeascoord$Richness=rank(univmeascoord$Richness)
univmeascoord$Abundance=rank(univmeascoord$Abundance)
```

```
## Change col names so 'Richness' becomes 'Richness (Family)'
colnames(univmeascoord) <- c("Latitude_WGS84", "Longitude_WGS84", "Richness (Family)",
                             "Abundance")

## Reshape data into long format for faceting
#install.packages("reshape2")
library(reshape2)
univmeascoord2=melt(univmeascoord,c("Latitude_WGS84","Longitude_WGS84"))

## Call colouRamps for maps
#install.packages("colorRamps")
require( colorRamps )

## Produce map
RichAbun.plot= ggplot()+
  geom_polygon(data=defra.dem.df, aes(x=long, y=lat, group=group,fill=Depth))+
  scale_fill_manual(values=grey,name="Depth (m)",guide = FALSE)+ # Turn off DEM legend
  geom_point(data=univmeascoord2,aes(Longitude_WGS84,Latitude_WGS84,col=rank(value),
                                     size=value))+
  scale_size(range = c(0.04, 0.04),guide = FALSE)+
  labs(colour="Rank")+# to change point legend from 'rank(abundance)' to 'Rank'
  geom_polygon(data=euDF2, aes(x=long, y=lat, group=group),fill="white",colour="black",
             size=0.1)+
  coord_map(xlim = c(-10.7, 4),ylim = c(48, 62)) + #set x,y limits of plot
  theme_bw(base_size = 18)+# change all text to font size 16
  labs(x="Longitude",y="Latitude")+
  facet_wrap(~variable)+
  scale_color_gradientn(colors= matlab.like(50),breaks=c(10000,20000,30000,40000,50000),
                        labels=c(5000,10000,15000,20000,25000))# Add 'guide=FALSE' to remove
#legend

## Save plot to an image file (png or tiff)
png("OUTPUTS/FIGURE 2.png", width=33, height=23.331, units="cm", res=600, pointsize=24)
#tiff("OUTPUTS/FIGURE 2.tiff", width=33, height=23.331, units="cm", res=600, pointsize=24)

RichAbun.plot+ theme(legend.key.size = unit(0.75, "cm"))

dev.off()

## Save legend for use in Figure 7
legendrichsm <- get_legend(RichAbun.plot + theme(legend.key.size = unit(1.2, "cm"))+
                             theme(legend.text=element_text(size=20),
                                   legend.title=element_text(size=24)))
plot(legendrichsm)

###################################################################################################
### STEP:             19                                                                      ###
###                                                                                           ###
### PAPER SECTION:    METHODS \ Faunal data analysis \ Community analysis                     ###
###                   RESULTS \ Faunal data analysis \ Community analysis \ Clustering        ###
###                                                                                           ###
### TASK:             Transform faunal data prior to clustering                               ###
###################################################################################################

## Transform the data (fourth-root transformation)
datat=data6^(0.25)

###################################################################################################
### STEP:             20                                                                      ###
###                                                                                           ###
### PAPER SECTION:    METHODS \ Faunal data analysis \ Community analysis                     ###
###                   RESULTS \ Faunal data analysis \ Community analysis \ Clustering        ###
###                                                                                           ###
### TASK:             Figure 3a (Faunal 'elbow' plot)                                         ###
###                                                                                           ###
### NOTES:            This plot will be combined with others in step 46                       ###
###################################################################################################

## Determine the appropriate number of cluster groups using the Elbow plot method (see
# http://www.r-statistics.com/2013/08/k-means-clustering-from-r-in-action/)
wssplot <- function(datat, nc=50, seed=1234){
  wss <- (nrow(datat)-1)*sum(apply(datat,2,var))
  for (i in 2:nc){
    set.seed(seed)
    wss[i] <- sum(kmeans(datat, algorithm="MacQueen",iter.max=100,nstart=25,centers=i)$withinss)}
  plot(1:nc, wss, type="b", xlab="Number of Clusters",
```

```
       ylab="Within groups sum of squares")}

## Save plot to an image file (png of tiff)
png("OUTPUTS/FIGURE 3a ELBOW PLOT.png",width = 15,height = 13,units = "cm", res = 800,
    pointsize = 12)
#tiff("OUTPUTS/FIGURE 3a ELBOW PLOT.tiff",width = 15,height = 13,units = "cm",res = 800,
#pointsize = 12)

## Set text size
par(cex=0.8)

## Generate plot
wssplot(datat)

## Add line to show position of elbow
abline(v=12,col="red")

dev.off()

###################################################################################################
### STEP:              21                                                                       ###
###                                                                                             ###
### PAPER SECTION:     METHODS \ Faunal Data analysis \ Community analysis                    ` ###
###                    RESULTS \ Faunal Data analysis \ Community analysis \ Clustering         ###
###                                                                                             ###
### TASK:              K-means clustering of faunal data                                        ###
###################################################################################################

## Perform Kmeans clusterinig of data. Results (cluster group) to the object 'results'
set.seed(1234)
results=kmeans(datat,12,algorithm="MacQueen",iter.max=100,nstart=25)

## Number of samples belonging to each cluster group
results$size

###################################################################################################
### STEP:              22                                                                       ###
###                                                                                             ###
### PAPER SECTION:     METHODS \ Faunal data analysis \ Community analysis                      ###
###                    RESULTS \ Faunal data analysis \ Community analysis \ Clustering         ###
###                                                                                             ###
### TASK:              Figure 3a Dendrogram (relationship between faunal clusters)              ###
###                                                                                             ###
### NOTES:             This plot will be combined with others in step 46                        ###
###################################################################################################

## Faunal data transformed and therefore on a reasonably equal footing. Calculate the
# absolute differences between cluster centres over all variables.

nclusters = 12

absdiff = matrix(0, nrow=nclusters, ncol=nclusters)
centers = results$centers

for (j in 1:nclusters) {
  for (k in 1:nclusters) {
    absdiff[j,k] = sum(abs(centers[j,] - centers[k,]))
  }
}

d=round(absdiff, 1)

## Find distance matrix
d1 <- dist(as.matrix(d))

## Apply hirarchical clustering
hc <- hclust(d1)

## Save plot of dendrogram as an image file(png or tiff)
png("OUTPUTS/FIGURE 3a DENDRO.png", width = 15, height = 13, units = "cm", res = 800,
    pointsize = 12)
#tiff("OUTPUTS/FIGURE 3a DENDRO.tiff", width = 15, height = 13, units = "cm", res = 800,
#pointsize = 12)

## Convert hclust into a dendrogram and plot
hcd <- as.dendrogram(hc)
```

```
## Reduce label size of cluster labels 1-12
par(cex=0.8)

## Plot dendrogram
plot(hcd, ylab = "Height")

## Add circle symbols below each leaf of the dendrogram. Define colours.
col.circle=c("blue2","cyan1","#05aae1","plum2","darkorchid3","green3","palegreen1","#b40202",
             "red1","darkorange","yellow","#b4b404")

## Add symbols
symbols(1:12, rep(-18, 12), circles=rep(1, 12), add=TRUE, inches=.08,fg=col.circle,
        bg=col.circle, xpd=TRUE)

## Add cluster group labels
axis(1,at=seq(1,12,by=1),labels=c("A1","A2a","A2b","B1a","B1b","C1a","C1b","D1","D2a","D2b",
                                 "D2c","D2d"),pos=-20,cex.axis=0.8,lty = 0,cex.axis=1)
dev.off()

###############################################################################################
### STEP REF:       23                                                                      ###
###                                                                                         ###
### PAPER SECTION:  METHODS \ Faunal data analysis \ Community analysis                     ###
###                 RESULTS \ Faunal data analysis \ Community analysis \ Clustering        ###
###                                                                                         ###
### TASK:           Create a dataframe for producing faunal cluster maps                    ###
###############################################################################################

## Add cluster group from kmeans results file to df 'pos' which includes 'Sample',
# 'Latitude_WGS84' and 'Longitude_WGS84'
faunal.cluster=cbind(pos,results$cluster)

## Change name of col 'results$cluster' to 'ClusterNum'
names(faunal.cluster)[4]<-paste("ClusterNum")

## Add a new empty col 'FaunalCluster' to df 'faunal.cluster
faunal.cluster["FaunalCluster"]=NA

## Populate FaunalCluster col with new names (see dendrogram from Step 21)
faunal.cluster$FaunalCluster[faunal.cluster$ClusterNum == 11] <- "A1"
faunal.cluster$FaunalCluster[faunal.cluster$ClusterNum == 1]<- "A2a"
faunal.cluster$FaunalCluster[faunal.cluster$ClusterNum == 8] <- "A2b"
faunal.cluster$FaunalCluster[faunal.cluster$ClusterNum == 3]<- "B1a"
faunal.cluster$FaunalCluster[faunal.cluster$ClusterNum == 7] <- "B1b"
faunal.cluster$FaunalCluster[faunal.cluster$ClusterNum == 4] <- "C1a"
faunal.cluster$FaunalCluster[faunal.cluster$ClusterNum == 5] <- "C1b"
faunal.cluster$FaunalCluster[faunal.cluster$ClusterNum == 12] <- "D1"
faunal.cluster$FaunalCluster[faunal.cluster$ClusterNum == 2] <- "D2a"
faunal.cluster$FaunalCluster[faunal.cluster$ClusterNum == 10] <- "D2b"
faunal.cluster$FaunalCluster[faunal.cluster$ClusterNum == 6] <- "D2c"
faunal.cluster$FaunalCluster[faunal.cluster$ClusterNum == 9]<- "D2d"

###############################################################################################
### STEP REF:       24                                                                      ###
###                                                                                         ###
### PAPER SECTION:  METHODS \ Faunal data analysis \ Community analysis                     ###
###                 RESULTS \ Faunal data analysis \ Community analysis \ Clustering        ###
###                                                                                         ###
### TASK:           Figure 4a (Map of faunal cluster distribution with all samples)         ###
###############################################################################################

## Produce map
p2= ggplot()+
  geom_polygon(data=defra.dem.df, aes(x=long, y=lat, group=group,fill=Depth))+
  scale_fill_manual(values=grey,name="Depth (m)",guide=FALSE)+
  geom_point(data=faunal.cluster,aes(Longitude_WGS84,Latitude_WGS84,col=FaunalCluster),
             size=0.45,show.legend = TRUE)+
  geom_polygon(data = euDF2, aes(x=long, y=lat, group = group),fill="white",colour="black",
               size=0.1)+
  scale_colour_manual(values = c("blue2","cyan1","#05aae1","plum2","darkorchid3","green3",
                                 "palegreen1","#b40202","red1","darkorange","yellow",
                                 "#b4b404"),name="Cluster")+
  guides(colour = guide_legend(override.aes = list(size=3)))+ # Change size of legend dots
  coord_map(xlim = c(-10.7, 4),ylim = c(48, 62))+ #set x,y limits of plot
  theme_bw(base_size = 24)+
```

```
  labs(x="Longitude",y="Latitude")

fig4a=p2+theme(legend.key.size = unit(1, "cm"))+
  guides(colour = guide_legend(override.aes = list(size=6)))

## Save plot to an image file (png or tiff)
png("OUTPUTS/FIGURE 4a.png",width = 29.7,height = 42,units = "cm", res = 600,
    pointsize = 48)
#tiff("OUTPUTS/FIGURE 4a.tiff",width = 29.7,height = 42,units = "cm",res = 600,pointsize = 48)
fig4a
dev.off()

## Save legend for use in Figure 7
legendfclus <- get_legend(p2 + theme_bw(base_size=24)+ guides(colour = guide_legend(override.aes =
list(size=8))))
plot(legendfclus)

###################################################################################################
### STEP REF:       25                                                                          ###
###                                                                                             ###
### PAPER SECTION:  METHODS \ Faunal Data analysis \ Community analysis                         ###
###                 RESULTS \ Faunal Data analysis \ Community analysis\ Clustering             ###
###                                                                                             ###
### TASK:           Figure 4b (Map of faunal cluster distribution by faunal cluster group)      ###
###################################################################################################

## Produce map
p6= ggplot()+
  geom_polygon(data=defra.dem.df, aes(x=long, y=lat, group=group,fill=Depth), size=0.15)+
  scale_fill_manual(values=grey)+
  geom_point(data=faunal.cluster,aes(Longitude_WGS84,Latitude_WGS84,col=FaunalCluster),
             size=0.15,show.legend = FALSE)+
  geom_polygon(data = euDF2, aes(x=long, y=lat, group = group),fill="white",colour="black",
               size=0.15)+
  coord_map(xlim = c(-10.7, 4),ylim = c(48, 62)) + #set x,y limits of plot
  theme_bw(base_size=24)+
  scale_colour_manual(values = c("blue2","cyan1","#05aae1","plum2","darkorchid3","green3",
                                 "palegreen1","#b40202","red1","darkorange","yellow",
                                 "#b4b404"),name="Cluster")+
  guides(colour = guide_legend(override.aes = list(size=5)))+ # Change size of legend dots
  #(were too small)
  labs(x="Longitude",y="Latitude")+
  facet_wrap(~FaunalCluster)

fig4b=p6+guides(fill=FALSE)# remove bathy legend

## Save plot to an image file (png or tiff)
png("OUTPUTS/FIGURE 4b.png",width = 29.7,height = 42,units = "cm",res = 600,pointsize = 12)
#tiff("OUTPUTS/FIGURE 4b.tiff",width = 29.7,height = 42,units = "cm",res = 600,pointsize = 12)
fig4b
dev.off()

###################################################################################################
### STEP REF:       26                                                                          ###
###                                                                                             ###
### PAPER SECTION:  RESULTS \ Faunal Data analysis \ Community Analysis                         ###
###                                                                                             ###
### TASK:           Figure 4 Parts a) & b) combined                                             ###
###                                                                                             ###
### NOTES:          Use this code to combine parts 4a and 4b                                    ###
###################################################################################################

require(cowplot)

## Save plot to an image file (png or tiff).

png("OUTPUTS/FIGURE 4.png",width=58, height=37, units="cm", res=800)
#tiff("OUTPUTS/FIGURE 4.tiff",width=58, height=37, units="cm", res=800)
plot_grid(fig4a,fig4b, labels = c("a)","b)"),nrow = 1,label_size = 24)
dev.off()

###################################################################################################
### STEP:           27                                                                          ###
###                                                                                             ###
### PAPER SECTION:  METHODS \ Faunal data analysis \ Faunal distribution within areas of        ###
###                 aggregate industry interest                                                 ###
```

```
###                 RESULTS \ Faunal data analysis \ Faunal distribution within areas of      ###
###                 aggregate industry interest                                                ###
###                                                                                            ###
### TASK:           Figure 7 (Faunal cluster distribution submaps)                            ###
###                                                                                            ###
### NOTES:          Run code for individual submap areas a-g. Change filename as appropriate  ###
###                 Also remember to create another plot object 'faunalsubmapcluster(a) for   ###
###                 use in step 29                                                             ###
################################################################################################

## Create a df for location points. Positions are for the following locations:Eastbourne,
# Dungeness,Southampton,Portsmouth(4),Bournemouth(5),Skegness (6),Hunstanton (7),Bridlington(8),
# Grimsby,Lowestoft,Great Yarmouth,Swansea, Worthing(13), Weymouth, Harwich,Aldeburgh,Dover,
# Ramsgate,Ilfracombe,Bude,Tenby,Liverpool,Blackpool,Rhyl,Whitehaven and Cromer
place<-data.frame(long=c(0.95,0.31,-1.423,-1.07,-1.906,0.329,0.53,-0.246,-0.073,1.73,1.718,
                         -3.966,-3.164,-0.393,-2.468,1.254,1.577,1.309,1.393,-4.13,-4.541,
                         -4.722,-2.976,-3.031,-3.483,-3.58,1.3019),
                  lat=c(50.925,50.79,50.934,50.815,50.733,53.206,52.949,54.061,53.560,52.494,
                        52.629,51.637,51.491,50.825,50.638,51.934,52.136,51.131,51.34,51.199,
                        50.828,51.683,53.436,53.821,53.312,54.544,52.9314))

## Produce maps for each submap area. Select appropriate sub area in 'coord_map' step below.
# Use # to hide options not in use.
faunalsubmap= ggplot()+
  geom_polygon(data=defra.dem.df, aes(x=long, y=lat, group=group,fill=Depth), size=0.15)+
  scale_fill_manual(values=grey)+
  geom_point(data=faunal.cluster,aes(Longitude_WGS84,Latitude_WGS84,col=FaunalCluster),
             size=0.55,show.legend = TRUE)+
  geom_polygon(data = euDF2, aes(x=long, y=lat, group = group),fill="white",colour="black",
               size=0.165)+
  geom_polygon(data=licence.df, aes(x=long, y=lat, group=group), colour="black",size=0.3,
               fill=NA)+#Licencsed Extraction Areas
  geom_polygon(data=appl.df, aes(x=long, y=lat, group=group), colour="black",size=0.3,
               fill=NA)+#Application Extraction Areas
  geom_polygon(data=humapp492.df, aes(x=long, y=lat, group=group), colour="black", size=0.3,
               fill=NA)+#Humber Area 492 Application Area
  geom_polygon(data=goodwinlic.df, aes(x=long, y=lat, group=group), colour="black", size=0.3,
               fill=NA)+#Goodwin Sands Extraction Area
  geom_polygon(data=humSIZ.df, aes(x=long, y=lat, group=group), colour="black", size=0.3,
               linetype=2,fill=NA)+#Humber SIZs
  geom_polygon(data=angSIZ.df, aes(x=long, y=lat, group=group), colour="black", size=0.3,
               linetype=2,fill=NA)+#Anglian SIZs
  geom_polygon(data=thaSIZ.df, aes(x=long, y=lat, group=group), colour="black", size=0.3,
               linetype=2,fill=NA)+#Thames SIZs
  geom_polygon(data=tha5012SIZ.df, aes(x=long, y=lat, group=group), colour="black", size=0.3,
               linetype=2,fill=NA)+#Thames Area 501/2 SIZ
  geom_polygon(data=scSIZ.df, aes(x=long, y=lat, group=group), colour="black", size=0.3,
               linetype=2,fill=NA)+#S.Coast SIZs
  geom_polygon(data=eecSIZ.df, aes(x=long, y=lat, group=group), colour="black", size=0.3,
               linetype=2,fill=NA)+#EEC SIZs
  geom_polygon(data=bcSIZ.df, aes(x=long, y=lat, group=group), colour="black", size=0.3,
               linetype=2,fill=NA)+#Bristol Channel SIZs
  geom_polygon(data=nw457SIZ.df, aes(x=long, y=lat, group=group), colour="black", size=0.3,
               linetype=2,fill=NA)+#Area 457 North West SIZ
  geom_polygon(data=nw392SIZ.df, aes(x=long, y=lat, group=group), colour="black", size=0.3,
               linetype=2,fill=NA)+#Area 392 North West SIZ
  geom_polygon(data=goodwinSIZ.df, aes(x=long, y=lat, group=group), colour="black", size=0.3,
               linetype=2,fill=NA)+#Goodwin SIZ
  coord_map(xlim = c(-1,1.5),ylim = c(52.8, 54.3))+ #submap a
  #coord_map(xlim = c(0.8,3.3),ylim = c(52.9, 54.4))+ #submap b
  #coord_map(xlim = c(0.8,3.3),ylim = c(51.5, 53.0))+ #submap c
  #coord_map(xlim = c(-0.6,1.9),ylim = c(50.1, 51.6))+ #submap d
  #coord_map(xlim = c(-2.7,-0.2),ylim = c(50, 51.5))+ #submap e
  #coord_map(xlim = c(-5,-2.5),ylim = c(50.5, 52))+ #submap f
  #coord_map(xlim = c(-5,-2.5),ylim = c(53.2, 54.7))+ #submap g
  theme_bw(base_size=18)+
  scale_colour_manual(values = c("blue2","cyan1","#05aae1","plum2","darkorchid3","green3",
                                 "palegreen1","#b40202","red1","darkorange","yellow",
                                 "#b4b404"),name="Cluster")+
  guides(colour = guide_legend(override.aes = list(size=3)))+ # Change size of legend dots
  labs(x="Longitude",y="Latitude")+
  annotate("text",x=c(0.81,0.16,0.785,-3.82,-2.94,-1.423,-1.079,-1.906,-0.393,-2.468,0.73,0.15,
                      -0.22,-0.42,1.55,1.45,1.10,1.40,1.2,1.21,-3.91,-3.164,-3.94,-4.44,-4.84,
                      -4.379,-2.81,-2.85,-3.483,-4.559,-3.37,1.15),
           y=c(50.97,50.83,51.497,51.33,50.64,50.974,50.865,50.773,50.865,50.668,52.945,53.206,
               53.560,54.061,52.494,52.629,51.934,52.136,51.131,51.34,51.67,51.535,51.185,50.828,
```

```
             51.683,53.288,53.436,53.821,53.275,54.20,54.544,52.9314),
          label=c("Dungeness","Eastbourne","Thames Estuary","Bristol Channel","Lyme Bay",
                  "Southampton","Portsmouth","Bournemouth","Worthing","Weymouth","Hunstanton",
                  "Skegness","Grimsby","Bridlington","Lowestoft","Great Yarmouth","Harwich",
                  "Aldeburgh","Dover","Ramsgate","Swansea","Cardiff","Ilfracombe","Bude",
                  "Tenby","Anglesey","Liverpool","Blackpool","Rhyl","Isle of Man","Whitehaven",
                  "Cromer"),color="black", size=3.5)+#was 3
  annotate("text",x=0,y=53.58,label="R. Humber", angle =-37, size=3.5)+#was 3
  annotate("text",x=-3.1,y=51.43,label="Severn Estuary", angle =47, size=3.5)+#was 3
  theme(plot.margin=unit(c(-0.6,0.5,-0.6,1),"cm"))+#add white space around the plot (T,R,B,L)
  theme(legend.position="none",axis.title.x=element_blank(),
                       axis.title.y=element_blank())+
  geom_point(data=place,aes(long,lat),colour="black",size=2)#place name points

## Create object for use in step 29 (remove # for appropriate name)
faunalsubmapclustera=faunalsubmap
#faunalsubmapclusterb=faunalsubmap
#faunalsubmapclusterc=faunalsubmap
#faunalsubmapclusterd=faunalsubmap
#faunalsubmapclustere=faunalsubmap
#faunalsubmapclusterf=faunalsubmap
#faunalsubmapclusterg=faunalsubmap

## Save plot to an image file (png or tiff)
png("OUTPUTS/FIGURE 7g CLUSTER.png", width = 14.7, height = 14.7, units = "cm",
    res = 600, pointsize = 12)
#tiff("OUTPUTS/FIGURE 7g CLUSTER.tiff", width = 14.7, height = 14.7, units = "cm",
#res = 600, pointsize = 12)
faunalsubmap
dev.off()

###################################################################################################
### STEP REF:       28                                                                        ###
###                                                                                           ###
### PAPER SECTION:  METHODS \ Faunal data analysis \ Faunal distribution within areas of      ###
###                 aggregate industry interest                                               ###
###                 RESULTS \ Faunal data analysis \ Faunal distribution within               ###
###                 areas of aggregate industry interest                                      ###
###                                                                                           ###
### TASK:           Figure 7 (Faunal richness submaps)                                        ###
###                                                                                           ###
### NOTES:          Run code for individual submap areas a-g. Change filename as              ###
###                 appropriate                                                               ###
###################################################################################################

## Call colouRamps for maps
require( colorRamps )

## Produce map. Select appropriate sub area in 'coord_map' step below. Use # to hide options
# not in use.
submap.rich=ggplot()+
  geom_polygon(data=defra.dem.df, aes(x=long, y=lat, group=group,fill=Depth), size=0.15)+
  scale_fill_manual(values=grey)+
  geom_point(data=pos, aes(Longitude_WGS84,Latitude_WGS84,col=rank(Richness)),size=0.6,
           show.legend = TRUE)+
  geom_polygon(data = euDF2, aes(x=long, y=lat, group = group),fill="white",colour="black",
             size=0.165)+
  geom_polygon(data=licence.df, aes(x=long, y=lat, group=group), colour="black",size=0.3,
             fill=NA)+#Licencsed Extraction Areas
  geom_polygon(data=appl.df, aes(x=long, y=lat, group=group), colour="black", size=0.3,
             fill=NA)+#Application Extraction Areas
  geom_polygon(data=humapp492.df, aes(x=long, y=lat, group=group), colour="black", size=0.3,
             fill=NA)+#Humber Area 492 Application Area
  geom_polygon(data=goodwinlic.df, aes(x=long, y=lat, group=group), colour="black", size=0.3,
             fill=NA)+# Goodwin Sands Extraction Area
  geom_polygon(data=humSIZ.df, aes(x=long, y=lat, group=group), colour="black", size=0.3,
             linetype=2,fill=NA)+#Humber SIZs
  geom_polygon(data=angSIZ.df, aes(x=long, y=lat, group=group), colour="black", size=0.3,
             linetype=2,fill=NA)+#Anglian SIZs
  geom_polygon(data=thaSIZ.df, aes(x=long, y=lat, group=group), colour="black", size=0.3,
             linetype=2,fill=NA)+#Thames SIZs
  geom_polygon(data=tha5012SIZ.df, aes(x=long, y=lat, group=group), colour="black", size=0.3,
             linetype=2,fill=NA)+#Thames Area 501/2 SIZ
  geom_polygon(data=scSIZ.df, aes(x=long, y=lat, group=group), colour="black", size=0.3,
             linetype=2,fill=NA)+#S.Coast SIZs
  geom_polygon(data=eecSIZ.df, aes(x=long, y=lat, group=group), colour="black", size=0.3,
```

```
              linetype=2,fill=NA)+#EEC SIZs
  geom_polygon(data=bcSIZ.df, aes(x=long, y=lat, group=group), colour="black", size=0.3,
              linetype=2,fill=NA)+#Bristol Channel SIZs
  geom_polygon(data=nw457SIZ.df, aes(x=long, y=lat, group=group), colour="black", size=0.3,
              linetype=2,fill=NA)+#Area 457 North West SIZ
  geom_polygon(data=nw392SIZ.df, aes(x=long, y=lat, group=group), colour="black", size=0.3,
              linetype=2,fill=NA)+#Area 392 North West SIZ
  geom_polygon(data=goodwinSIZ.df, aes(x=long, y=lat, group=group), colour="black", size=0.3,
              linetype=2,fill=NA)+#Goodwin SIZ
  coord_map(xlim = c(-1,1.5),ylim = c(52.8, 54.3))+ #submap a
  #coord_map(xlim = c(0.8,3.3),ylim = c(52.9, 54.4))+ #submap b
  #coord_map(xlim = c(0.8,3.3),ylim = c(51.5, 53.0))+ #submap c
  #coord_map(xlim = c(-0.6,1.9),ylim = c(50.1, 51.6))+ #submap d
  #coord_map(xlim = c(-2.7,-0.2),ylim = c(50, 51.5))+ #submap e
  #coord_map(xlim = c(-5,-2.5),ylim = c(50.5, 52))+ #submap f
  #coord_map(xlim = c(-5,-2.5),ylim = c(53.2, 54.7))+ #submap g
  theme_bw(base_size=18)+
  theme(plot.margin=unit(c(-0.6,0.5,-0.6,1),"cm"))+#add white space around the plot (T,R,B,L)
  labs(x="Longitude",y="Latitude")+
  scale_color_gradientn(colors= matlab.like(50))+
  theme(legend.position="none",axis.title.x=element_blank(),
                         axis.title.y=element_blank(),axis.text.y=element_blank())

## Create object for use in step 29 (remove # for appropriate name)
submap.richa=submap.rich
#submap.richb=submap.rich
#submap.richc=submap.rich
#submap.richd=submap.rich
#submap.riche=submap.rich
#submap.richf=submap.rich
#submap.richg=submap.rich

## Save plot to an image file (png or tiff)
png("OUTPUTS/FIGURE 7at DIVERSITY.png", width = 14.7, height = 14.7, units = "cm",
    res = 600, pointsize = 12)
#tiff("OUTPUTS/FIGURE 7g DIVERSITY.tiff", width = 14.7, height = 14.7, units = "cm",
#res = 600, pointsize = 12)
submap.rich
dev.off()

#################################################################################################
### STEP REF:       29                                                                        ###
###                                                                                           ###
### PAPER SECTION:  METHODS \ Faunal data analysis \ Temporal assessment                      ###
###                 RESULTS \ Faunal data analysis \ Community analysis\ Temporal assessment  ###
###                                                                                           ###
### TASK:           Figure 7 parts a-g                                                        ###
###                                                                                           ###
### NOTES:          Use this code if a plot combining parts a to g                            ###
#################################################################################################

require(cowplot)
library(grid)
library(gridExtra)

## Convert faunal cluster submaps to gtable
g.Fa <- ggplotGrob(faunalsubmapclustera)
g.Fb <- ggplotGrob(faunalsubmapclusterb)
g.Fc <- ggplotGrob(faunalsubmapclusterc)
g.Fd <- ggplotGrob(faunalsubmapclusterd)
g.Fe <- ggplotGrob(faunalsubmapclustere)
g.Ff <- ggplotGrob(faunalsubmapclusterf)
g.Fg <- ggplotGrob(faunalsubmapclusterg)

## Convert faunal richness submaps to gtable
g.Ra <- ggplotGrob(submap.richa)
g.Rb <- ggplotGrob(submap.richb)
g.Rc <- ggplotGrob(submap.richc)
g.Rd <- ggplotGrob(submap.richd)
g.Re <- ggplotGrob(submap.riche)
g.Rf <- ggplotGrob(submap.richf)
g.Rg <- ggplotGrob(submap.richg)

## Extract the first three widths (left margin, y lab, and y axis) from faunal cluster submaps
Fa.widths <- g.Fa$widths[1:3]
Fb.widths <- g.Fb$widths[1:3]
```

```
Fc.widths <- g.Fc$widths[1:3]
Fd.widths <- g.Fd$widths[1:3]
Fe.widths <- g.Fe$widths[1:3]
Ff.widths <- g.Ff$widths[1:3]
Fg.widths <- g.Fg$widths[1:3]

## Extract the first three widths (left margin, y lab, and y axis) from faunal richness submaps
Ra.widths <- g.Ra$widths[1:3]
Rb.widths <- g.Rb$widths[1:3]
Rc.widths <- g.Rc$widths[1:3]
Rd.widths <- g.Rd$widths[1:3]
Re.widths <- g.Re$widths[1:3]
Rf.widths <- g.Rf$widths[1:3]
Rg.widths <- g.Rg$widths[1:3]

## Determine max width from all submaps
max.widths <- unit.pmax(Fa.widths,Fe.widths,Fb.widths,Ff.widths,Ra.widths,Re.widths,Rb.widths,
                        Rf.widths,Rc.widths,Rg.widths,Fc.widths,Fg.widths,Fd.widths,Rd.widths)

## Assign max widths to gtable faunal cluster submaps
g.Fa$widths[1:3] <- max.widths
g.Fb$widths[1:3] <- max.widths
g.Fc$widths[1:3] <- max.widths
g.Fd$widths[1:3] <- max.widths
g.Fe$widths[1:3] <- max.widths
g.Ff$widths[1:3] <- max.widths
g.Fg$widths[1:3] <- max.widths

## Assign max widths to gtable faunal richness submaps
g.Ra$widths[1:3] <- max.widths
g.Re$widths[1:3] <- max.widths
g.Rb$widths[1:3] <- max.widths
g.Rf$widths[1:3] <- max.widths
g.Rc$widths[1:3] <- max.widths
g.Rg$widths[1:3] <- max.widths
g.Rd$widths[1:3] <- max.widths

## Plot column titles
title <- ggdraw() + draw_label("   Faunal Cluster Group                              Diversity
(Richness)                                Faunal Cluster Group
Diversity (Richness)", fontface='bold',size = 24)

## Use plot_grid to produce each row
rw1=plot_grid(g.Fa,g.Ra,g.Fe,g.Re, labels = c("a)","","e)",""), nrow = 1,align='h',
              label_size = 24,hjust = 0.04)
rw2=plot_grid(g.Fb,g.Rb,g.Ff,g.Rf, labels = c("b)","","f)",""), nrow = 1,align='h',
              label_size = 24,hjust = 0.04)
rw3=plot_grid(g.Fc,g.Rc,g.Fg,g.Rg, labels = c("c)","","g)",""), nrow = 1,align='h',
              label_size = 24,hjust = 0.04)
rw4=plot_grid(g.Fd,g.Rd, labels = c("d)","","",""), ncol = 4,align='h',
              label_size = 24,hjust = 0.04)

## Combine all 4 rows and header labels
rwall=plot_grid(title,rw1,rw2,rw3,rw4,ncol=1,rel_heights=c(0.07,1,1,1,1))

png("OUTPUTS/FIGURE 7.png",width=63, height=59.4, units="cm", res=500)#was w70 h66
#tiff("OUTPUTS/FIGURE 7.tiff",width=63, height=59.4, units="cm", res=500)
rwall+ draw_grob(legendfclus, 2.9/6, 0, 1/3, 0.26)+ draw_grob(legendrichsm, 4.4/6, 0, 1/3, 0.32)
# dec last no. moves it down
dev.off()

###############################################################################################
### STEP REF:       30                                                                      ###
###                                                                                         ###
### PAPER SECTION:  METHODS \ Faunal data analysis \ Univariate indices                     ###
###                 METHODS \ Faunal data analysis \ Community analysis                     ###
###                 RESULTS \ Faunal data analysis \ Univariate indices                     ###
###                 RESULTS \ Faunal data analysis \ Community analysis \ Clustering        ###
###                                                                                         ###
### TASK:           Table 1                                                                 ###
###                                                                                         ###
### EXPLANATION:    Calculate mean and sd for richness and abundance by faunal cluster group  ###
###############################################################################################

## Add univariate outputs to df 'faunal.cluster' which includes variables: Sample, Lat, Long,
# ClusterNum and FaunalCluster
```

```
data.sum.meas=cbind(faunal.cluster,Richness,Abundance)

## Check new columns added
head(data.sum.meas)

## Get means, standard deviations, min and max for table 3
library(plyr)
richmeansd=ddply(data.sum.meas,~FaunalCluster,summarise,mean=mean(Richness),sd=sd(Richness))
abundmeansd=ddply(data.sum.meas,~FaunalCluster,summarise,mean=mean(Abundance),sd=sd(Abundance))
richmax=ddply(data.sum.meas,~FaunalCluster,summarise,max=max(Richness))
richmin=ddply(data.sum.meas,~FaunalCluster,summarise,min=min(Richness))
abundmax=ddply(data.sum.meas,~FaunalCluster,summarise,max=max(Abundance))
abundmin=ddply(data.sum.meas,~FaunalCluster,summarise,min=min(Abundance))

## Identify the number of samples belonging to each FaunalCluster group
table(data.sum.meas$FaunalCluster)

## Bind results together
table1partb=cbind(richmax,richmin$min,richmeansd$mean,richmeansd$sd,abundmax$max,abundmin$min,
                  abundmeansd$mean,abundmeansd$sd)

## Change name of col 2 to 'richmax$max'
colnames(table1partb)[2] <- "richmax$max"

## Export df 'table1partb' to a .csv file
write.csv(table1partb,file = "OUTPUTS/TABLE 1 UNI INDICES.csv",
          row.names=FALSE)

###############################################################################################
### STEP REF:       31                                                                      ###
###                                                                                         ###
### PAPER SECTION:  METHODS \ Faunal data analysis \ Univariate indices                     ###
###                 METHODS \ Faunal data analysis \ Community analysis                     ###
###                 RESULTS \ Faunal data analysis \ Univariate indices                     ###
###                 RESULTS \ Faunal data analysis \ Community analysis \ Clustering        ###
###                                                                                         ###
### TASK:           Table 1                                                                 ###
###                                                                                         ###
### EXPLANATION:    Determine whether mean richness and abundance values are 'high', 'med'  ###
###                 or 'low'based on quantiles                                              ###
###############################################################################################

## Enter full range of values across all 12 faunal cluster groups (data in file
#'TABLE 1 UNI INDICES.csv' - see step 30)
x=c(69.6,52.4,57.6,64.0,43.5,30.9,43.9,40.4,23.6,26.9,8.1,19.0)# Richness
#x=c(1121.9,972.3,365.8,226.6,205.7,152.8,243.4,636.6,91.2,138.5,25.7,100.4)# Abundance

min=min(x)
max=max(x)
range=max(x)-min(x)
range
int=range/3
int
low.upper=min(x)+int
low.upper
high.lower=max(x)-int
high.lower
lims=c(min,low.upper,high.lower,max)
lims

## Determine limits (3 equal chunks High Med Low)
cut(x, quantile(x, c(0, 1/3, 2/3, 1)))

###############################################################################################
### STEP REF:       32                                                                      ###
###                                                                                         ###
### PAPER SECTION:  METHODS \ Faunal data analysis \ Community analysis                     ###
###                 RESULTS \ Faunal data analysis \ Community analysis\ Clustering         ###
###                                                                                         ###
### TASK:           Table 1 (Export data for Simper analysis in Primer 6)                   ###
###                                                                                         ###
### NOTES:          Faunal data in df 'data5'.  The factor for 'FaunalCluster' is in df     ###
###                 'faunal.cluster'.                                                       ###
###############################################################################################

## Add Sample code to df 'data5'
```

```
data4simper=cbind(faunal.cluster$Sample,data5)

## Change name of col 1 from 'faunal.cluster$Sample' to 'Sample'
colnames(data4simper)[1] <- "Sample"

## Export both objects as .csv files for use with PRIMER6
write.csv(data4simper,file = "OUTPUTS/DATAFORSIMPER.csv",row.names=FALSE)
write.csv(faunal.cluster,file = "OUTPUTS/FACTORFORSIMPER.csv",row.names=FALSE)

################################################################################################
### STEP REF:       33                                                                      ###
###                                                                                         ###
### PAPER SECTION:  METHODS \ Faunal data analysis \ Community analysis                     ###
###                 RESULTS \ Faunal data analysis \ Community analysis \ Clustering        ###
###                                                                                         ###
### TASK:           Undertake SIMPER analysis using the Primer 6 software (i.e. NOT in R)   ###
###                                                                                         ###
### NOTES:          Primer>Open>All Files>DATAFORSIMPER.csv>Open                            ###
###                 Data Type>Sample data>Next                                              ###
###                 Title: Uncheck>Shape: Sample as rows>Data Type: Abundance>Blank:        ###
###                       zero>Next                                                         ###
###                 Text delimiters:  Tab,Sapce, Comma (check all)>Finish                   ###
###                 Primer>right mouse click on data>Factors>Add>Add factor named:          ###
###                       FaunalCluster                                                     ###
###                 Excel>Open>FACTORFORSIMPER.csv. Copy Cluster values to clipboard and    ###
###                       paste into the Primer/FaunalCluster>OK                            ###
###                 Primer>Analyse>Pre-treatment>Transform(overall)>Fourth root>ok          ###
###                 Primer>Analyse>SIMPER>Design: One way>Factor A: FaunalCluster>Measure:  ###
###                       Bray-Curtis Similarity>ok                                         ###
################################################################################################

################################################################################################
### STEP REF:       34                                                                      ###
###                                                                                         ###
### PAPER SECTION:  METHODS \ Faunal data analysis \ Temporal assessment                    ###
###                 RESULTS \ Faunal data analysis \ Community analysis \ Temporal assessment ###
###                                                                                         ###
### TASK:           Figure 5a (Faunal cluster by year bin)                                  ###
################################################################################################

## Create a df for 'Latitude_WGS84','Longitude_WGS84', 'FaunalCluster' (cols 2,3 and 5 of df
# 'faunal.cluster'),'Year'and 'Month' (cols 811:812 of df 'data4.5')
year.data=cbind(faunal.cluster[,c(2,3,5)],data4.5[,811:812])

## Check names of df 'year.data'
names(year.data)

## Add new col 'YearBin'to df 'year.data'
year.data["YearBin"]=NA # add a new col

## Populate col 'YearBin' with data from col 'Year'
year.data$YearBin=year.data$Year

## Code to stop NA category being displayed in facet plot
year.data <- year.data[!is.na(year.data$Year), ]

## Update values in col 'YearBin' with bin name
year.data$YearBin[year.data$YearBin==1976]="1976-1999"
year.data$YearBin[year.data$YearBin==1989]="1976-1999"
year.data$YearBin[year.data$YearBin==1993]="1976-1999"
year.data$YearBin[year.data$YearBin==1994]="1976-1999"
year.data$YearBin[year.data$YearBin==1995]="1976-1999"
year.data$YearBin[year.data$YearBin==1996]="1976-1999"
year.data$YearBin[year.data$YearBin==1997]="1976-1999"
year.data$YearBin[year.data$YearBin==1998]="1976-1999"
year.data$YearBin[year.data$YearBin==1999]="1976-1999"
year.data$YearBin[year.data$YearBin==2000]="2000-2002"
year.data$YearBin[year.data$YearBin==2001]="2000-2002"
year.data$YearBin[year.data$YearBin==2002]="2000-2002"
year.data$YearBin[year.data$YearBin==2003]="2003-2005"
year.data$YearBin[year.data$YearBin==2004]="2003-2005"
year.data$YearBin[year.data$YearBin==2005]="2003-2005"
year.data$YearBin[year.data$YearBin==2006]="2006-2008"
year.data$YearBin[year.data$YearBin==2007]="2006-2008"
year.data$YearBin[year.data$YearBin==2008]="2006-2008"
year.data$YearBin[year.data$YearBin==2009]="2009-2011"
```

```
year.data$YearBin[year.data$YearBin==2010]="2009-2011"
year.data$YearBin[year.data$YearBin==2011]="2009-2011"
year.data$YearBin[year.data$YearBin==2012]="2012-2014"
year.data$YearBin[year.data$YearBin==2013]="2012-2014"
year.data$YearBin[year.data$YearBin==2014]="2012-2014"
year.data$YearBin[year.data$YearBin==2015]="2015-2016"
year.data$YearBin[year.data$YearBin==2016]="2015-2016"

## Produce map
year= ggplot()+
  geom_polygon(data=defra.dem.df, aes(x=long, y=lat, group=group,fill=Depth), size=0.05)+
  scale_fill_manual(values=grey)+
  geom_point(data=year.data,aes(Longitude_WGS84,Latitude_WGS84,col=FaunalCluster), size=0.3,
             show.legend = TRUE)+
  geom_polygon(data = euDF2, aes(x=long, y=lat, group = group),fill="white",colour="black",
               size=0.15)+
  coord_map(xlim = c(-10.7, 4),ylim = c(48, 62))+ #set x,y limits of plot
  theme_bw(base_size=24)+
  scale_colour_manual(values = c("blue2","cyan1","#05aae1","plum2","darkorchid3","green3",
                                 "palegreen1","#b40202","red1","darkorange","yellow",
                                 "#b4b404"),name="Cluster")+
  guides(colour = guide_legend(override.aes = list(size=5)))+ # Change size of legend dots
  labs(x="Longitude",y="Latitude")+
  facet_wrap(~YearBin)

fig5a=year+theme(legend.position="none")

## Save plot to an image file (png or tiff)
png("OUTPUTS/FIGURE 5a.png", width = 29.7, height = 42, units = "cm", res = 600,pointsize = 12)
#tiff("OUTPUTS/FIGURE 5a.tiff",width = 29.7,height = 42, units = "cm", res = 600,pointsize = 12)
fig5a
dev.off()

###################################################################################################
### STEP REF:       35                                                                          ###
###                                                                                             ###
### PAPER SECTION:  METHODS \ Faunal data analysis \ Temporal assessment                        ###
###                 RESULTS \ Faunal data analysis \ Community analysis\ Temporal assessment    ###
###                                                                                             ###
### TASK:           Figure 5b (Faunal cluster map faceted by season)                            ###
###################################################################################################

## Create a new df 'month.data' from df 'year.data' with just the required variables:
# 'Latitude_WGS84', 'Longitude_WGS84', 'FaunalCluster' and 'Month'
month.data=year.data[,c(1:3,5)]

## Code to stop NA category being displayed in facet plot
month.data <- month.data[!is.na(month.data$Month), ]

## Create a df 'season.data' which is a copy of the df 'month.data'
season.data=month.data

## Add a new col 'Season' to df 'season.data'
season.data["Season"]=NA

## Populate col 'Season' with data from col 'Month'
season.data$Season=season.data$Month

## Select only the variables of interest:'Latitude_WGS84', 'Longitude_WGS84',
# 'FaunalCluster' and 'Season'(i.e. remove col 'Month')
season.data=season.data[,c(1:3,5)]

## Need to change col Season from factor to numeric
season.data$Season=as.numeric(season.data$Season)

## Code to stop NA category being displayed in facet plot
season.data <- season.data[!is.na(season.data$Season), ]

## Update values in col 'Season' with bin name
season.data$Season[season.data$Season==1]="Dec-Feb"
season.data$Season[season.data$Season==2]="Dec-Feb"
season.data$Season[season.data$Season==3]="Mar-May"
season.data$Season[season.data$Season==4]="Mar-May"
season.data$Season[season.data$Season==5]="Mar-May"
season.data$Season[season.data$Season==6]="Mar-May"
season.data$Season[season.data$Season==7]="Jun-Aug"
```

```
season.data$Season[season.data$Season==8]="Jun-Aug"
season.data$Season[season.data$Season==9]="Jun-Aug"
season.data$Season[season.data$Season==10]="Sep-Nov"
season.data$Season[season.data$Season==11]="Sep-Nov"
season.data$Season[season.data$Season==12]="Dec-Feb"

## Produce map
season= ggplot()+
  geom_polygon(data=defra.dem.df, aes(x=long, y=lat, group=group,fill=Depth), size=0.15)+
  scale_fill_manual(values=grey,guide = FALSE)+
  geom_point(data=season.data,aes(Longitude_WGS84,Latitude_WGS84,col=FaunalCluster),
             size=0.2,show.legend = TRUE)+
  geom_polygon(data = euDF2, aes(x=long, y=lat, group = group),fill="white",colour="black",
               size=0.15)+
  coord_map(xlim = c(-10.7, 4),ylim = c(48, 62)) + #set x,y limits of plot
  theme_bw(base_size=24)+
  scale_colour_manual(values = c("blue2","cyan1","#05aae1","plum2","darkorchid3","green3",
                                 "palegreen1","#b40202","red1","darkorange","yellow",
                                 "#b4b404"),name="Cluster")+
  guides(colour = guide_legend(override.aes = list(size=3)))+ # Change size of legend dots
  labs(colour="Cluster")+# to change point legend from 'FaunalCluster' to 'Cluster'
  labs(x="Longitude",y="Latitude")+
  facet_wrap(~Season)

fig5b=season+theme(legend.key.size = unit(1, "cm"))+
  guides(colour = guide_legend(override.aes = list(size=6)))# reduces size of legend key

## Save plot to an image file (png or tiff)
png("OUTPUTS/FIGURE 5b.png", width = 29.7, height = 42, units = "cm", res = 600,pointsize = 12)
#tiff("OUTPUTS/FIGURE 5b.tiff",width = 29.7,height = 42, units = "cm", res = 600,pointsize = 12)
fig5b
dev.off()

#################################################################################################
### STEP REF:       36                                                                        ###
###                                                                                           ###
### PAPER SECTION:  METHODS \ Faunal data analysis \ Temporal assessment                      ###
###                 RESULTS \ Faunal data analysis \ Community analysis\ Temporal assessment  ###
###                                                                                           ###
### TASK:           Figure 5 Parts a & b combined (Faunal cluster distribution by year        ###
###                 and season)                                                               ###
###                                                                                           ###
### NOTES:          Use this code if a plot combining parts a and b is required               ###
#################################################################################################

require(cowplot)

## Save plot to an image file (png or tiff).
png("OUTPUTS/FIGURE 5.png",width=54, height=37, units="cm", res=800)
#tiff("OUTPUTS/FIGURE 5.tiff",width=66, height=37, units="cm", res=800)
plot_grid(fig5a,fig5b, labels = c("a)","b)"),nrow = 1,label_size = 24,rel_widths = c(1.1, 1))
dev.off()

#################################################################################################
### STEP REF:       37                                                                        ###
###                                                                                           ###
### PAPER SECTION:  METHODS \ Faunal Data Analysis \ Explaining patterns in faunal            ###
###                 distribution                                                              ###
###                 RESULTS \ Faunal Data Analysis \ Explaining patternsin faunal             ###
###                 distribution                                                              ###
###                                                                                           ###
### TASK:           Prepare data for analysis using best, adonis and dbRDA                    ###
#################################################################################################

## Create a copy of the raw data df 'data'
data4best=data

## See col names of df 'data4CCA'
names(data4best)

## Change bathy values to positive
data4best$Depth=abs(data4best$Depth)

## Change sieve data 'NA' values to zero. Sieve data in cols 776:784
data4best[, 776:874][is.na(data4best[, 776:874])] <- 0
```

```
## Add in cols for Mud, Sand and Gravel
data4best$Mud=rowSums(data4best[874:836])
data4best$Sand=rowSums(data4best[835:809])
data4best$Gravel=rowSums(data4best[808:776])

## Add col for total percent (across sediment sieves - should be 100%)
data4best$Tpercent=rowSums(data4best[902:904])

## Check dimension of df data4best
dim(data4best)#33198 905

## Check variables of correct class
str(data4best, list.len=ncol(data4best))

## Remove samples where Tpercent is not 100
data4best2 <- subset(data4best, Tpercent > 99 & Tpercent < 101)
dim(data4best2)# 27147 905

## Select data only where top sieve empty
data4best3=subset(data4best2, TopSieveEmpty=="Yes"|TopSieveEmpty=="NA")
dim(data4best3)# 24191 905

## Select data only where PSA taken as a subsample from the faunal grab
data4best4=subset(data4best3,PSASubSample=="Yes")
dim(data4best4)# 21353 905

## Select data only where sample taken using a comparable gear type.
data4best5=subset(data4best4, Gear=="MHN"|Gear=="DG"|Gear=="VV"|Gear=="SM")
dim(data4best5)# 20703 905

## Select data only were sample processed over a 1mm sieve
data4best6=subset(data4best5, Sieve=='1')
dim(data4best6)# 19908 905

## Select data only were samples include colonial taxa
data4best7=subset(data4best6, IncCol=="Yes")
dim(data4best7)# 17177 905

## Select only data from 'Reference' conditions
data4best8=subset(data4best7, Treatment=="R"|Treatment=="NA")
dim(data4best8)# 15203 905

## Select only the columns of interest from df 'faunal.cluster' (Sample & FaunalCluster)
names(faunal.cluster)
clustergrpsforbest=subset(faunal.cluster[,c(1,5)])
dim(clustergrpsforbest)#27432 2

# Add custer groupings to df 'data4CCA8'
data4best8$FaunalCluster<-clustergrpsforbest[match(data4best8$Sample,
                                                   clustergrpsforbest$Sample),2]

## Check faunalCluster added
names(data4best8)

## Remove samples from the faunal data (df data4CCA8) where no fauna present
data4best9 = data4best8[ rowSums(data4best8[,2:775])!=0, ]
dim(data4best9)#15127 906

## Identify (using the .csv file) variables with no abundance
write.csv(data4best9,file = "OUTPUTS/data4best9.csv",row.names=TRUE)

## Remove variables with no abund (got list from csv file). There will be taxa with no abund as
# some samples with taxa present have been deleted in gear step above#
data4best10 <- data4best9[-c(4,5,7,21,24,32,36,39,40,47,57,97,100,138,139,145,153,157,165,167,
                          169,172,175,177,178,195,200,204,207,213,223,229,237,246,250,252,
                          263,264,283,292,294,295,303,308,313,314,322,323,325,361,362,376,
                          379,380,381,384,402,408,410,413,414,417,418,426,428,430,436,446,
                          447,448,453,454,459,460,465,469,474,477,479,485,486,489,496,501,
                          505,508,509,514,528,536,556,557,558,575,576,578,598,601,603,605,
                          607,618,630,633,638,641,642,647,651,675,687,689,698,699,700,705,
                          709,717,726,734,742,743,760,765,775)]

dim(data4best10)#15127 781

## Remove rows where NA is present in one of the phy variable columns
data4best10v2=data4best10[complete.cases(data4best10[,c(753,763:770,777:779)]),]
```

```
## Check the number of available samples by faunal cluster group
table(data4best10v2$FaunalCluster)# Answer is 297 (A1)

## Create a subset of the data for FaunalCluster A1a
A1SS= subset(data4best10v2,FaunalCluster=="A1")

## Check number of samples in df 'A1' tallies with the above table
dim(A1SS)# 297 it does

## Generate random numbers (without replacement) from 1 to n
set.seed(1)# to stop random numbers changing
A1SSnum=sample(1:297,297)

## Add random numbers to new col 'A1SS' in df A1SS
A1SS$SSNumber <-A1SSnum

## Now do the same for FaunalCluster group A2a
A2aSS= subset(data4best10v2,FaunalCluster=="A2a")
dim(A2aSS)# 514 it does
set.seed(2)# to stop random numbers changing
A2aSSnum=sample(1:514,514)
A2aSS$SSNumber <-A2aSSnum

## Now do the same for FaunalCluster group A2b
A2bSS= subset(data4best10v2,FaunalCluster=="A2b")
dim(A2bSS)# 813 it does
set.seed(3)# to stop random numbers changing
A2bSSnum=sample(1:813,813)
A2bSS$SSNumber <-A2bSSnum

## Now do the same for FaunalCluster group B1a
B1aSS= subset(data4best10v2,FaunalCluster=="B1a")
dim(B1aSS)# 1058 it does
set.seed(4)# to stop random numbers changing
B1aSSnum=sample(1:1058,1058)
B1aSS$SSNumber <-B1aSSnum

## Now do the same for FaunalCluster group B1b
B1bSS= subset(data4best10v2,FaunalCluster=="B1b")
dim(B1bSS)# 776 it does
set.seed(5)# to stop random numbers changing
B1bSSnum=sample(1:776,776)
B1bSS$SSNumber <-B1bSSnum

## Now do the same for FaunalCluster group C1a
C1aSS= subset(data4best10v2,FaunalCluster=="C1a")
dim(C1aSS)# 1501 it does
set.seed(6)# to stop random numbers changing
C1aSSnum=sample(1:1501,1501)
C1aSS$SSNumber <-C1aSSnum

## Now do the same for FaunalCluster group C1b
C1bSS= subset(data4best10v2,FaunalCluster=="C1b")
dim(C1bSS)# 1180 it does
set.seed(7)# to stop random numbers changing
C1bSSnum=sample(1:1180,1180)
C1bSS$SSNumber <-C1bSSnum

## Now do the same for FaunalCluster group D1
D1SS= subset(data4best10v2,FaunalCluster=="D1")
dim(D1SS)# 319 it does
set.seed(8)# to stop random numbers changing
D1SSnum=sample(1:319,319)
D1SS$SSNumber <-D1SSnum

## Now do the same for FaunalCluster group D2a
D2aSS= subset(data4best10v2,FaunalCluster=="D2a")
dim(D2aSS)# 1832 it does
set.seed(9)# to stop random numbers changing
D2aSSnum=sample(1:1832,1832)
D2aSS$SSNumber <-D2aSSnum

## Now do the same for FaunalCluster group D2b
D2bSS= subset(data4best10v2,FaunalCluster=="D2b")
dim(D2bSS)# 997 it does
```

```
set.seed(10)# to stop random numbers changing
D2bSSnum=sample(1:997,997)
D2bSS$SSNumber <-D2bSSnum

## Now do the same for FaunalCluster group D2c
D2cSS= subset(data4best10v2,FaunalCluster=="D2c")
dim(D2cSS)# 4065 it does
set.seed(11)# to stop random numbers changing
D2cSSnum=sample(1:4065,4065)
D2cSS$SSNumber <-D2cSSnum

## Now do the same for FaunalCluster group D2d
D2dSS= subset(data4best10v2,FaunalCluster=="D2d")
dim(D2dSS)# 1388 it does
set.seed(12)# to stop random numbers changing
D2dSSnum=sample(1:1388,1388)
D2dSS$SSNumber <-D2dSSnum

## Stitch together all the data subsets
best.data.ss=rbind(A1SS,A2aSS,A2bSS,B1aSS,B1bSS,C1aSS,C1bSS,D1SS,D2aSS,D2bSS,D2cSS,D2dSS)

## Check dimensions of df 'data4CCA10'
dim(data4best10v2)# 14740 781

## Check dimensions of df 'best.data.ss' - should be same as df 'data4best10v2', but with
# one extra column
dim(best.data.ss)# 14740 782 -it is

## Now select only samples with random no. 1:297
best.data.ss.final=subset(best.data.ss, SSNumber<298)
dim(best.data.ss.final)# should be 3564 (12 x 297) -it is

## Check cols for faunal data
names(best.data.ss.final)# 2:650
View(best.data.ss.final)
dim(best.data.ss.final)#3564 782

## Select wanted variables: faunal data (2:650), Latitude_WGS84(753),Sal (763),Temp (764),
# Chla (765), SPM (766),Depth (767),WOV (768),AvCur (769),Stress (770),Mud (777),Sand (778),
# Gravel (779),FaunalCluster (781)
best.data.ss.final2=best.data.ss.final[,c(2:650,753,763,764,765,766,767,768,769,770,777,778,
                                           779,781)]
## Check selected columns
names(best.data.ss.final2)

## Now need to check which faunal variables are redundant (i.e. no records). Make a note of
# cols in excel.
write.csv(best.data.ss.final2,file = "OUTPUTS/best.data.ss.final2.csv",row.names=TRUE)

## Remove variables with no abund  (got list from csv file). There will be taxa with no abund
# as some samples with taxa present have been deleted in gear step above#
best.data.ss.final3 <- best.data.ss.final2[-c(1,5,10,12,15,51,57,81,113,115,120,125,127,135,
                                              136,154,162,166,171,175,181,184,185,188,195,
                                              196,199,212,222,233,238,244,252,254,259,269,
                                              277,281,284,288,321,326,341,345,359,368,370,
                                              396,401,410,437,438,445,451,453,471,479,481,
                                              487,489,515,521,535,542,548,553,558,572,573,
                                              581,585,594,605,606,612,614,615,617,618,625,
                                              632)]

## Check some taxa have been removed
dim(best.data.ss.final3)#3564 581 - they have
names(best.data.ss.final3)

## Create a df 'bestBIO' for faunal data and save
bestBIO=best.data.ss.final3[,c(1:568)]
write.csv(bestBIO,file = "OUTPUTS/bestBIO.csv",row.names=TRUE)

## Create a df 'bestPHY' for physical variables and save.Lat (569), Sal (570), Temp (571),
# Chla (572), SPM (573),Depth (574), WOV (575),AvCur (576), Stress (577), Mud (578),
# Sand (579), Gravel (580)
bestPHY=best.data.ss.final3[,c(569:580)]
write.csv(bestPHY,file = "OUTPUTS/bestPHY.csv",row.names=TRUE)

## Create a df 'bestFAC' for factor (FaunalCluster(581)) and save
bestFAC=best.data.ss.final3[,581]
```

```
write.csv(bestFAC,file = "OUTPUTS/bestFAC.csv",row.names=TRUE)

###################################################################################################
### STEP REF:       38                                                                          ###
###                                                                                             ###
### PAPER SECTION:  METHODS \ Faunal Data Analysis \ Explaining patterns in faunal              ###
###                 distribution                                                                ###
###                 RESULTS \ Faunal Data Analysis \ Explaining patterns in faunal              ###
###                 distribution                                                                ###
###                                                                                             ###
### TASK:           Identify important environmental predictor variables using 'best'          ###
###                 function from the vegan package                                             ###
###                                                                                             ###
### NOTES:          Transform biological data                                                   ###
###                 Address collinearity for environmental variables                            ###
###                 Transform data for explanatory variables to address skewness               ###
###                 No need to normaise environmental data as this is done by automatically    ###
###                 in best                                                                     ###
###################################################################################################

## Load vegan package
library("vegan")

## Load faunal and phy variable data
varespec=read.csv("OUTPUTS/bestBIO.csv", header=T,na.strings=c("NA", "-", "?","<null>"),
                  stringsAsFactors=F)
varephy=read.csv("OUTPUTS/bestPHY.csv", header=T,na.strings=c("NA", "-", "?","<null>"),
                 stringsAsFactors=F)
varefac=read.csv("OUTPUTS/bestFAC.csv", header=T,na.strings=c("NA", "-", "?","<null>"),
                 stringsAsFactors=F)

## View objects to check ok
#View(varespec)# need to delete 1st col
#View(varephy)# need to delete 1st col
#View(varefac)# need to delete 1st col

## Remove first col (X) in dfs 'varespec' and 'varephy'and change values to rownames
varespec=varespec[,-1]
varephy=varephy[,-1]

## Change name of col varephy$Latitude_WGS84 to varephy$Lat
colnames(varephy)[1] <- "Lat"

## Create a new df 'varespec2' for 4th root transformed data from df 'varespec'
varespec2=varespec^(0.25)

## Rename columns in df 'varefac'
colnames(varefac)=c("Number","FaunalCluster")

## Change varefac$FaunalCluster from chr to factor
varefac$FaunalCluster=as.factor(varefac$FaunalCluster)

## Detecting collinearity Using Variance Inflation Factors (VIFs) from usdm package
library(usdm)
names(varephy)

## Get correlation coefficients
cor(varephy, use="all.obs", method="spearman")

## Get VIF scores for variables in df 'varphy'and note higest value above 2.5
vif(varephy) #Temp has highest VIF score

## Get VIF scores for variables in df 'varephy'(minus Temp) and note highest value above 2.5
vif(varephy[,c(1,2,4,5,6,7,8,9,10,11,12)])#Temp removed. Gravel highest remaining VIF

## Get VIF scores for variables in df 'varephy'(minus Temp and Gravel) and note highest
# value above 2.5
vif(varephy[,c(1,2,4,5,6,7,8,9,10,11)])#Gravel removed. Chla highest remaining VIF

## Get VIF scores for variables in df 'varephy'(minus Temp, Gravel and Chl a) and note
# highest value above 2.5
vif(varephy[,c(1,2,5,6,7,8,9,10,11)])#Chla removed.Stress highest remaining VIF

## Get VIF scores for variables in df 'varephy'(minus Temp, Gravel, Chl a and Stress)
# and note highest value above 2.5
vif(varephy[,c(1,2,5,6,7,8,10,11)])#Stress removed. Now all with VIF score <2.5
```

```
## Select variables from df varephy to take forward (Lat (1), Sal (2), SPM (5), Depth (6),
# WOV (7), AvCur (8), Mud (10), Sand (11)).
varephy2=varephy[,c(1,2,5,6,7,8,10,11)]

## Check for any skewness in the data. Negative skewness indicates that the mean of the data
# values is less than the median,and the data distribution is left-skewed. Positive skewness
# would indicate that the mean of the data values is larger than the median, and the data
# distribution is right-skewed.
summary(varephy2)# Positive skewness (mean>median) for SPM (3), Depth (4) and Mud (7)

## Transform relevant columns (SPM (3), Depth(4), Mud(7))
varephy2[,c(3,4,7)]=log(varephy2[,c(3,4,7)]+0.1)

## Run bioenv with the transformed faunal (df 'varespec2') and the selected env variables
#(df 'varephy2'). Note env variables will be normaised during best.
res<-bioenv(varespec2, varephy2)
res

## See all best results
summary(res)

###################################################################################################
### STEP REF:       39                                                                         ###
###                                                                                            ###
### PAPER SECTION:  METHODS \ Faunal Data Analysis \ Explaining patterns in faunal             ###
###                 ditribution                                                                ###
###                 RESULTS \ Faunal Data Analysis \ Explaining patterns in faunal             ###
###                 distribution                                                               ###
###                                                                                            ###
### TASK:           Use 'adonis' to quantify the variation in faunal data explained by         ###
###                 the environmental predictors identified in 'best'                          ###
###                                                                                            ###
###################################################################################################

## Normalise env data prior to using adonis (subtract by pop mean and sd) of phy variable data
varephy3 <- scale(varephy2)# scale the transformed data

## varephy3 is a matrix so need to convert back to a df for use with adonis
varephy3=as.data.frame(varephy3)

## Use Adonis to see how much of the variation is explained by the different variables. Enter
# phy variable which are important (see BEST results)
# http://www.talkstats.com/showthread.php/15912-Permanova-Adonis
adonis.res=adonis(formula = varespec2 ~ AvCur + Mud + Sand, data = varephy3, permutations = 999,
                  method = "bray")

adonis.res

###################################################################################################
### STEP REF:       40                                                                         ###
###                                                                                            ###
### PAPER SECTION:  METHODS \ Faunal Data Analysis \ Explaining patterns in faunal             ###
###                 ditribution                                                                ###
###                 RESULTS \ Faunal Data Analysis \ Explaining patterns in faunal             ###
###                 distribution                                                               ###
###                                                                                            ###
### TASK:           Distance based redundancy analysis (dbRDA) ordination to visualise the    ###
###                 the relationship between macrofaunal data and predictor variables          ###
### NOTES:                                                                                     ###
###################################################################################################

##dbRDA https://www.rdocumentation.org/packages/vegan/versions/2.4-2/topics/capscale
vare.cap <- capscale(varespec2 ~ AvCur + Mud + Sand, varephy3,dist="bray")

vare.cap

palette(c("blue2","cyan1","#05aae1","plum2","darkorchid3","green3",
          "palegreen1","#b40202","red1","darkorange","yellow",
          "#b4b404"))

## Save plot to an image file (png or tiff)
png("OUTPUTS/FIGURE 6.png",width = 14,height = 12.5,units = "cm", res = 800, pointsize = 12)
#tiff("OUTPUTS/FIGURE 6.tiff",width = 14,height = 12.5,units = "cm",res = 800,pointsize = 12)

plot(vare.cap,type="none",scaling=2,xlim = c(-2, 2),ylim = c(-3, 3),
```

```
     cex.lab=0.7,cex.axis=0.7)

## Add sites as points coloured by cluster group. Samples shown as points representing their
# mean species composition.
points(vare.cap, col=varefac$FaunalCluster,cex = 0.25,pch=16)

# Add explantory variable vectors pointing in the direction of max increase.  Long vectors
#equals strong trends
text(vare.cap, dis="cn",col="black",cex=0.7)

legend("topleft", pch = 20,pt.cex=1, col=unique(varefac$FaunalCluster),
       legend = unique(varefac$FaunalCluster),cex=.55)

dev.off()

anova(vare.cap)

###############################################################################################
### STEP REF:        41                                                                     ###
###                                                                                         ###
### PAPER SECTION:   METHODS \ Faunal data analysis \ Explaining patterns in faunal         ###
###                  distribution                                                           ###
###                  RESULTS \ Faunal data analysis \ Explaining patterns in faunal         ###
###                  distribution                                                           ###
###                                                                                         ###
### TASK:            Supplementary Figure S1 (Heat maps for explanatory variables)          ###
###                                                                                         ###
###############################################################################################

## Required data in df 'data'
names(data)

## Select relevant cols from df 'data':"X125mm"(776) to "Pan"(874), "Latitude_WGS84" (878),
# "Longitude_WGS84"(879),"Av_current"(894), "Depth"(892),"WOV"(893), "Chla"(890), "SPM"(891),
# "Temp"(889),"Sal"(888),"Stress"(895)
varmapdata=data[,c(874:776,878,879,894,892,893,890,891,889,888,895)]

## Change Depth values to positive
varmapdata$Depth=abs(varmapdata$Depth)

## Change an 'NA' values in sieve data to zero
varmapdata[, 1:99][is.na(varmapdata[, 1:99])] <- 0

## Add in cols for Mud, Sand and Gravel
varmapdata$Mud=rowSums(varmapdata[1:39])
varmapdata$Sand=rowSums(varmapdata[40:66])
varmapdata$Gravel=rowSums(varmapdata[67:99])

#select only variable of interest
varmapdata2=varmapdata[,100:112]

## Change names of col 1 from 'Latitude_WGS84' to 'Lat'
colnames(varmapdata2)[1]="Lat"

## Change names of col 2 from 'Longitude_WGS84' to 'Long'
colnames(varmapdata2)[2]="Long"

## Add a col 'tperc' for the total sediment percent
varmapdata2$tperc=rowSums(varmapdata2[11:13])

## Remove samples where tperc is not equal to ~100
varmapdata2.5=subset(varmapdata2, tperc > 99 & tperc < 101)

## Remove col 'tperc'
varmapdata2.5$tperc=NULL

## Remove rows where there is a value of NA in one of the columns
varmapdata3=varmapdata2.5[complete.cases(varmapdata2.5),]

## Rank values for each variable
Depth=rank(varmapdata3$Depth)
AvCur=rank(varmapdata3$AvCur)
WOV=rank(varmapdata3$WOV)
Chla=rank(varmapdata3$Chla)
SPM=rank(varmapdata3$SPM)
Temp=rank(varmapdata3$Temp)
```

```
Sal=rank(varmapdata3$Sal)
Stress=rank(varmapdata3$Stress)
Mud=rank(varmapdata3$Mud)
Gravel=rank(varmapdata3$Gravel)
Sand=rank(varmapdata3$Sand)
Lat=varmapdata3$Lat
Long=varmapdata3$Long

## Bind variable rank columns together
varmapdata3.5=cbind(Lat,Long,AvCur,Depth,WOV,Chla,SPM,Temp,Sal,Stress,Mud,Sand,Gravel)

## Change class of df varmapdata3.5 from a matrix to a df
varmapdata3.5=as.data.frame(varmapdata3.5)

## Get data into a suitable form for feceting
library(reshape2)
varmapdata4=melt(varmapdata3.5,c("Lat","Long"))

## Call colouRamps for maps
require( colorRamps )

## Produce plot
PhyVarMaps= ggplot()+
  geom_polygon(data=defra.dem.df, aes(x=long, y=lat, group=group,fill=Depth))+
  scale_fill_manual(values=grey,name="Depth (m)",guide = FALSE)+ # Turn off DEM legend
  geom_point(data=varmapdata4,aes(Long,Lat,col=value, size=value))+
  scale_size(range = c(0.04, 0.04),guide = FALSE)+
  labs(colour="Rank")+# to change point legend from 'rank(abundance)' to 'Rank'
  geom_polygon(data=euDF2, aes(x=long, y=lat, group=group),fill="white",colour="black",
               size=0.1)+
  coord_map(xlim = c(-10.7, 4),ylim = c(48, 62)) + #set x,y limits of plot
  theme_bw(base_size = 14)+
  labs(x="Longitude",y="Latitude")+
  facet_wrap(~variable)+
  scale_color_gradientn(colors= matlab.like(50))

## Save plot to an image file (png or tiff) width was 22.5
png("OUTPUTS/FIGURE S1.png", width = 24, height = 28.2, units = "cm",
    res = 800,pointsize = 12)
#tiff("OUTPUTS/FIGURE S1.tiff", width = 24, height = 28.2, units="cm",res=800,pointsize = 12)
PhyVarMaps+ theme(legend.key.size = unit(0.5, "cm"))
dev.off()

################################################################################################
# # #                                  (E) SEDIMENT ANALYSIS                               # # #
################################################################################################

################################################################################################
### STEP REF:        42                                                                     ###
###                                                                                         ###
### PAPER SECTION:  METHODS \ Faunal-sediment relationships \ Identification of physical     ###
###                 cluster groups                                                          ###
###                 RESULTS \ Faunal-sediment relationships \ Identification of physical     ###
###                 cluster groups                                                          ###
###                                                                                         ###
### TASK:           Prepare data for clustering of physical variables                       ###
################################################################################################

## Select relevant variables from df 'data4.5' (subset of raw data based on selection criteria
# for: gear, sieve, faunal presence)
names(data4.5)

## Check dimensions of df 'data4.5'
dim(data4.5)#27432 830

## Create a df 'phy.data' which is the subset of chosen variables (see above) from df 'data4.5
# Variables of interest are: Sample (1), Latitude_WGS84 (807), Longitude_WGS84 (808),Sal (817),
# Temp (818), Chla (819), SPM (820), Depth (821), WOV (822), AvCur (823), Stress (824)
phy.data=data4.5[,c(1,807,808,817,818,819,820,821,822,823,824)]

## Check selected columns present
names(phy.data)

## Change bathy values to positive
phy.data$Depth=abs(phy.data$Depth)
```

```
## Remove rows where there is a value of NA in one of the columns
phy.data.complete=phy.data[complete.cases(phy.data),]

## Remove variables from df phy.data that won't be used for clustering (i.e. Sample,
# Latitude_WGS84, and Longitude_WGS84)
phy.data.final=phy.data.complete[,4:11]

## Check unwanted varaibales removed
names(phy.data.final)

## Check dimensions of df 'phy.data.final
dim(phy.data.final)#26406 8

## Identify any skewness in the data. Intuitively, the skewness is a measure of symmetry. As a
# rule, negative skewness indicates that the mean of the data values is less than the median,
# and the data distribution is left-skewed. Positive skewness would indicates that the mean of
# the data values is larger than the median, and the data distribution is right-skewed.
summary(phy.data.final)# RS (Mean>median) observed for SPM (4), Depth (5), Stress (8)

## Create a copy of df CCA.PHY
phy.data.finalT=phy.data.final

## Transform relevant columns
phy.data.finalT[,c(4,5,8)]=log(phy.data.finalT[,c(4,5,8)]+0.1)

## Normalisation of phy variable data (subtract by pop mean and sd)
df <- scale(phy.data.finalT)# scale the data

## Dimensions of df 'df'
dim(df)#26406 8
#View(df)

###################################################################################################
### STEP:           43                                                                          ###
###                                                                                             ###
### PAPER SECTION:  METHODS \ Faunal-sediment relationships \ Identification of physical        ###
###                 cluster groups                                                              ###
###                 RESULTS \ Faunal-sediment relationships \ Identification of physical        ###
###                 cluster groups                                                              ###
###                                                                                             ###
### TASK:           Figure 3b (Elbow plot to determine number of physical cluster groups)       ###
###                                                                                             ###
### NOTES:          This plot will be combined with others in step 46                           ###
###################################################################################################

## Determine the appropriate number of cluster groups
Phy.wssplot2 <- function(df, nc=50, seed=1234){
  wss <- (nrow(df)-1)*sum(apply(df,2,var))
  for (i in 2:nc){
    set.seed(seed)
    #wss[i] <- sum(kmeans(df, centers=i)$withinss)}
    wss[i] <- sum(kmeans(df, algorithm="MacQueen",iter.max=100,nstart=25,centers=i)$withinss)}
  plot(1:nc, wss, type="b", xlab="Number of Clusters",
       ylab="Within groups sum of squares")}

## Save plot to an image file (png or tiff)
png("OUTPUTS/FIGURE 3b ELBOW PLOT.png",width = 15,height = 13,units = "cm",res = 800,
    pointsize = 12)
#tiff("OUTPUTS/FIGURE 3b ELBOW PLOT.tiff",width = 15,height = 13,units = "cm",res = 800,
#pointsize = 12)

## Set text size
par(cex=0.8)

## Produce plot
Phy.wssplot2(df)

## Add line to show position of elbow
abline(v=10,col="red")

dev.off()

###################################################################################################
### STEP:           44                                                                          ###
###                                                                                             ###
### PAPER SECTION:  METHODS \ Faunal-sediment relationships \ Identification of physical        ###
```

```
###                 cluster groups                                                     ###
###                 RESULTS \ Faunal-sediment relationships \ Identification of physical      ###
###                 cluster groups                                                     ###
###                                                                                    ###
### TASK:           K-means clustering of Physical variables data                      ###
##########################################################################################

## kmeans clutering
set.seed(1234)
phy.results=kmeans(df,10,algorithm="MacQueen",iter.max=100,nstart=25)

## Create a df 'phy.cluster' for the cluster groups
phy.cluster=as.data.frame(phy.results$cluster)

## Create an object 'phy.output' with sample coordinates and cluster group.
phy.output=cbind(phy.data.complete$Sample,phy.data.complete$Latitude_WGS84,
                 phy.data.complete$Longitude_WGS84,phy.cluster)

## Reinstate the appropriate column names
colnames(phy.output) <- c("Sample", "Latitude_WGS84","Longitude_WGS84","PhyCluster")

## Change variable PhyCluster from an integer to a factor
phy.output$PhyCluster=as.factor(phy.output$PhyCluster)

## Check names of df 'phy.output'
names(phy.output)

## Number of samples belonging to each cluster group
phy.results$size# 2472 1732 3818 4299 1344 2108 3093 4788 1750 1002

##########################################################################################
### STEP:           45                                                                 ###
###                                                                                    ###
### PAPER SECTION:  METHODS\ Faunal-sediment relationships\ Identification of physical       ###
###                 cluster groups                                                     ###
###                 RESULTS\ Faunal-sediment relationships\ Identification of physical       ###
###                 cluster groups                                                     ###
###                                                                                    ###
### TASK:           Figure 3b Dendrogram (Relationship between physical cluster groups)       ###
###                                                                                    ###
### NOTES:          This plot will be combined with others in step 46                  ###
##########################################################################################

## Data has been transformed with each variable is on a reasonably equal footing.  Now calculate
# the absolute differences between cluster centres over all variables.

nclusters = 10

absdiff = matrix(0, nrow=nclusters, ncol=nclusters)
centers = phy.results$centers

for (j in 1:nclusters) {
  for (k in 1:nclusters) {
    absdiff[j,k] = sum(abs(centers[j,] - centers[k,]))
  }
}

dp=round(absdiff, 1)

## Find distance matrix
d1 <- dist(as.matrix(dp))

## Apply hirarchical clustering
hc.phy <- hclust(d1)

## Save plot of dendrogram to an image file (png or tiff)
png("OUTPUTS/FIGURE 3b DENDRO.png",width = 15,height = 13,units = "cm",res = 800,
    pointsize = 12)
#tiff("OUTPUTS/FIGURE 3b DENDRO.tiff",width = 15,height = 13,units = "cm",res = 800,
#     pointsize = 12)

## Convert hclust into a dendrogram and plot
hcd.phy <- as.dendrogram(hc.phy)

## reduced label size of cluster labels 1-15. Need to do this outside the plot command
par(cex=0.8)
```

```
## Plot dendrogram
plot(hcd.phy, ylab = "Height")

## Add circle symbols below each leaf of the dendrogram. Define colours.
col.circle=c('#FF62BC','#FFFF32','#39B600','#00BFC4','#8681E5','#A3A500','#e31a1c','#ff7f00',
             '#fdbf6f','#1f78b4')

## Add symbols
symbols(1:10, rep(-4, 10), circles=rep(1, 10), add=TRUE, inches=.08,fg=col.circle,bg=col.circle,
        xpd=TRUE)

dev.off()

###############################################################################################
### STEP:           46                                                                      ###
###                                                                                         ###
### PAPER SECTION:  METHODS \ Faunal data analysis \ Community analysis                     ###
###                 METHODS\ Faunal-sediment relationships\ Identification of physical      ###
###                 cluster groups                                                          ###
###                 RESULTS \ Faunal data analysis \ Community analysis \ Clustering        ###
###                 RESULTS \ Faunal-sediment relationships\ Identification of physical     ###
###                 cluster groups                                                          ###
###                                                                                         ###
### TASK:           Figure 3 (Combined  elbow and cluster plots for fauna and physical data)  ###
###############################################################################################

## Load png package
#install.packages("png")
library("png") # for reading in PNGs

## Bring in png images for elbow plots and dendrograms
faunal.elbow = readPNG("OUTPUTS/FIGURE 3a ELBOW PLOT.png")
faunal.dendro = readPNG("OUTPUTS/FIGURE 3a DENDRO.png")
phy.elbow = readPNG("OUTPUTS/FIGURE 3b ELBOW PLOT.png")
phy.dendro = readPNG("OUTPUTS/FIGURE 3b DENDRO.png")

png("OUTPUTS/FIGURE 3.png", width = 22, height = 19, units = "cm", res = 600,pointsize = 14)
#tiff("OUTPUTS/FIGURE 3.tiff", width = 22, height = 19, units = "cm", res = 600,pointsize = 14)

## Set up screen - no margins
par(mar=rep(0,4))

## Create a layout with 3 rows and 2 columns. 1st row columns combined
layout(matrix(c(1,1,1,2,3,4,5,6,7), 3, 3, byrow = TRUE),widths=c(1,12,12),heights=c(1,12.5,12.5))

## Plot titles
plot.new()# plot for titles
mtext("         Elbow plots                                              Dendrograms",
      side=3, outer=TRUE, line=-2)

## a) label
plot.new()# plot for a)label
text(0.4,0.85,"a)",cex=1.4,font=2)

## Faunal elbow plot
plot(NA,xlim=0:1,ylim=0:1,xaxt="n",yaxt="n",bty="n")
rasterImage(faunal.elbow,0,0,1,1)

## Faunal dendrogram
plot(NA,xlim=0:1,ylim=0:1,xaxt="n",yaxt="n",bty="n")
rasterImage(faunal.dendro,0,0,1,1)

## b) label
plot.new()
text(0.4,0.85,"b)",cex=1.4,font=2)

## Physical elbow plot
plot(NA,xlim=0:1,ylim=0:1,xaxt="n",yaxt="n",bty="n")
rasterImage(phy.elbow,0,0,1,1)

## Physical dendrogram
plot(NA,xlim=0:1,ylim=0:1,xaxt="n",yaxt="n",bty="n")
rasterImage(phy.dendro,0,0,1,1)

dev.off()
```

```
#############################################################################################
### STEP REF:        47                                                                  ###
###                                                                                      ###
### PAPER SECTION:   METHODS \ Faunal-sediment relationships \ Identification of physical      ###
###                  cluster groups                                                      ###
###                  RESULTS \ Faunal-sediment relationships \ Identification of physical      ###
###                  cluster groups                                                      ###
###                                                                                      ###
### TASK:            Figure 8a (Physical cluster group identity for individual stations)      ###
#############################################################################################

## Produce a map of physical cluster group distributuion
PhyClusMap= ggplot()+
  geom_polygon(data=defra.dem.df, aes(x=long, y=lat, group=group,fill=Depth))+
  scale_fill_manual(values=grey,name="Depth (m)",guide=FALSE)+
  geom_polygon(data = euDF2, aes(x=long, y=lat, group = group),fill="white",colour="black",
             size=0.05)+
  geom_point(data=phy.output,aes(Longitude_WGS84,Latitude_WGS84,col=PhyCluster), size=0.45,
           show.legend = TRUE)+
  scale_colour_manual(values=c('#e31a1c','#FF62BC','#fdbf6f','#ff7f00','#FFFF32','#8681E5',
                              '#00BFC4','#A3A500','#1f78b4','#39B600'))+
    geom_polygon(data=licence.df, aes(x=long, y=lat, group=group), colour="black",size=0.15,
             fill=NA)+#Licencsed areas
  geom_polygon(data=appl.df, aes(x=long, y=lat, group=group), colour="black",size=0.15,
             fill=NA)+#Application Areas
  geom_polygon(data=goodwinlic.df, aes(x=long, y=lat, group=group), colour="black", size=0.15,
             fill=NA)+#Goodwin Sands
  geom_polygon(data=humSIZ.df, aes(x=long, y=lat, group=group), colour="black", size=0.15,
             linetype=2,fill=NA)+#Humber SIZs
  geom_polygon(data=angSIZ.df, aes(x=long, y=lat, group=group), colour="black", size=0.15,
             linetype=2,fill=NA)+#Anglian SIZs
  geom_polygon(data=thaSIZ.df, aes(x=long, y=lat, group=group), colour="black", size=0.15,
             linetype=2,fill=NA)+#Thames SIZs
  geom_polygon(data=tha5012SIZ.df, aes(x=long, y=lat, group=group), colour="black", size=0.15,
             linetype=2,fill=NA)+#Thames Area 501/2 SIZ
  geom_polygon(data=scSIZ.df, aes(x=long, y=lat, group=group), colour="black", size=0.15,
             linetype=2,fill=NA)+#S.Coast SIZs
  geom_polygon(data=eecSIZ.df, aes(x=long, y=lat, group=group), colour="black", size=0.15,
             linetype=2,fill=NA)+#EEC SIZs
  geom_polygon(data=bcSIZ.df, aes(x=long, y=lat, group=group), colour="black", size=0.15,
             linetype=2,fill=NA)+#Bristol Channel SIZs
  geom_polygon(data=nw457SIZ.df, aes(x=long, y=lat, group=group), colour="black", size=0.15,
             linetype=2,fill=NA)+#Area 457 North West SIZ
  geom_polygon(data=nw392SIZ.df, aes(x=long, y=lat, group=group), colour="black", size=0.15,
             linetype=2,fill=NA)+#Area 392 North West SIZ
  geom_polygon(data=goodwinSIZ.df, aes(x=long, y=lat, group=group), colour="black", size=0.15,
             linetype=2,fill=NA)+#Goodwin SIZ
  coord_map(xlim = c(-10.7, 4),ylim = c(48, 62)) + #set x,y limits of plot
  theme_bw(base_size=24)+
  guides(colour = guide_legend(override.aes = list(size=3)))+ # Change size of legend dots
  labs(x="Longitude",y="Latitude")#
#facet_wrap(~PhyCluster)# Add this line to facet by PhyCluster

fig8a=PhyClusMap+theme(legend.key.size = unit(1, "cm"))+
  guides(colour = guide_legend(override.aes = list(size=8)))

## Save plot to an image file (png or tiff)
png("OUTPUTS/FIGURE 8a.png", width = 29.7,height = 42,units = "cm",res = 1000,pointsize = 48)
#tiff("OUTPUTS/FIGURE 8a.tiff",width = 29.7,height = 42,units = "cm",res = 600,pointsize = 48)
fig8a
dev.off()

## To id colours used in this plot. Enter number of colours in brackets
library(scales)
scales::hue_pal()(10)

#############################################################################################
### STEP REF:        48                                                                  ###
###                                                                                      ###
### PAPER SECTION:   METHODS \ Faunal-sediment relationships \ Identification of physical      ###
###                  cluster groups                                                      ###
###                  RESULTS \ Faunal-sediment relationships \ Identification of physical      ###
###                  cluster groups                                                      ###
###                                                                                      ###
### TASK:            Figure 8b: Physical variable box plots by physical cluster group         ###
```

```
###                                                                                          ###
################################################################################################

## Create a df with required data: Sal,Temp,Chl a, SPM, Depth, WOV, AvCur, Stress
# (from df 'phy.data.final') and Sample, Latitude_WGS84, Longitude_WGS84 and PhyCluster
# (phy cluster group) (from df 'phy.output).
bp=cbind(phy.data.final,phy.output)

## Create a df 'bp2' with only the phy variables and phy cluster group
bp2=bp[,c(1:8,12)]

## Check required variable present
names(bp2)

## Call library 'reshape2' - required for melting data
library(reshape2)

## Melt data into suitable form for facetting
dat <- melt(bp2,"PhyCluster") # Produces a df of 3 cols (PhyCluster, variable, value)

## Plotting
phyboxplots=ggplot(dat, aes(PhyCluster,value,fill=PhyCluster)) +
  scale_fill_manual(values=c('#e31a1c','#FF62BC','#fdbf6f','#ff7f00','#FFFF32','#8681E5',
                             '#00BFC4','#A3A500','#1f78b4','#39B600'))+
  geom_boxplot(outlier.shape=NA) + # don't display outliers
  theme_bw(base_size = 24)+
  facet_wrap(~variable,scales="free_y")+# was"free"
  labs(y = "Value")

fig8b=phyboxplots+theme(legend.position="none")

## Save plot to an image file (png or tiff)
png("OUTPUTS/FIGURE 8b.png", width = 1000, height = 933,units = "px", pointsize = 20)
#tiff("OUTPUTS/FIGURE 8b.tiff", width = 1000, height = 933,units = "px", pointsize = 20)
fig8b
dev.off()

################################################################################################
### STEP REF:       49                                                                       ###
###                                                                                          ###
### PAPER SECTION:  METHODS \ Faunal-sediment relationships \ Identification of physical     ###
###                 cluster groups                                                           ###
###                 RESULTS \ Faunal-sediment relationships \ Identification of physical     ###
###                 cluster groups                                                           ###
###                                                                                          ###
### TASK:           Figure 8 a and b combined                                                ###
###                                                                                          ###
### NOTES:          Use this code if a plot combining parts a and b is required              ###
################################################################################################

require(cowplot)

## Save plot to an image file (png or tiff).
png("OUTPUTS/FIGURE 8.png",width=70.37, height=42, units="cm", res=1000)
#tiff("OUTPUTS/FIGURE 8.tiff",width=70.37, height=42, units="cm", res=1000)
plot_grid(fig8a,fig8b, labels = c("a)","b)"),nrow = 1,label_size = 24,hjust=0.04)
dev.off()

################################################################################################
### STEP REF:       50                                                                       ###
###                                                                                          ###
### PAPER SECTION:  METHODS \ Faunal-Sediment relationships \ Sediment composition by faunal ###
###                 and faunal-physical cluster groups                                       ###
###                 RESULTS \ Faunal-Sediment relationships \ Sediment composition by faunal ###
###                 and faunal-physical cluster groups                                       ###
###                                                                                          ###
### TASK:           Prepare data for analysis of sediments                                   ###
###                                                                                          ###
### NOTES:          Append Physical cluster results to df of raw data                        ###
###                 Append Faunal cluster results to df of raw data                          ###
###                 Add and populate a column for total sieve percent                        ###
###                 Create a Phy/Faunal cluster group through concatenation of both variables ###
################################################################################################

## Make a copy of the raw data file
data.new=data
```

```
## View col numbers
names(data.new)

## Add new col to df 'data.new' for PhyCluster
data.new["PhyCluster"]=NA

## Check new col added
names(data.new) # it is

## Populate col Phy_Cluster in df 'data.new' by matching the col 'Sample' in df's data.new and
# PhyClusRes.  Note the number at the end of the code below is the col ref for PhyCluster in df
# 'phy.output'
data.new$PhyCluster<-phy.output[match(data.new$Sample, phy.output$Sample),4]

## Check col 'Phy_Cluster' has been filled
data.new[,c(1,902)]

## Add new col to df 'data.new' for FaunalCluster
data.new["FaunalCluster"]=NA

## Check new col added
names(data.new) # it is

## Populate col 'FaunalCluster' in df 'data.new' by matching the Sample code in df's 'data.new'
# and 'faunal.cluster'. Note that the number at the end of the string is the col ref
# for FaunalCluster in df 'faunal.cluster'
data.new$FaunalCluster<-faunal.cluster[match(data.new$Sample, faunal.cluster$Sample),5]

# Check FaunalCluster col populated in df 'data.new'
data.new[,c(1,902:903)] #it is

## Export raw data with Physical and Faunal Cluster groups.
#write.csv(data.new,file = "OUTPUTS/datanew.csv",row.names=FALSE)

## Add col to df 'data.new' for total percent
data.new["TotalPercent"]=NA

##Check new column added
names(data.new)# it is

## Change NAs in sieve data cols to zero so you can sum
data.new[, 776:874][is.na(data.new[, 776:874])] <- 0

## Populate col TotalPercent by summing across the sieve cols: 125mm (776) to Pan (874)
data.new$TotalPercent=rowSums(data.new[,776:874])

#write.csv(data.new,file = "OUTPUTS/datanew.csv",row.names=FALSE)

## Now concatonate cols:  'PhyCluster'and 'FaunalCluster'
data.new$PhyBioCluster <- do.call(paste, c(data.new[c("PhyCluster", "FaunalCluster")],
                                           sep = "_"))

##Check new variable 'PhyBioCluster' added
names(data.new)#it is

## Check new variable
data.new$PhyBioCluster

## Select only samples with a value for FaunalCluster (in effect subset by gear and sieve)
data.new3=subset(data.new, !is.na(FaunalCluster))
dim(data.new3) #27432 905
#names(data.new3)

## subset data for total percent is ~100
sed.data.all <- subset(data.new3, TotalPercent > 99 & TotalPercent < 101)
dim(sed.data.all) #23487 905

## subset data for WenthorthSuitability
sed.data.all1 <- subset(sed.data.all, WentworthSuitability=="Y")
dim(sed.data.all1) #21468 905

## Subset by Treatment
sed.data.all2= subset(sed.data.all1,Treatment=="NA"|Treatment=="R")
dim(sed.data.all2) #19068 905
```

```
## Select data only where top sieve empty
sed.data.all2.5=subset(sed.data.all2, TopSieveEmpty=="Yes"|TopSieveEmpty=="NA")
dim(sed.data.all2.5)# 17773 905

## Select data only where PSA taken as a subsample from the faunal grab
sed.data.all2.6=subset(sed.data.all2.5,PSASubSample=="Yes")
dim(sed.data.all2.6)# 16558 905

## Select data only were samples include colonial taxa
sed.data.all2.7=subset(sed.data.all2.6, IncCol=="Yes")
dim(sed.data.all2.7)# 14145 905

## Select data only were samples collected using a 0.1m2 Hamon grab
sed.data.all2.8=subset(sed.data.all2.7, Gear=="MHN")
dim(sed.data.all2.8)# 12426 905

## Select only columns of interest (sieve data, cluster groups (Phy, Faunal, PhyBio).
sed.data.all3=sed.data.all2.8[,c(1,776:874,902,903,905)]
names(sed.data.all3)

## Check class type is correct for cols (should be chr for col 'Sample' Factors for cluster cols
# and num for sieve cols)
str(sed.data.all3, list.len=ncol(sed.data.all3))

## Change class of FaunalCluster and PhyBioCluster from 'chr' to 'Factor'
sed.data.all3$FaunalCluster=as.factor(sed.data.all3$FaunalCluster)
sed.data.all3$PhyBioCluster=as.factor(sed.data.all3$PhyBioCluster)

## Check class has been changed for cols: FaunalCluster and PhyBioCluster
str(sed.data.all3, list.len=ncol(sed.data.all3))# they have

## Write file for sed data and cluster groups
write.csv(sed.data.all3, file="OUTPUTS/sed.data.all3.csv")

################################################################################################
### STEP REF:       51                                                                       ###
###                                                                                          ###
### PAPER SECTION:  METHODS \ Faunal-Sediment relationships \ Sediment composition by faunal ###
###                 and faunal-physical cluster groups                                       ###
###                 RESULTS \ Faunal-Sediment relationships \ Sediment composition by faunal ###
###                 and faunal-physical cluster groups                                       ###
###                                                                                          ###
### TASK:           Import preprepared data for sediment analysis                            ###
###                                                                                          ###
### NOTES           Use this code if sediment analysis data previously prepared              ###
###                 (i.e. files 'sed.data.all3.csv' is available)                            ###
################################################################################################

## Use this code to avoid having to run code above
sed.data.all3test=read.csv("OUTPUTS/sed.data.all3.csv", header=T,
                          na.strings=c("NA", "-", "?","<null>"),stringsAsFactors=F)

## Remove first col (X)
sed.data.all3test2 <- sed.data.all3test[,-1]

## Change values from col 1 to rownames
rownames(sed.data.all3test2) <- sed.data.all3test[,1]
#View(sed.data.all3test2)

## Reinstate the correct name
sed.data.all3=sed.data.all3test2

################################################################################################
### STEP REF:       52                                                                       ###
###                                                                                          ###
### PAPER SECTION:  METHODS \ Faunal-Sediment relationships \ Sediment composition by faunal ###
###                 and faunal-physical cluster groups                                       ###
###                 RESULTS \ Faunal-Sediment relationships \ Sediment composition by faunal ###
###                 and faunal-physical cluster groups                                       ###
###                                                                                          ###
### TASK:           Prepare sediment data for analysis                                       ###
###                                                                                          ###
### NOTES           Generate sediment variables across a standard set of sieves              ###
################################################################################################

## Start with df of sediment data (sieve and summary measures) and cluster results (Phy, Faunal
```

```
# and PhyBio)
names(sed.data.all3) # id cols to select

## Sieve data and factors only
sed.data.raw=sed.data.all3[,c(100:2,101:103)]
names(sed.data.raw) #check data

## Introduce new cols for sieves of interest
sed.data.raw$CPan=rowSums(sed.data.raw[,1:39])#Pan to 0.045
sed.data.raw$C0.063=rowSums(sed.data.raw[,40:44])#0.0625 to 0.09
sed.data.raw$C0.125=rowSums(sed.data.raw[,45:50])#0.125 to 0.212
sed.data.raw$C0.25=rowSums(sed.data.raw[,51:57])#0.25 to 0.43
sed.data.raw$C0.5=rowSums(sed.data.raw[,58:61])#0.5 to 0.85
sed.data.raw$C1=rowSums(sed.data.raw[,62:66])#1 to 1.7
sed.data.raw$C2=rowSums(sed.data.raw[,67:70])#2 to 3.35
sed.data.raw$C4=rowSums(sed.data.raw[,71:75])#4 to 6.3
sed.data.raw$C8=rowSums(sed.data.raw[,76:80])#8 to 14
sed.data.raw$C16=rowSums(sed.data.raw[,81:88])#16 to 31.5
sed.data.raw$C32=rowSums(sed.data.raw[,89:93])#32 to 63
sed.data.raw$C64=rowSums(sed.data.raw[,94:99])#64 to 1.7

## Check new cols added for sieves of interest
names(sed.data.raw)

## Create a new df 'sed.data.subset' for the sieves of interest + factors only
sed.data.subset=sed.data.raw[,c(103:108,109,110,111:114,100:102)]
names(sed.data.subset) # Check cols

###################################################################################################
### STEP REF:       53                                                                          ###
###                                                                                             ###
### PAPER SECTION:  METHODS \ Faunal-Sediment relationships \ Sediment composition by faunal   ###
###                 and faunal-physical cluster groups                                          ###
###                 RESULTS \ Faunal-Sediment relationships \ Sediment composition by faunal   ###
###                 and faunal-physical cluster groups                                          ###
###                                                                                             ###
### TASK:           Prepare data for calculation of Index of Multivariate Dispersion MVDISP    ###
###                 using Primer 6 software (Values for Tables 3 and S2)                       ###
###################################################################################################

## Select data
sed.var=sed.data.subset[,1:12]

##make row names a column, otherwise Primer interprets first col as sample names
sed.var$Name<-rownames(sed.var)

## Reorder so col Names is the 1st column
sed.var=sed.var[,c(13,1:12)]
#View(sed.var)

## Factor
groups=sed.data.subset$FaunalCluster
PhyBio=sed.data.subset$PhyBioCluster

## Output files for use with Primer
write.csv(sed.var,file = "OUTPUTS/SED VAR FOR IMD CAL.csv",row.names=FALSE)
write.csv(groups,file = "OUTPUTS/BIO CLUSTER.csv",row.names=FALSE)
write.csv(PhyBio,file = "OUTPUTS/PHY BIO CLUSTER.csv",row.names=FALSE)

###################################################################################################
### STEP REF:       54                                                                          ###
###                                                                                             ###
### PAPER SECTION:  METHODS \ Faunal-Sediment relationships \ Sediment composition by faunal   ###
###                 and faunal-physical cluster groups                                          ###
###                 RESULTS \ Faunal-Sediment relationships \ Sediment composition by faunal   ###
###                 and faunal-physical cluster groups                                          ###
###                                                                                             ###
### TASK:           Undertake MVDISP analysis using the Primer 6 software (i.e. not in R)      ###
###                                                                                             ###
### NOTES:          PRIMER>File>Open>All Files>SED VAR FOR IMD CAL.csv>Open                    ###
###                 Data Type>Sample data>Next                                                  ###
###                 Title: Uncheck.Shape: Sample as rows. Data Type: Environment. Blank:       ###
###                       Missing value>Next Text delimiters: Check tab, space, comma>Finish   ###
###                 Add in factors (BioCluster & PhyBioCluster) from files BIO CLUSTER.csv     ###
###                 and PHY BIO CLUSTER.csv. Right mouse click on data>Factors>Add factor      ###
###                 'BIO'and 'PHYBIO'. Open files  BIO CLUSTER.csv and PHY BIO CLUSTER.csv     ###
```

```
###                and paste data into factor column.                                  ###
###                Select samples for taking forward. Note that there must be at least 2   ###
###                samples within each group.  Therefore need to exclude the following     ###
###                levels (PhyBio): 10_A2a, 4_A2a, 4_D1, 6_D1, 7_B1a, 8_B1b, 9_B1a, 9_B1b  ###
###                Select> Samples>factor levels>                                      ###
###                factor name>PhyBio>Levels>remove unwanted samples -see n in paper   ###
###                Analyse>Resemblance>Analyse between:Samples>Measure:Euclidean distance   ###
###                >OK                                                                 ###
###                Analyse>MVDISP>Factor name: PHYBIO (repeat for BIO)                 ###
################################################################################################

################################################################################################
### STEP REF:      55                                                                  ###
###                                                                                    ###
### PAPER SECTION:  METHODS \ Faunal-Sediment relationships \ Sediment composition by faunal  ###
###                and faunal-physical cluster groups                                  ###
###                RESULTS \ Faunal-Sediment relationships \ Sediment composition by faunal  ###
###                and faunal-physical cluster groups                                  ###
###                                                                                    ###
### TASK:          Figure 9a (Sediment composition by Faunal cluster group)            ###
################################################################################################

## Produce a df for mean sediment composition for each FaunalCluster group
sed.data.bio.means=aggregate(sed.data.subset[, 1:12], list(sed.data.subset$FaunalCluster), mean)

## Change format of data ready for plotting
library(reshape2)
sed.data.bio.means2 <- melt(sed.data.bio.means,"Group.1")

## Create a new factor 'Sieve2'which is a copy of col 'variable' (CPan, C0.063 etc)
sed.data.bio.means2$Sieve2=sed.data.bio.means2$variable

## Change col 'Sieve2' to a character as, in current form (factor) it is not possible to change
# to numbers
sed.data.bio.means2$Sieve2=as.character(sed.data.bio.means2$Sieve2)

## Update Sieve2 values from characters to sequential number
sed.data.bio.means2$Sieve2[sed.data.bio.means2$Sieve2=="CPan"]=1
sed.data.bio.means2$Sieve2[sed.data.bio.means2$Sieve2=="C0.063"]=2
sed.data.bio.means2$Sieve2[sed.data.bio.means2$Sieve2=="C0.125"]=3
sed.data.bio.means2$Sieve2[sed.data.bio.means2$Sieve2=="C0.25"]=4
sed.data.bio.means2$Sieve2[sed.data.bio.means2$Sieve2=="C0.5"]=5
sed.data.bio.means2$Sieve2[sed.data.bio.means2$Sieve2=="C1"]=6
sed.data.bio.means2$Sieve2[sed.data.bio.means2$Sieve2=="C2"]=7
sed.data.bio.means2$Sieve2[sed.data.bio.means2$Sieve2=="C4"]=8
sed.data.bio.means2$Sieve2[sed.data.bio.means2$Sieve2=="C8"]=9
sed.data.bio.means2$Sieve2[sed.data.bio.means2$Sieve2=="C16"]=10
sed.data.bio.means2$Sieve2[sed.data.bio.means2$Sieve2=="C32"]=11
sed.data.bio.means2$Sieve2[sed.data.bio.means2$Sieve2=="C64"]=12

## Change name of columns
colnames(sed.data.bio.means2)= c("FaunalCluster","Sieve1","value","Sieve2")

## Change class of col 'Sieve2' from character to numeric
sed.data.bio.means2$Sieve2=as.numeric(sed.data.bio.means2$Sieve2)

## Add a col 'cumvalue' which is the cumulative percentage for each FaunalCluster group
require(plyr)
sed.data.bio.means3 <- ddply(sed.data.bio.means2, .(FaunalCluster), transform,
                             cumvalue = cumsum(value))

## Create bespoke colour scale for use with ggplot
myColors=c("blue2","cyan1","#05aae1","plum2","darkorchid3","green3",
           "palegreen1","#b40202","red1","darkorange","yellow","#b4b404")

names(myColors) <- levels(sed.data.bio.means3$FaunalCluster)
colScale <- scale_colour_manual(name = "FaunalCluster",values = myColors)

## Adding group=1 inside the aes() of geom_line() will ensure that data are connected. If you
# need also a legend, then one way is to put fill= and linetype= inside the aes() with name you
# want to show.
hisp=ggplot(data=sed.data.bio.means3, aes(x=Sieve1,y=cumvalue)) +
  geom_bar(aes(y=value,fill=FaunalCluster),color="black",width=.7, stat="identity") +
  scale_fill_manual(values=c("blue2","cyan1","#05aae1","plum2","darkorchid3","green3",
                             "palegreen1","#b40202","red1","darkorange","yellow","#b4b404"))+
  geom_line(size=1,aes(y=cumvalue,group=1,linetype="Cumulative %",color=FaunalCluster))+
```

```
  geom_point(size=-1,aes(y=cumvalue))+
  facet_wrap(~FaunalCluster,nrow=4)+
  theme_bw()+
  theme(axis.text.x = element_text(angle = 90, hjust = 1, vjust=0.5,size=9)) +
  scale_x_discrete("Sieve (mm)", labels = c("CPan" = "Pan","C0.063" = "0.063",
                                          "C0.125" = "0.125","C0.25" = "0.25",
                                          "C0.5" = "0.5", "C1"="1","C2"="2",
                                          "C4"="4","C8"="8","C16"="16",
                                          "C32"="32","C64"="64"))+
  theme(plot.margin=unit(c(0,0.5,0,1),"cm"))+#add some white space around the plot (T,R,B,L)
  ylab("Cumulative %")

fig9a=hisp+colScale+theme(legend.position="none")

## Save plot to a .png file
png("OUTPUTS/FIGURE 9a.png",width =17 ,height = 21,units = "cm",res = 600,pointsize = 12)
#tiff("OUTPUTS/FIGURE 9a.tiff",width =17,height = 21,units = "cm",res = 600,pointsize = 12)
fig9a
dev.off()

#################################################################################################
### STEP REF:       56                                                                        ###
###                                                                                           ###
### PAPER SECTION:  METHODS \ Faunal-Sediment relationships \ Sediment composition by faunal  ###
###                 and faunal-physical cluster groups                                        ###
###                 RESULTS \ Faunal-Sediment relationships \ Sediment composition by faunal  ###
###                 and faunal-physical cluster groups                                        ###
###                                                                                           ###
### TASK:           Table 3 (Mean sediment composition by faunal cluster group)               ###
###                                                                                           ###
### NOTES:          Table to accompany cum dist plots based on faunal cluster group           ###
#################################################################################################

## Start with DF 'sed.data.subset' containing sieves of interest + factors only
names(sed.data.subset)

## Create a copy of DF sed.data.subset' called'sed.sum.by.fclus'
sed.sum.by.fclus=sed.data.subset

## Introduce new cols for summary sediment varaibles
sed.sum.by.fclus$SC=sed.sum.by.fclus[,1]#Silt/Clay=CPan
sed.sum.by.fclus$fS=rowSums(sed.sum.by.fclus[,2:3])#Fine Sand=C0.063 + C0.125
sed.sum.by.fclus$mS=sed.sum.by.fclus[,4]#mS=CC0.25
sed.sum.by.fclus$cS=rowSums(sed.sum.by.fclus[,5:6])#cS=C0.5+C1
sed.sum.by.fclus$fG=rowSums(sed.sum.by.fclus[,7:8])#fG=C2+C4
sed.sum.by.fclus$mG=sed.sum.by.fclus[,9]#mG=C8
sed.sum.by.fclus$cG=rowSums(sed.sum.by.fclus[,10:12])#cG=C16+C32+C64
sed.sum.by.fclus$TotalSiltClay=sed.sum.by.fclus[,1]#
sed.sum.by.fclus$TotalSand=rowSums(sed.sum.by.fclus[,2:6])#
sed.sum.by.fclus$TotalGravel=rowSums(sed.sum.by.fclus[,7:12])#

## Check new summary sediment cols added
#View(sed.sum.by.fclus)

## Save data - required for step 59 (Mahalanobis)
write.csv(sed.sum.by.fclus, file="OUTPUTS/SEDSUMBYFCLUS.csv",row.names=FALSE)

## (TABLE 3) Identify the number of samples belonging to each FaunalCluster grp
table(sed.sum.by.fclus$FaunalCluster)

## Produce a df for mean sediment composition (summary sed fractions only) for each
# FaunalCluster group
sed.sum.by.fclus.means=aggregate(sed.sum.by.fclus[, c(16:25)],
                                 list(sed.sum.by.fclus$FaunalCluster), mean)

## Change name of col1 to FaunalCluster
colnames(sed.sum.by.fclus.means)[1] <- "FaunalCluster"
#View(sed.sum.by.fclus.means) # Check DF looks ok

## Copy  the data - sed summary means by faunalCluster grp - to a csv, allowing data
# to be put into a table in the report
write.csv(sed.sum.by.fclus.means, file="OUTPUTS/TABLE 3.csv",row.names=FALSE)

## Get sediment descriptions from 'Particle size distribution classification (Blott and Pye,
# 2011).xls' in C5922/WORKSPACE/PAPER
```

```
#################################################################################################
### STEP REF:        57                                                                       ###
###                                                                                           ###
### PAPER SECTION:  METHODS \ Faunal-Sediment relationships \ Sediment composition by faunal  ###
###                 and faunal-physical cluster groups                                        ###
###                 RESULTS \ Faunal-Sediment relationships \ Sediment composition by faunal  ###
###                 and faunal-physical cluster groups                                        ###
###                                                                                           ###
### TASK:           Figure 9b (Sediment composition by faunal-physical cluster group)         ###
#################################################################################################

## Start with df 'sed.data.subset' containing sieves of interest + factors only
names(sed.data.subset)

## Identify the number of samples belonging to each faunal-physical cluster group
PhyBioCount=table(sed.data.subset$PhyBioCluster)

## Output df 'PhyBioCount' as a .csv file (Table 6 n)
write.csv(PhyBioCount, file="OUTPUTS/TABLE S2 n FOR PHYBIO.csv",row.names=FALSE)

## Produce a df for mean sediment composition for each PhyBioCluster group
sed.data.phybio.means=aggregate(sed.data.subset[, 1:12], list(sed.data.subset$PhyBioCluster),
                                mean)

## View df 'sed.data.phybio.means'
#View(sed.data.phybio.means)

## Check dimensions of df 'sed.data.phybio.means'
dim(sed.data.phybio.means)

## Delete rows where no Phy cluster grp (ie where NA present in col Group.1(PhyBioCluster))
sed.data.phybio.means <- sed.data.phybio.means[-grep("\\NA",sed.data.phybio.means$Group.1),]

## Change format of data ready for plotting
library(reshape2)
sed.data.phybio.means2 <- melt(sed.data.phybio.means,"Group.1")

## Create a new factor 'Sieve2'which is a copy of col 'variable' (CPan, C0.063 etc)
sed.data.phybio.means2$Sieve2=sed.data.phybio.means2$variable

## Change col 'Sieve2' to a character as, in current form (factor) it is not possible to
# change to numbers
sed.data.phybio.means2$Sieve2=as.character(sed.data.phybio.means2$Sieve2)

## Update Sieve2 values from characters to sequential number
sed.data.phybio.means2$Sieve2[sed.data.phybio.means2$Sieve2=="CPan"]=1
sed.data.phybio.means2$Sieve2[sed.data.phybio.means2$Sieve2=="C0.063"]=2
sed.data.phybio.means2$Sieve2[sed.data.phybio.means2$Sieve2=="C0.125"]=3
sed.data.phybio.means2$Sieve2[sed.data.phybio.means2$Sieve2=="C0.25"]=4
sed.data.phybio.means2$Sieve2[sed.data.phybio.means2$Sieve2=="C0.5"]=5
sed.data.phybio.means2$Sieve2[sed.data.phybio.means2$Sieve2=="C1"]=6
sed.data.phybio.means2$Sieve2[sed.data.phybio.means2$Sieve2=="C2"]=7
sed.data.phybio.means2$Sieve2[sed.data.phybio.means2$Sieve2=="C4"]=8
sed.data.phybio.means2$Sieve2[sed.data.phybio.means2$Sieve2=="C8"]=9
sed.data.phybio.means2$Sieve2[sed.data.phybio.means2$Sieve2=="C16"]=10
sed.data.phybio.means2$Sieve2[sed.data.phybio.means2$Sieve2=="C32"]=11
sed.data.phybio.means2$Sieve2[sed.data.phybio.means2$Sieve2=="C64"]=12

## Change name of columns
colnames(sed.data.phybio.means2)= c("PhyBioCluster","Sieve1","value","Sieve2")

## Change class of col 'Sieve2' from character to numeric
sed.data.phybio.means2$Sieve2=as.numeric(sed.data.phybio.means2$Sieve2)

## Add a col 'cumvalue' which is the cumulative percentage for each PhyBioCluster grp
require(plyr)
sed.data.phybio.means3 <- ddply(sed.data.phybio.means2, .(PhyBioCluster), transform,
                                cumvalue = cumsum(value))

## View df 'sed.data.phybio.means3'
#View(sed.data.phybio.means3)

## There are 107 PhyBioCluster grps, so you need 105 colours
## Change PhyBioCluster to a factor
sed.data.phybio.means3$PhyBioCluster=as.factor(sed.data.phybio.means3$PhyBioCluster)
```

```
## View all PhyBio Cluster groups
levels(sed.data.phybio.means3$PhyBioCluster)# 105 in total

## Save PhyBio Cluster groups to a .csv file.  In Excel generate PhyCluster and FaunalCluster
# variables from the col PhyBioCluster (use =left, =right et). Use Table\Remove Duplicates
write.csv(sed.data.phybio.means3, file="OUTPUTS/MEANS3.csv",row.names=FALSE)

## Add new cols to sed.data.phybio.means3 for factors 'PhyCluster' and 'FaunalCluster'.

## Create a vector 'values' for the PhyCluster data
values=c("1","1","1","1","1","1","1","1","1","1","1","10","10","10","10","10","10",
         "10","10","10","10","2","2","2","2","2","2","2","3","3","3","3","3","3",
         "3","3","3","3","3","3","4","4","4","4","4","4","4","4","4","4","4","4",
         "5","5","5","5","5","5","5","5","5","6","6","6","6","6","6","6","6","6",
         "7","7","7","7","7","7","7","7","7","7","7","8","8","8","8","8","8","8",
         "8","8","8","8","8","9","9","9","9","9","9","9","9","9","9","9","9")

## Add col PhyCluster to df 'sed.data.phybio.means3' and populate with data from values
# (see above)
sed.data.phybio.means3$PhyCluster=values[sed.data.phybio.means3$PhyBioCluster]

## Create a vector 'values2' for the FaunalCluster data
values2=c("A1","A2b","B1a","B1b","C1a","C1b","D1","D2a","D2b","D2c","D2d","A2a",
          "A2b","B1a","C1a","C1b","D1","D2a","D2b","D2c","D2d","A2a","C1a","C1b",
          "D2a","D2b","D2c","D2d","A1","A2a","A2b","B1a","B1b","C1a","C1b","D1",
          "D2a","D2b","D2c","D2d","A1","A2a","A2b","B1a","B1b","C1a","C1b","D1",
          "D2a","D2b","D2c","D2d","A2a","A2b","C1a","C1b","D1","D2a","D2b","D2c",
          "D2d","A1","A2a","A2b","C1a","C1b","D1","D2a","D2b","D2c","A1","A2a",
          "A2b","B1a","C1a","C1b","D1","D2a","D2b","D2c","D2d","A1","A2a","A2b",
          "B1a","B1b","C1a","C1b","D1","D2a","D2b","D2c","D2d","A1","A2a","A2b",
          "B1a","B1b","C1a","C1b","D1","D2a","D2b","D2c","D2d")

## Add col FaunalCluster to df 'sed.data.phybio.means3' and populate with data from values2
# (see above)
sed.data.phybio.means3$FaunalCluster=values2[sed.data.phybio.means3$PhyBioCluster]

## Remove unused levels for sed.data.phybio.means3$PhyBioCluster (NA_....)
sed.data.phybio.means3$PhyBioCluster <- factor(sed.data.phybio.means3$PhyBioCluster)

## Check data types for df 'sed.data.phybio.means3'
str(sed.data.phybio.means3)

## Change PhyCluster and FaunalCluster from chr to factor
sed.data.phybio.means3$PhyCluster=as.factor(sed.data.phybio.means3$PhyCluster)
sed.data.phybio.means3$FaunalCluster=as.factor(sed.data.phybio.means3$FaunalCluster)

## Check levels for PhyCluster
levels(sed.data.phybio.means3$PhyCluster)

## Order PhyCluster levels in correct numeric order
sed.data.phybio.means3$PhyCluster <- factor(sed.data.phybio.means3$PhyCluster,
                                            levels = c("1","2","3","4","5","6","7","8","9",
                                                       "10"))

## Copy above list to Excel, remove fauna grp string (ie leave only phy cluster info 1_, 2_..)
# and replace with relevant colour - see phy simper
myColors2=c('#e31a1c','#FF62BC','#fdbf6f','#ff7f00','#FFFF32','#8681E5','#00BFC4','#A3A500',
            '#1f78b4','#39B600')

names(myColors2) <- levels(sed.data.phybio.means3$PhyCluster)
colScale2 <- scale_colour_manual(name = "PhyCluster",values = myColors2)

## Facet by FaunalCluster
p=ggplot(sed.data.phybio.means3,aes(Sieve1,cumvalue))+
  geom_point(aes(color=PhyCluster), size=-1)+
  geom_line(size=0.7,aes(Sieve2,cumvalue,color=PhyCluster))+
  theme_bw()+
  labs(x="Sieve (mm)",y="Cumulative %")+
  scale_x_discrete("Sieve (mm)", labels = c("CPan" = "Pan","C0.063" = "0.063",
                                            "C0.125" = "0.125","C0.25" = "0.25",
                                            "C0.5" = "0.5", "C1"="1","C2"="2",
                                            "C4"="4","C8"="8","C16"="16",
                                            "C32"="32","C64"="64"))+
  theme(plot.margin=unit(c(0,0,0,1),"cm"))+#add some white space around the plot (T,R,B,L)
  facet_wrap(~FaunalCluster,nrow=4)#,nrow=4
```

```
## plot with x axis text rotated
fig9b=p+colScale2+ theme(axis.text.x = element_text(angle = 90, hjust=1,vjust=0.5))

## Save plot to a .png file
png("OUTPUTS/FIGURE 9b.png",width =20 ,height = 21,units = "cm",res = 600,pointsize = 12)
#tiff("OUTPUTS/FIGURE 9b.tiff",width =20 ,height = 21,units = "cm",res = 600,pointsize = 12)
fig9b
dev.off()

#################################################################################################
### STEP REF:       58                                                                        ###
###                                                                                           ###
### PAPER SECTION:  METHODS \ Faunal-Sediment relationships \ Sediment composition by faunal  ###
###                 and faunal-physical cluster groups                                        ###
###                 RESULTS \ Faunal-Sediment relationships \ Sediment composition by faunal  ###
###                 and faunal-physical cluster groups                                        ###
###                                                                                           ###
### TASK:           Supplementary Table S2 (Mean sediment composition by faunal-physical      ###
###                 cluster group                                                             ###
###                                                                                           ###
### NOTES:          Data to accompany cumulative distribution plots based on faunal-physical  ###
###                 group                                                                     ###
#################################################################################################

## Start with df 'sed.data.subset' containing sieves of interest + factors only
names(sed.data.subset)

## Creat a df 'sed.sum.by.phybioclus' to manipulate
sed.sum.by.phybioclus=sed.data.subset

## Introduce new cols for summary sediment categories
sed.sum.by.phybioclus$SC=sed.sum.by.phybioclus[,1]#Silt/Clay=CPan
sed.sum.by.phybioclus$fS=rowSums(sed.sum.by.phybioclus[,2:3])#Fine Sand=C0.063 + C0.125
sed.sum.by.phybioclus$mS=sed.sum.by.phybioclus[,4]#mS=CC0.25
sed.sum.by.phybioclus$cS=rowSums(sed.sum.by.phybioclus[,5:6])#cS=C0.5+C1
sed.sum.by.phybioclus$fG=rowSums(sed.sum.by.phybioclus[,7:8])#fG=C2+C4
sed.sum.by.phybioclus$mG=sed.sum.by.phybioclus[,9]#mG=C8
sed.sum.by.phybioclus$cG=rowSums(sed.sum.by.phybioclus[,10:12])#cG=C16+C32+C64
sed.sum.by.phybioclus$TotalSiltClay=sed.sum.by.phybioclus[,1]#
sed.sum.by.phybioclus$TotalSand=rowSums(sed.sum.by.phybioclus[,2:6])#
sed.sum.by.phybioclus$TotalGravel=rowSums(sed.sum.by.phybioclus[,7:12])#

## Check sedient summary varaibles present.
names(sed.sum.by.phybioclus)

## Identify the number of samples belonging to each PhyBio cluster group
table(sed.sum.by.phybioclus$PhyBioCluster)

## Produce a df for mean sediment composition for each PhyBioCluster group
sed.sum.by.phybioclus.means=aggregate(sed.sum.by.phybioclus[, c(16:22,25:23)],
                                      list(sed.sum.by.phybioclus$PhyBioCluster),mean)

## Change name of col1 to PhyBioCluster
colnames(sed.sum.by.phybioclus.means)[1] <- "PhyBioCluster"

## Output data to a .csv file
write.csv(sed.sum.by.phybioclus.means, file="OUTPUTS/TABLE S2.csv",row.names=FALSE)

## Get sediment descriptions from 'Particle size distribution classification.xls'
# in C5922/WORKSPACE/PAPER

#################################################################################################
### STEP REF:       59                                                                        ###
###                                                                                           ###
### PAPER SECTION:  METHODS \ Faunal-Sediment relationships \ Sediment composition by faunal  ###
###                 and faunal-physical cluster groups                                        ###
###                 RESULTS \ Faunal-Sediment relationships \ Sediment composition by faunal  ###
###                 and faunal-physical cluster groups                                        ###
###                                                                                           ###
### TASK:           Figure 9 (parts 9a and 9b combined)                                       ###
###                                                                                           ###
### NOTES:          Use this code if a plot combining parts 9a and 9b is required             ###
#################################################################################################

require(cowplot)
```

```
## Save plot to an image file (png or tiff).
png("OUTPUTS/FIGURE 9.png",width=37, height=22, units="cm", res=1000) # works ok
#tiff("OUTPUTS/FIGURE 9.tiff",width=37, height=22, units="cm", res=1000)
plot_grid(fig9a,fig9b, labels = c("a)","b)"),nrow = 1,label_size = 14,rel_widths = c(1, 1.15))
dev.off()

###############################################################################################
# # #                               (G) GRAVEL/RICHNESS CHECK                               # # #
###############################################################################################

###############################################################################################
### STEP:             60                                                                    ###
###                                                                                         ###
### PAPER SECTION:    METHODS \ Faunal-sediment relationships \ Relationship between gravel   ###
###                   and taxon richness/total abundance                                    ###
###                   RESULTS \ Faunal-sediment relationships \ Relationship between gravel   ###
###                   and taxon richness/total abundance                                    ###
###                                                                                         ###
### TASK:             Prepare data for Richness by % gravel assessment                      ###
###############################################################################################

## Vector of Richness values
Richnessdf=as.data.frame(Richness)

## Vector of Abundance values
Abundancedf=as.data.frame(Abundance)

## Check number of records in dfs'Richnessdf', 'Abundancedf' and 'data4.5 - should be the same
# (27432)
dim(Richnessdf)
dim(Abundancedf)
dim(data4.5)

## df for sample labels
sample=data4.5[,1, drop=FALSE]

## Check dimensions of df 'sample'
dim(sample) #27432

## Join sample names to df 'richnessdf'
Richnessdf2=cbind(Richnessdf,Abundancedf,sample)
#View(Richnessdf2)

## Start with df 'data.new3' and select cols: Sample, Sieves, and cluster groupings
SampleSieveClusters=data.new3[,c(1,874:776,902,903,905)]

## Check names of df 'SampleSieveClusters'
names(SampleSieveClusters)

## Check dimensions of df 'SampleSieveClusters'
dim(SampleSieveClusters)#27432 103

## Check data types
str(SampleSieveClusters,list.len=ncol(SampleSieveClusters))

## Change an 'NA' values in sieve data to zero
SampleSieveClusters[, 2:100][is.na(SampleSieveClusters[, 2:100])] <- 0

## Add in cols for Mud, Sand and Gravel
SampleSieveClusters$Mud=rowSums(SampleSieveClusters[2:40])#Pan to 0.0625
SampleSieveClusters$Sand=rowSums(SampleSieveClusters[41:67]) #0.063 to 1.7
SampleSieveClusters$Gravel=rowSums(SampleSieveClusters[68:100])#2 to 125
SampleSieveClusters$CoarseGravel=rowSums(SampleSieveClusters[78:100])#10mm to 125
SampleSieveClusters$TotalPercent=rowSums(SampleSieveClusters[2:100])

## Check new cols added
names(SampleSieveClusters)#they are

## Select cols for Sample (1), sediment summary variables (Mud(104), Sand (105), Gravel (106),
# Coarse Gravel (107), TotalPercent (108)) and cluster groups (PhyCluster (101),
# FaunalCluster (102) and PhyBioCluster (103))
SampleSieveClusterssmall=SampleSieveClusters[,c(1,101:108)]

## Create a df with Richness and Abundance values, sediment and cluster data
Richnessdf3=cbind(Richnessdf2,SampleSieveClusterssmall)
```

```
## Check names of df 'Richnessdf3'
names(Richnessdf3)

## subset data for total percent is ~100
Richnessdf4 <- subset(Richnessdf3, TotalPercent > 99 & TotalPercent < 101)

## View df 'Richnessdf4'
#View(Richnessdf4)

## Add new cols in df datasc for variable of interest
Richnessdf4$Latitude=NA
Richnessdf4$Longitude=NA
Richnessdf4$Survey=NA
Richnessdf4$Gear=NA
Richnessdf4$TopSieveEmpty=NA
Richnessdf4$PSASubSample=NA
Richnessdf4$IncCol=NA
Richnessdf4$Sieve=NA
Richnessdf4$Treatment=NA

## Populate empty cols using data in df test (matching by col 'Sample')
names(data4.5)
Richnessdf4$Latitude<-data4.5[match(Richnessdf4$Sample, data4.5$Sample),807]
Richnessdf4$Longitude<-data4.5[match(Richnessdf4$Sample, data4.5$Sample),808]
Richnessdf4$Survey<-data4.5[match(Richnessdf4$Sample, data4.5$Sample),806]
Richnessdf4$Gear<-data4.5[match(Richnessdf4$Sample, data4.5$Sample),809]
Richnessdf4$TopSieveEmpty<-data4.5[match(Richnessdf4$Sample, data4.5$Sample),826]
Richnessdf4$PSASubSample<-data4.5[match(Richnessdf4$Sample, data4.5$Sample),815]
Richnessdf4$IncCol<-data4.5[match(Richnessdf4$Sample, data4.5$Sample),825]
Richnessdf4$Sieve<-data4.5[match(Richnessdf4$Sample, data4.5$Sample),810]
Richnessdf4$Treatment<-data4.5[match(Richnessdf4$Sample, data4.5$Sample),816]

dim(Richnessdf4)#23487
## Select data only where top sieve empty
Richnessdf5=subset(Richnessdf4, TopSieveEmpty=="Yes"|TopSieveEmpty=="NA")
dim(Richnessdf5)# 21391

## Select data only where PSA taken as a subsample from the faunal grab
Richnessdf6=subset(Richnessdf5,PSASubSample=="Yes")
dim(Richnessdf6)# 19788

## Select data only where sample taken using a comparable gear type.
Richnessdf7=subset(Richnessdf6, Gear=="MHN"|Gear=="DG"|Gear=="VV"|Gear=="SM")
dim(Richnessdf7)# 19788

## Select data only were sample processed over a 1mm sieve
Richnessdf8=subset(Richnessdf7, Sieve=='1')
dim(Richnessdf8)# 19788

## Select data only were samples include colonial taxa
Richnessdf9=subset(Richnessdf8, IncCol=="Yes")
dim(Richnessdf9)# 17074

## Select only data from 'Reference' conditions
Richnessdf10=subset(Richnessdf9, Treatment=="R"|Treatment=="NA")
dim(Richnessdf10)# 15127

## Complete cases only
Richnessdf11=Richnessdf10[complete.cases(Richnessdf10), ]
dim(Richnessdf11)#14740

## Select only cols of interest
names(Richnessdf11)
Richnessdf12=Richnessdf11[,c(3,1,2,10,6,15,13,14)]

## View df 'Richnessdf12'
#View(Richnessdf12)

## Save df 'Richnessdf12'
#write.csv(Richnessdf12,file = "OUTPUTS/Richnessdf12.csv",row.names=TRUE)

###############################################################################################
### STEP:             61                                                                    ###
###                                                                                         ###
### PAPER SECTION:    METHODS \ Faunal-sediment relationships \ Relationship between gravel  ###
###                   and taxon richness/total abundance                                    ###
```

```
###                 RESULTS \ Faunal-sediment relationships \ Relationship between gravel   ###
###                 and taxon richness/total abundance                                      ###
###                                                                                         ###
### TASK:           Calculate regression coefficients for all data                          ###
###############################################################################################

names(Richnessdf12)
gravel = Richnessdf12$Gravel
cluster = Richnessdf12$FaunalCluster
rich = Richnessdf12$Richness
abun = Richnessdf12$Abundance
survey = Richnessdf12$Survey
lat = Richnessdf12$Latitude
long = Richnessdf12$Longitude
table(cluster)# Number of samples by faunal cluster group

#install.packages("nlme")
library(nlme)

## Calculates empirical autocorrelation function for within-group residuals from an lme fit
acf(rich)

lm2 = lm(rich ~ gravel + survey)
res = lm2$residuals
acf(res)

alpha.all = lm2$coefficients[1]
beta.all = lm2$coefficients[2]
anova(lm2)[1,5]           # < 2*10 ^ (-16)

## Intercept from median survey (use this for calc regression line)
med.coef = median(c(0, lm2$coefficients[-c(1,2)]))
alpha.all.med = alpha.all + med.coef

## Plot data and regression line
plot(gravel,rich)
gravplot = c(min(gravel), max(gravel))
yplot=alpha.all.med + beta.all * gravplot
lines(gravplot, yplot, col=2)

## Find min gravel value
minXall=min(gravel)

## Find max gravel values by faunal cluster group
maxXall=max(gravel)

## Create object 'intercept'
intercept=alpha.all.med

## Create object 'slope'
slope=beta.all

## Create a df for FaunalCluster, min X, max X, intercept and slope
reg.data.all=data.frame(minXall, maxXall, intercept,slope)

## Change col names in df 'reg.data2.all'
colnames(reg.data.all)=c("Xmin","Xmax","Intercept","Slope")
View(reg.data.all)

## Create new cols for Ymax and Ymin
reg.data.all$Ymax=NA
reg.data.all$Ymin=NA

## Calculate Ymax and Ymin using regression equation Y=a+bx
reg.data.all$Ymax=reg.data.all$Intercept+(reg.data.all$Slope*reg.data.all$Xmax)
reg.data.all$Ymin=reg.data.all$Intercept+(reg.data.all$Slope*reg.data.all$Xmin)

## find n for df 'richnessdf12'
summary(Richnessdf12)# Answer: 14740 - insert this value in annotate instruction below

###############################################################################################
### STEP:           62                                                                      ###
###                                                                                         ###
### PAPER SECTION:  METHODS \ Faunal-sediment relationships \ Relationship between gravel   ###
###                 and taxon richness/total abundance                                      ###
###                 RESULTS \ Faunal-sediment relationships \ Relationship between gravel   ###
```

```
###                    and taxon richness/total abundance                                      ###
###                                                                                            ###
### TASK:              Figure 10a (Richness by % gravel plot)                                  ###
##################################################################################################

library(ggplot2)

## Gravel vs Richness plot (all samples). Use annotate to place equation and r^2 on plot
RichGrav <- ggplot(data = Richnessdf12, aes(x = Gravel, y = Richness)) +
  geom_point(data = Richnessdf4, aes(x = Gravel, y = Richness,col=FaunalCluster),shape=16,
             alpha=0.7, size=0.9)+
  scale_colour_manual(values = c("blue2","cyan1","#05aae1","plum2","darkorchid3","green3",
                                 "palegreen1","#b40202","red1","darkorange","yellow",
                                 "#b4b404"),name="Cluster")+
    guides(colour = guide_legend(override.aes = list(size=3)))+ # Change size of legend dots
  geom_segment(aes(x=Xmin,y=Ymin,xend=Xmax,yend=Ymax), data=reg.data.all,size=1.1) +
  labs(x = "% Gravel")+
  theme_bw(base_size=14)

fig10a=RichGrav+theme(legend.position="none")+
  annotate("text", x = 50, y = 110,
           label = "italic(n)==14740~~italic(\u03B2)==0.37^'***'",parse = TRUE,size=4.5)

## Save plot to an image file (png or tiff)
png("OUTPUTS/FIGURE 10a.png",width =20 ,height = 21,units = "cm",res = 600,pointsize = 12)
#tiff("OUTPUTS/FIGURE 10a.tiff",width =20,height = 21,units = "cm",res = 600,pointsize = 12)
fig10a
dev.off()

##################################################################################################
### STEP:              63                                                                      ###
###                                                                                            ###
### PAPER SECTION:     METHODS \ Faunal-sediment relationships \ Relationship between gravel   ###
###                    and taxon richness/total abundance                                      ###
###                    RESULTS \ Faunal-sediment relationships \ Relationship between gravel   ###
###                    and taxon richness/total abundance                                      ###
###                                                                                            ###
### TASK:              Regression calcs for individual cluster groups                          ###
##################################################################################################

names(Richnessdf12)
gravel = Richnessdf12$Gravel
cluster = Richnessdf12$FaunalCluster
rich = Richnessdf12$Richness
abun = Richnessdf12$Abundance
survey = Richnessdf12$Survey
lat = Richnessdf12$Latitude
long = Richnessdf12$Longitude
table(cluster)

cluster.names = c("A1","A2a","A2b","B1a","B1b","C1a","C1b","D1",
                  "D2a","D2b","D2c","D2d")

#install.packages("nlme")
library(nlme)

alpha = rep(0,12)
alpha.adj = rep(0,12)
beta = rep(0,12)
pvalues = rep(0,12)

for (j in c(1:12)) {

  gravel.D1 = gravel[cluster==cluster.names[j]]
  rich.D1 = rich[cluster==cluster.names[j]]
  survey.D1 = survey[cluster==cluster.names[j]]

  lm1 = lm(rich.D1 ~ gravel.D1 + survey.D1)
  res = lm1$residuals
  acf(res)
  pvalues[j] = anova(lm1)[1,5]
  alpha[j] = lm1$coefficients[1]
  alpha.adj[j] = alpha[j] + median(c(0,lm1$coefficients[-c(1,2)]))
  beta[j] = lm1$coefficients[2]
}
```

```
## Plot data and regression lines by faunal cluster group
par(mfrow=c(3,2), mar=c(3,3,3,3))
for (j in 7:12) {
  gravel.D1 = gravel[cluster==cluster.names[j]]
  rich.D1 = rich[cluster==cluster.names[j]]
  survey.D1 = survey[cluster==cluster.names[j]]
  plot(gravel.D1, rich.D1)
  gravplot = c(min(gravel.D1), max(gravel.D1))
  yplot = alpha.adj[j] + beta[j] * gravplot
  lines(gravplot, yplot, col=2)
}

## See regression coefficients
alpha.adj
beta
pvalues

## Find max gravel values by faunal cluster group
require(dplyr)
minX=Richnessdf12 %>% group_by(FaunalCluster) %>% summarise(Gravel = min(Gravel))

## Find max gravel values by faunal cluster group
maxX=Richnessdf12 %>% group_by(FaunalCluster) %>% summarise(Gravel = max(Gravel))

## Create object 'intercept' for values of alpha by faunal cluster group
intercept=alpha.adj

## Create object 'slope' for values of beta by faunal cluster group
slope=beta

## Create a df for FaunalCluster, min X, max X, intercept and slope
reg.data=data.frame(minX, maxX, intercept,slope)

## Select only cols of interest
names(reg.data)
reg.data2=reg.data[,c(1,2,4,5,6)]

## Change col names
colnames(reg.data2)=c("FaunalCluster","Xmin","Xmax","Intercept","Slope")
View(reg.data2)

## Create new cols for Ymax and Ymin
reg.data2$Ymax=NA
reg.data2$Ymin=NA

## Calculate Ymax and Ymin using regression equation Y=a+bx
reg.data2$Ymax=reg.data2$Intercept+(reg.data2$Slope*reg.data2$Xmax)
reg.data2$Ymin=reg.data2$Intercept+(reg.data2$Slope*reg.data2$Xmin)

###################################################################################################
### STEP:             64                                                                        ###
###                                                                                             ###
### PAPER SECTION:    METHODS \ Faunal-sediment relationships \ Relationship between gravel    ###
###                   and taxon richness/total abundance                                        ###
###                   RESULTS \ Faunal-sediment relationships \ Relationship between gravel    ###
###                   and taxon richness/total abundance                                        ###
###                                                                                             ###
### TASK:             Figure 10b (Richness by % gravel plot for different faunal cluster       ###
###                   groups)                                                                   ###
###################################################################################################

## Change Richnessdf4$FaunalCluster from chr to a factor
Richnessdf12$FaunalCluster=as.factor(Richnessdf12$FaunalCluster)

## Create a df for the regression data
abline.dat <- data.frame(FaunalCluster=levels(Richnessdf12$FaunalCluster),
                         intercept=c(67.472812, 53.201367, 59.113773, 55.324739, 44.284760,
                                     30.933622, 43.556371, 35.956208, 20.339853, 26.473710,
                                     8.111198, 17.947759),
                         slope=c(-0.02546518, -0.06716964, -0.05092130,  0.11158370,
                                 0.04067788,  0.01542932,  0.01753884,  0.44454995,
                                 0.08889163, 0.09824444,  0.07805096,  0.13021108))

## Gravel vs Richness plot, faceted by faunal cluster group (inc equation and r2)
RichGravbyFCG <- ggplot(data = Richnessdf12, aes(x = Gravel, y = Richness)) +
  geom_point(data = Richnessdf12, aes(x = Gravel, y = Richness,col=FaunalCluster),shape=16,
```

```
             alpha=0.5, size=0.9)+
  scale_colour_manual(values = c("blue2","cyan1","#05aae1","plum2","darkorchid3","green3",
                               "palegreen1","#b40202","red1","darkorange","yellow",
                               "#b4b404"), name="Cluster")+
  geom_segment(aes(x=Xmin,y=Ymin,xend=Xmax,yend=Ymax), data=reg.data2,size=1.1) +
  facet_wrap(~FaunalCluster,nrow=4)+
  labs(x = "% Gravel")+
  theme_bw(base_size=14)

fig10b=RichGravbyFCG+
  theme(legend.position="none")+
  annotate("text", x = 50, y = 110,
         label = c("italic(n)==297~~italic(\u03B2)==-0.03^'(p = 0.13)'",
                   "italic(n)==514~~italic(\u03B2) == -0.07^'***'",
                   "italic(n)==813~~italic(\u03B2) == -0.05^'***'",
                   "italic(n)==1058~~italic(\u03B2) == 0.11^'***'",
                   "italic(n)==776~~italic(\u03B2) == 0.04^'(p = 0.66)'",
                   "italic(n)==1501~~italic(\u03B2) == 0.02^'(p = 0.76)'",
                   "italic(n)==1180~~italic(\u03B2) == 0.02^'(p = 0.30)'",
                   "italic(n)==319~~italic(\u03B2) == 0.44^'***'",
                   "italic(n)==1832~~italic(\u03B2) == 0.09^'***'",
                   "italic(n)==997~~italic(\u03B2) == 0.10^'***'",
                   "italic(n)==4065~~italic(\u03B2) == 0.08^'***'",
                   "italic(n)==1388~~italic(\u03B2) == 0.13^'***'"),
                   parse = TRUE,size=4.5)

## Save plot to an image file (png or tiff)
png("OUTPUTS/FIGURE 10b.png", width =20 , height = 21, units = "cm", res = 600, pointsize = 12)
#tiff("OUTPUTS/FIGURE 10b.tiff", width =20 , height = 21, units = "cm", res = 600, pointsize = 12)

fig10b

dev.off()

###############################################################################################
### STEP REF:       65                                                                      ###
###                                                                                         ###
### PAPER SECTION:  RESULTS \ Faunal-sediment relationships \ Relationship between gravel   ###
###                 and taxon richness/total abundance                                      ###
###                                                                                         ###
### TASK:           Figure 10 Parts a & b combined (Taxon Richness by % Gravel)             ###
###                                                                                         ###
### NOTES:          Use this code if a plot combining parts a and b is required             ###
###############################################################################################

require(cowplot)

## Save plot to an image file (png or tiff).

png("OUTPUTS/FIGURE 10.png",width=39, height=22, units="cm", res=1000) # works ok
#tiff("OUTPUTS/FIGURE 10.tiff",width=39, height=22, units="cm", res=1000)
plot_grid(fig10a,fig10b, labels = c("a)","b)"),nrow = 1,label_size = 14,scale = 0.98,
          hjust=0.04)#rel_widths = c(1, 1.15), rel_heights=c(0.05, 3)
dev.off()

###############################################################################################
# # #                    (H) ASSESSING SEDIMENT CHANGE (MAHALANOBIS)                        # # #
###############################################################################################

###############################################################################################
### STEP:             66                                                                    ###
###                                                                                         ###
### PAPER SECTION:    METHODS \ Assessing the ecological significance of sediment change    ###
###                   RESULTS \ Assessing the ecological significance of sediment change    ###
###                                                                                         ###
### TASK:             Table 4 (Mahalanobis Calcs)                                           ###
###############################################################################################

## Use this code to avoid having to run code above. The df 'sed.sum.by.fclus' contains the
# necessary data for the Mahalanobis calcs (sieve data (used in cum sed plots), summary data
# (coarse, med fine), summary data (total G, S and SC) and cluster groups (FaunalCluster,
# PhyCluster and PhyBioCluster))
sed.sum.by.fclus=read.csv("OUTPUTS/SEDSUMBYFCLUS.csv", header=T,
                          na.strings=c("NA", "-", "?","<null>"),stringsAsFactors=F)

## View df 'sed.sum.by.fclus'
```

```
#View(sed.sum.by.fclus)

## View names for df 'sed.sum.by.fclus'.
names(sed.sum.by.fclus)

##  Select only the data of interest (summary data and PhyBioCluster)
mdata=sed.sum.by.fclus[,c(15,16:22)]

## Check cols in df 'mdata'
names(mdata)

## Subset by PhyBioCluster grp. See file: OUTPUTS/datanew.csv for cluster group identity. Note
# that sample EEC2010_Site 102  has a baseline faunal cluster identity of 4_B1a
# (Sample: EECREA2005#102#102#19278).
mdata2= subset(mdata,PhyBioCluster=="4_B1a") # sample EEC2010_Site 102

## Identify number of available replicates
dim(mdata2)

## Check df 'mdata2' has only samples of chosen PhyBioCluster group
#View(mdata2)

#Subset for numeric variables (i.e. sediment variables only)
mdata3=mdata2[,8:2]

## Check df 'mdata3' has only sediment variables
#View(mdata3)

## Sediment data for test sample EEC2010_Site 102:
#cG=3.11892
#mG=3.85611
#fG=13.36363
#cS=23.68595
#mS=36.31295
#fS=17.79785
#S/C=1.86459

## Function
quantile = function(X, mu, sigma) {
  md = mahalanobis(X, mu, sigma)
  pvalue = 1 - pchisq(md,length(X))
  pvalue
}

## Set up parameters using training data set (df 'mdata3') - Mahalanobis needs these.
sigma = cov(mdata3)
mu = colMeans(mdata3)
nrows = dim(mdata3)[1]

## Monitoring dataset - define number of rows and cols. Change nrow number where multiple
# samples
data.monitor = matrix(0, ncol=7, nrow=1)

## Input monitoring sample data. Order from coarse gravel to silt/clay. Data below for sample:
# EEC2010_Site 102
data.monitor[1,] = c(3.11892,3.85611,13.36363,23.68595,36.31295,17.79785,1.86459)

## Use this to do multiple samples at the same time. Remember to change nrow above accordingly
#data.monitor[1,] = c()
#data.monitor[2,] = c()
#data.monitor[3,] = c()

## Run test
nmons = dim(data.monitor)[1]
ps = rep(0, nmons)

for (j in 1:nmons) {
  ps[j] = quantile(data.monitor[j,], mu, sigma)
}

## Pick off the ones that fail. FALSE means the sample is OK (i.e. acceptable sediment
# composition)
fail = ps<0.05
fail

###################################################################################################
```

```
### STEP REF:       67                                                                   ###
###                                                                                    ###
### PAPER SECTION:  Supplementary Info (Further testing of Mahalanobis)                ###
###                                                                                    ###
### TASK:           Extract Area 222 data for Mahalanobis testing                      ###
##########################################################################################

## First create a df for the list of Area 222 samples. The df contains cols for 'Sample',
#'StationCode', 'Year'(NB/ years 2001, 2002, 2003, 2004, 2005, 2007, 2011 relate to 5,6,7,8,
# 11 and 15 years after dredging) and 'Treatement' (high or ref).
Area222data=data.frame(Sample=c("CEFAS_MHN_3103","CEFAS_MHN_3104","CEFAS_MHN_3105",
                                "CEFAS_MHN_3106","CEFAS_MHN_3107","CEFAS_MHN_3108",
                                "CEFAS_MHN_3109","CEFAS_MHN_3110","CEFAS_MHN_3111",
                                "CEFAS_MHN_3112","CEFAS_MHN_2969","CEFAS_MHN_2970",
                                "CEFAS_MHN_2971","CEFAS_MHN_2972","CEFAS_MHN_2973",
                                "CEFAS_MHN_2974","CEFAS_MHN_2975","CEFAS_MHN_2976",
                                "CEFAS_MHN_2977","CEFAS_MHN_2978","CEFAS_MHN_4680",
                                "CEFAS_MHN_4681","CEFAS_MHN_4682","CEFAS_MHN_4683",
                                "CEFAS_MHN_4684","CEFAS_MHN_4685","CEFAS_MHN_4686",
                                "CEFAS_MHN_4687","CEFAS_MHN_4688","CEFAS_MHN_4689",
                                "CEFAS_MHN_5948","CEFAS_MHN_5949","CEFAS_MHN_5950",
                                "CEFAS_MHN_5951","CEFAS_MHN_5952","CEFAS_MHN_5953",
                                "CEFAS_MHN_5954","CEFAS_MHN_5955","CEFAS_MHN_5956",
                                "CEFAS_MHN_5957","CEND1207222#R2A#183#21604",
                                "CEND1207222#R2B#184#21605","CEND1207222#R2C#187#21606",
                                "CEND1207222#R2D#186#21607","CEND1207222#R2E#185#21608",
                                "CEND1207222#R1D#208#21602","CEND1207222#R1E#209#21603",
                                "CEND1207222#R1B#210#21600","CEND1207222#R1C#211#21601",
                                "CEND1207222#R1A#212#21599",NA,NA,NA,NA,NA,NA,NA,NA,NA,NA,
                                "CEFAS_MHN_3079","CEFAS_MHN_3080","CEFAS_MHN_3081",
                                "CEFAS_MHN_3082","CEFAS_MHN_3083","CEFAS_MHN_3084",
                                "CEFAS_MHN_3085","CEFAS_MHN_3086","CEFAS_MHN_3087",
                                "CEFAS_MHN_3089","CEFAS_MHN_2949","CEFAS_MHN_2950",
                                "CEFAS_MHN_2951","CEFAS_MHN_2952","CEFAS_MHN_2953",
                                "CEFAS_MHN_2954","CEFAS_MHN_2955","CEFAS_MHN_2956",
                                "CEFAS_MHN_2957","CEFAS_MHN_2958","CEFAS_MHN_4660",
                                "CEFAS_MHN_4661","CEFAS_MHN_4662","CEFAS_MHN_4663",
                                "CEFAS_MHN_4664","CEFAS_MHN_4665","CEFAS_MHN_4666",
                                "CEFAS_MHN_4667","CEFAS_MHN_4668","CEFAS_MHN_4669",
                                "CEFAS_MHN_5927","CEFAS_MHN_5928","CEFAS_MHN_5929",
                                "CEFAS_MHN_5930","CEFAS_MHN_5932","CEFAS_MHN_5933",
                                "CEFAS_MHN_5934","CEFAS_MHN_5935","CEFAS_MHN_5936",
                                "CEFAS_MHN_5937","CEND1207222#H1#188#21579",
                                "CEND1207222#H2#189#21580","CEND1207222#H6#190#21584",
                                "CEND1207222#H10#191#21588","CEND1207222#H5#192#21583",
                                "CEND1207222#H8#193#21586","CEND1207222#H3#194#21581",
                                "CEND1207222#H7#195#21585","CEND1207222#H9#196#21587",
                                "CEND1207222#H4#197#21582",NA,NA,NA,NA,NA,NA,NA,NA,NA,NA),
                       StationCode=c("CIR 5X01 222_68_R1A","CIR 5X01 222_69_R1B",
                                     "CIR 5X01 222_70_R1D","CIR 5X01 222_110_R2C",
                                     "CIR 5X01 222_111_R2A","CIR 5X01 222_112_R2D",
                                     "CIR 5X01 222_113_R2B","CIR 5X01 222_114_R2E",
                                     "CIR 5X01 222_67_R1E","CIR 5X01 222_71_R1C",
                                     "CIR402222#65#R1A#2969","CIR402222#66#R1B#2970",
                                     "CIR402222#67#R1C#2971","CIR402222#64#R1D#2972",
                                     "CIR402222#68#R1E#2973","CIR402222#33#R2A#2974",
                                     "CIR402222#35#R2B#2975","CIR402222#32#R2C#2976",
                                     "CIR402222#30#R2D#2977","CIR402222#34#R2E#2978",
                                     "END503222#60#R1A#4680","END503222#63#R1B#4681",
                                     "END503222#61#R1C#4682","END503222#64#R1D#4683",
                                     "END503222#62#R1E#4684","END503222#59#R2A#4685",
                                     "END503222#58#R2B#4686","END503222#57#R2C#4687",
                                     "END503222#56#R2D#4688","END503222#55#R2E#4689",
                                     "COR11X222#52#R1A#5948","COR11X222#55#R1B#5949",
                                     "COR11X222#54#R1C#5950","COR11X222#53#R1D#5951",
                                     "COR11X222#56#R1E#5952","COR11X222#57#R2A#5953",
                                     "COR11X222#60#R2B#5954","COR11X222#59#R2C#5955",
                                     "COR11X222#58#R2D#5956","COR11X222#61#R2E#5957",
                                     "Area 222 2007 R2A","Area 222 2007 R2B",
                                     "Area 222 2007 R2E","Area 222 2007 R2D",
                                     "Area 222 2007 R2C","Area 222 2007 R1D",
                                     "Area 222 2007 R1E","Area 222 2007 R1B",
                                     "Area 222 2007 R1C","Area 222 2007 R1A",
                                     "Area 222 2011 R2_1","Area 222 2011 R2_2",
                                     "Area 222 2011 R2_3","Area 222 2011 R2_4",
                                     "Area 222 2011 R2_5","Area 222 2011 R1_1",
```

```
                                "Area 222 2011 R1_2","Area 222 2011 R1_3",
                                "Area 222 2011 R1_4","Area 222 2011 R1_5",
                                "CIR 5X01 222_32_H7","CIR 5X01 222_34_H13",
                                "CIR 5X01 222_35_H2","CIR 5X01 222_36_H14",
                                "CIR 5X01 222_37_H8","CIR 5X01 222_38_H10",
                                "CIR 5X01 222_39_H3","CIR 5X01 222_40_H12",
                                "CIR 5X01 222_41_H11","CIR 5X01 222_43_H4",
                                "CIR402222#36#H1#2949","CIR402222#46#H10#2950",
                                "CIR402222#38#H2#2951","CIR402222#43#H3#2952",
                                "CIR402222#37#H4#2953","CIR402222#42#H5#2954",
                                "CIR402222#41#H6#2955","CIR402222#39#H7#2956",
                                "CIR402222#45#H8#2957","CIR402222#44#H9#2958",
                                "END503222#50#H1#4660","END503222#53#H10#4661",
                                "END503222#43#H2#4662","END503222#45#H3#4663",
                                "END503222#46#H4#4664","END503222#54#H5#4665",
                                "END503222#48#H6#4666","END503222#47#H7#4667",
                                "END503222#49#H8#4668","END503222#44#H9#4669",
                                "COR11X222#39#H1#5927","COR11X222#41#H10#5928",
                                "COR11X222#34#H2#5929","COR11X222#40#H3#5930",
                                "COR11X222#32#H4#5932","COR11X222#38#H5#5933",
                                "COR11X222#35#H6#5934","COR11X222#37#H7#5935",
                                "COR11X222#33#H8#5936","COR11X222#36#H9#5937",
                                "CEND 12/07_H1","CEND 12/07_H2","CEND 12/07_H6",
                                "CEND 12/07_H10","CEND 12/07_H5","CEND 12/07_H8",
                                "CEND 12/07_H3","CEND 12/07_H7","CEND 12/07_H9",
                                "CEND 12/07_H4","Area 222 2011 H1","Area 222 2011 H2",
                                "Area 222 2011 H3","Area 222 2011 H4","Area 222 2011 H5",
                                "Area 222 2011 H6","Area 222 2011 H7","Area 222 2011 H8",
                                "Area 222 2011 H9","Area 222 2011 H10"),
                    Year=c(2001,2001,2001,2001,2001,2001,2001,2001,2001,2001,2002,2002,2002,
                           2002,2002,2002,2002,2002,2002,2002,2003,2003,2003,2003,2003,2003,
                           2003,2003,2003,2003,2004,2004,2004,2004,2004,2004,2004,2004,2004,
                           2004,2007,2007,2007,2007,2007,2007,2007,2007,2007,2007,2011,2011,
                           2011,2011,2011,2011,2011,2011,2011,2011,2001,2001,2001,2001,2001,
                           2001,2001,2001,2001,2001,2002,2002,2002,2002,2002,2002,2002,2002,
                           2002,2002,2003,2003,2003,2003,2003,2003,2003,2003,2003,2003,2004,
                           2004,2004,2004,2004,2004,2004,2004,2004,2004,2007,2007,2007,2007,
                           2007,2007,2007,2007,2007,2007,2011,2011,2011,2011,2011,2011,2011,
                           2011,2011,2011),
                    Treatment=c("Ref","Ref","Ref","Ref","Ref","Ref","Ref","Ref","Ref","Ref",
                                "Ref","Ref","Ref","Ref","Ref","Ref","Ref","Ref","Ref","Ref",
                                "Ref","Ref","Ref","Ref","Ref","Ref","Ref","Ref","Ref","Ref",
                                "Ref","Ref","Ref","Ref","Ref","Ref","Ref","Ref","Ref","Ref",
                                "Ref","Ref","Ref","Ref","Ref","Ref","Ref","Ref","Ref","Ref",
                                "Ref","Ref","Ref","Ref","Ref","Ref","Ref","Ref","Ref","Ref",
                                "High","High","High","High","High","High","High","High",
                                "High","High","High","High","High","High","High","High",
                                "High","High","High","High","High","High","High","High",
                                "High","High","High","High","High","High","High","High",
                                "High","High","High","High","High","High","High","High",
                                "High","High","High","High","High","High","High","High",
                                "High","High","High","High","High","High","High","High",
                                "High","High","High","High"))

## Change data types in df 'Area222data'
Area222data$StationCode=as.character(Area222data$StationCode)
Area222data$Sample=as.character(Area222data$Sample)
Area222data$Year=as.factor(Area222data$Year)

## View df 'Area222data'
View(Area222data)

## Need to run script for steps: 1 (load df 'dataall2'), 7, 16, 17, 18 (1st 2 rows only), 19,
# 21, 23, 42, 44, 50. Data fields to extract include: Richness, Abundance, Lat, Long, PhyCluster,
# FaunalCluster, PhyBioCluster, GrabSampleSize, SC, fS, mS, cS, fG, mG, cG.

## Above data stored in various dataframes and some manipulation may be required using code below
#   DATAFRAME         FROM STEP     VARIABLES
#   univmeascoord     18            Lat (2), Long (3), Richness (4), Abundance (5)
#   faunal.cluster    23            FaunalCluster (5)
#   data.new          50            PhyCluster (901), PhyBioCluster (904)
#   dataall2          1             GrabSampleSize (13465)

## Add new cols in df 'Area222data' for variables of interest
Area222data$Richness=NA
Area222data$Abundance=NA
```

```
Area222data$Lat=NA
Area222data$Long=NA
Area222data$PhyCluster=NA
Area222data$FaunalCluster=NA
Area222data$PhyBioCluster=NA
Area222data$GrabSampleSize=NA

## Populate empty cols in df 'Area222data' using data from other dataframes (matching by col
# 'Sample')
Area222data$Richness<-univmeascoord[match(Area222data$Sample, univmeascoord$Sample),4]
Area222data$Abundance<-univmeascoord[match(Area222data$Sample, univmeascoord$Sample),5]
Area222data$Lat<-univmeascoord[match(Area222data$Sample, univmeascoord$Sample),2]
Area222data$Long<-univmeascoord[match(Area222data$Sample, univmeascoord$Sample),3]
Area222data$FaunalCluster<-faunal.cluster[match(Area222data$Sample, faunal.cluster$Sample),5]
Area222data$PhyCluster<-data.new[match(Area222data$Sample, data.new$Sample),902]
Area222data$PhyBioCluster<-data.new[match(Area222data$Sample, data.new$Sample),905]
Area222data$GrabSampleSize<-dataall2[match(Area222data$Sample, dataall2$Sample),13465]

## View df 'Area222datav2'
View(Area222data)

## Now add in the sediment data. First create a df 'dataall2ss' which is a subset of
# df 'dataall2' for only the sample and sieve variables
options(max.print=1000000)# to see all column names
names(dataall2)
dataall2ss=dataall2[,c(2,13564:13466)]
names(dataall2ss)

## Now add sieve data from df 'dataall2ss'to df 'Area222data'
a=join(Area222data,dataall2ss,by = "Sample")
#View(a)
#names(a)

## Change 'NA' values in sieve data to zero (allowing for rowsum to produce summary variables)
a[,13:111][is.na(a[, 13:111])] <- 0

## Introduce new cols for sediment summary variables
a$SC=rowSums(a[13:51])#SC
a$fS=rowSums(a[52:62])#fS
a$mS=rowSums(a[63:69])#mS
a$cS=rowSums(a[70:78])#cS
a$fG=rowSums(a[79:87])#fG
a$mG=rowSums(a[88:92])#mG
a$cG=rowSums(a[93:111])#cG

## Select only cols of interest (Richness","Abundance","Lat","Long","PhyCluster","FaunalCluster"
#"PhyBioCluster","GrabSampleSize","SC","fS","mS","cS","fG","mG","cG"). In other words, get rid of
# the sieve data variables
names(a)
b=a[,c(1:12,112:118)]
#View(b)

## Now add a col 'MtestSedData' which is the sediment data (cG, mG, fG, cS, mS, fS, SC) ready for
# input in Mahalanobis test above
b$MtestSedData= do.call(paste, c(b[c("cG", "mG", "fG", "cS", "mS", "fS", "SC")], sep = ","))

## Insert missing sediment data for Area 222 2011 survey (NB these data are not part of the
# wider dataset)
b$MtestSedData[b$StationCode == "Area 222 2011 R2_1"] <-
  "14.811358485,36.14718038,12.872950274,20.32063876,7.287831074,4.239102911,4.320938119"
b$MtestSedData[b$StationCode == "Area 222 2011 R2_2"] <-
  "23.278824405,24.10250212,15.159468841,19.869704654,7.630631547,5.077835411,4.881033028"
b$MtestSedData[b$StationCode == "Area 222 2011 R2_3"] <-
  "5.037953624,8.843714256,14.89809712,29.633122178,6.965435687,7.813018301,26.808658834"
b$MtestSedData[b$StationCode == "Area 222 2011 R2_4"] <-
  "24.239301402,18.240494658,9.415848566,27.5653309,8.458932909,6.112961435,5.967130133"
b$MtestSedData[b$StationCode == "Area 222 2011 R2_5"] <-
  "30.243519249,11.66012045,6.276512176,23.90933978,9.662500995,7.923603404,10.324403949"
b$MtestSedData[b$StationCode == "Area 222 2011 R1_1"] <-
  "33.34046926,9.939630074,8.488182907,17.433419676,7.828502669,5.247050574,17.722744843"
b$MtestSedData[b$StationCode == "Area 222 2011 R1_2"] <-
  "17.423996186,13.070705758,13.371904049,22.189782731,10.149556083,6.146539384,17.647515812"
b$MtestSedData[b$StationCode == "Area 222 2011 R1_3"] <-
  "24.53199522,20.107505162,12.650499464,15.620035967,11.506600996,5.696678802,9.886684393"
b$MtestSedData[b$StationCode == "Area 222 2011 R1_4"] <-
  "0,15.046443781,8.014486435,17.370998505,5.924600631,8.611433962,45.032036685"
```

```
b$MtestSedData[b$StationCode == "Area 222 2011 R1_5"] <-
  "6.965118027,13.749812057,10.645015787,23.010869802,2.412456046,5.234051596,37.982676685"
b$MtestSedData[b$StationCode == "Area 222 2011 H1"] <-
  "19.818792162,12.062053906,8.84202979,11.178456449,7.391884839,8.005033032,32.70174982"
b$MtestSedData[b$StationCode == "Area 222 2011 H2"] <-
  "42.86266665,18.201839177,6.783450734,11.846192524,10.753279656,5.327124808,4.225446453"
b$MtestSedData[b$StationCode == "Area 222 2011 H3"] <-
  "23.959250774,17.344841754,7.410017028,13.201405232,17.196514664,12.524268981,8.36370156"
b$MtestSedData[b$StationCode == "Area 222 2011 H4"] <-
  "32.524442305,18.646385865,7.760548543,9.942942586,16.206984295,8.723622759,6.195073645"
b$MtestSedData[b$StationCode == "Area 222 2011 H5"] <-
  "38.533385949,13.76782996,11.422450246,18.279606701,10.386246154,3.808602438,3.801878548"
b$MtestSedData[b$StationCode == "Area 222 2011 H6"] <-
  "28.573592421,17.808456315,15.116631324,15.667161222,11.239338673,4.729723911,6.865096143"
b$MtestSedData[b$StationCode == "Area 222 2011 H7"] <-
  "2.357646825,7.68657458,7.222001392,24.151413042,30.73491074,17.95071099,9.896742425"
b$MtestSedData[b$StationCode == "Area 222 2011 H8"] <-
  "12.23989367,16.839306998,15.407003392,23.556032199,20.7117997,7.756356462,3.489607588"
b$MtestSedData[b$StationCode == "Area 222 2011 H9"] <-
  "16.401357522,27.845339587,22.213243909,15.372948488,8.733404387,6.231739001,3.201967112"
b$MtestSedData[b$StationCode == "Area 222 2011 H10"] <-
  "7.088228774,19.341609394,13.208453411,21.994976823,20.30710953,10.732908608,7.326713461"

## Check 2011 sed data added to col 'MtestSedData' in df 'b'
View(b)

## Now perform Mahalanobis tests on each sample in df 'b'. These samples come from ref and
# dredged (high) treaments over the period 2001 to 2011(i.e. years 5,6,7,8,11 and 15). The test
# data are contained in col b$MtestSedData (extracted out below for convenience). Note that as
# Area 222 samples were randomly selected from ref and dredged (high) boxes, we cannot know the
# original faunal cluster group identity at each station. We therefore assume a mix of faunal
# groups (8_A2b, 8_C1a, 8_C1b) based on those present within the ref sites (2001-2007).
# Monitoring sample sediments will be compared to sediments from these combined groups.

## Subset data for samples belonging to PhyBioCluster groups 8_A2b, 8_C1a and 8_C1b.
mdata2= subset(mdata,PhyBioCluster=="8_A2b"|
                 PhyBioCluster=="8_C1a"|
                 PhyBioCluster=="8_C1b")# Area 222 ref sites faunal identity

## Identify number of available replicates
dim(mdata2)

#Subset for numeric variables (i.e. sediment variables only)
mdata3=mdata2[,8:2]

## Function
quantile = function(X, mu, sigma) {
  md = mahalanobis(X, mu, sigma)
  pvalue = 1 - pchisq(md,length(X))
  pvalue
}

## Set up parameters using training data set (df 'mdata3') - Mahalanobis needs these.
sigma = cov(mdata3)
mu = colMeans(mdata3)
nrows = dim(mdata3)[1]

## Monitoring dataset - define number of rows and cols. Change nrow number where multiple samples
data.monitor = matrix(0, ncol=7, nrow=120)

## Input monitoring sample data. Order from coarse gravel to silt/clay
data.monitor[1,] = c(7.910617856,7.41660264,19.215598331,30.125366629,19.581683368,10.018699986,
                     5.731431192)
data.monitor[2,] = c(9.893249765,10.913816583,11.356409337,29.084101066,16.531274097,4.365248059,
                     17.855901092)
data.monitor[3,] = c(27.243048957,18.123496122,14.01935732,15.242171432,9.886578633,3.059858876,
                     12.425488664)
data.monitor[4,] = c(18.236034813,19.547737805,7.241273139,20.289512393,4.654879393,4.217834085,
                     25.812728367)
data.monitor[5,] = c(50.44506213,8.770770824,6.656857497,18.066829249,3.080180155,2.394088904,
                     10.586211242)
data.monitor[6,] = c(4.556633573,4.288779515,10.100589664,43.937016873,24.549908318,8.101769865,
                     4.465302191)
data.monitor[7,] = c(20.90185256,14.51132504,17.125849012,27.889849002,8.226566235,6.562854292,
                     4.781703857)
data.monitor[8,] = c(44.02423968,12.466269851,7.761840843,15.048546905,8.805440428,5.509873332,
```

```
                      6.383788955)
data.monitor[9,] = c(23.35137642,24.030529464,10.21770544,10.687368488,0.44753337,3.587244797,
                      27.678242025)
data.monitor[10,] = c(6.707293703,11.045954543,17.140009965,19.160889772,14.103368978,
                       5.962602047,25.879880995)
data.monitor[11,] = c(24.34703431,16.782232211,9.874689448,13.308284991,8.781559747,
                       4.936950053,21.969249248)
data.monitor[12,] = c(11.047557634,12.758246632,14.949264224,11.12449189,4.727633917,
                       4.341602303,41.0512034)
data.monitor[13,] = c(27.393757396,8.178575462,7.525441124,8.060374817,0.085485208,
                       3.331480658,45.424885337)
data.monitor[14,] = c(27.37635134,8.719733995,5.926471332,12.978616108,14.761634827,
                       7.060341039,23.176851367)
data.monitor[15,] = c(4.153251191,12.323417866,12.534571951,15.340602428,10.871998897,
                       4.915501805,39.860655861)
data.monitor[16,] = c(27.33568307,32.144871149,9.537147947,15.416120207,6.426941136,
                       3.587549189,5.551687301)
data.monitor[17,] = c(12.81311773,17.278589716,11.993259666,28.156607995,7.209725526,
                       4.265704656,18.282994719)
data.monitor[18,] = c(18.451604423,19.504529514,17.227306707,25.471119907,8.33872314,
                       5.274480225,5.732236081)
data.monitor[19,] = c(20.221870452,5.623254346,3.924602163,6.50836136,0.001274629,3.778638939,
                       59.941998108)
data.monitor[20,] = c(29.532304504,20.168195255,11.370704087,22.495108031,8.017640017,
                       3.650639954,4.765408148)
data.monitor[21,] = c(29.577397449,9.640577903,11.427696768,14.251611483,8.393395226,
                       4.48981702,22.21950415)
data.monitor[22,] = c(13.013623273,7.590026521,8.324706988,11.193220479,1.495807874,
                       6.604788172,51.777826693)
data.monitor[23,] = c(6.345988731,10.837452358,7.716923033,10.399483331,0.770586523,
                       6.677122514,57.252443511)
data.monitor[24,] = c(17.955167981,21.1284402,14.124135876,20.518986567,7.767013248,
                       2.754595562,15.751660575)
data.monitor[25,] = c(17.995944436,15.909370135,12.298847768,11.820340908,7.066666769,
                       4.748900842,30.159929137)
data.monitor[26,] = c(0,9.036038345,9.54968309,34.613492038,1.33289973,4.853290095,
                       40.614596699)
data.monitor[27,] = c(24.596803412,14.955729805,13.896628949,26.855790773,6.797435502,
                       4.524589171,8.373022386)
data.monitor[28,] = c(55.7629684,17.633905065,4.846217345,11.07136856,3.982786021,1.955057999,
                       4.747696603)
data.monitor[29,] = c(22.20709152,19.413898108,16.871530266,27.77748287,7.151435944,2.32872788,
                       4.249833411)
data.monitor[30,] = c(21.315956189,9.624942713,11.358686465,20.803083184,3.620279865,5.037392472,
                       28.239659106)
data.monitor[31,] = c(40.09,13.44,6.82,12.36,0.23,0.89,26.14)
data.monitor[32,] = c(27.45,8.3,6.91,10.97,10.45,5.48,30.45)
data.monitor[33,] = c(17.82,14.42,11.93,23.77,11.59,2.34,18.14)
data.monitor[34,] = c(32.19,20.38,10.33,15.71,7.81,2.81,10.77)
data.monitor[35,] = c(8.14,14.66,10.94,16.69,2.41,0.96,46.21)
data.monitor[36,] = c(26.65,15.38,14.14,27.17,8.28,3.9,4.48)
data.monitor[37,] = c(47.87,16.84,8.93,14.4,4.42,3.35,4.2)
data.monitor[38,] = c(12.53,26.2,10.61,24.72,3.51,3.68,18.76)
data.monitor[39,] = c(16.21,9.18,7.14,12.96,4.63,3.3,46.59)
data.monitor[40,] = c(15.53,12.04,6.7,16.29,1.22,1.82,46.4)
data.monitor[41,] = c(14.714170562,21.168269326,8.748796211,29.969961589,5.082002966,
                       4.631861606,15.684937748)
data.monitor[42,] = c(50.21930825,12.515007243,10.864294729,14.709940385,3.658049303,
                       4.007349396,4.026050695)
data.monitor[43,] = c(34.27991468,20.309884878,7.457288778,18.077038839,8.473922488,
                       6.963261065,4.438689264)
data.monitor[44,] = c(8.875709392,12.79717777,16.028426811,31.775653151,0,2.30235237,
                       28.220680503)
data.monitor[45,] = c(38.352100875,12.00967847,9.05844307,21.976417992,3.170064259,
                       4.740875135,10.692420192)
data.monitor[46,] = c(27.7977994,11.851379365,9.389252114,19.459068482,9.781070172,
                       6.468322509,15.253107969)
data.monitor[47,] = c(13.09210865,15.573939487,10.038142364,26.036081677,9.591681902,
                       5.184518476,20.483527443)
data.monitor[48,] = c(18.22499893,7.17831315,7.822328726,22.774420008,13.80709846,8.398506621,
                       21.794334101)
data.monitor[49,] = c(11.365452285,10.130373171,12.14948535,12.193379092,0,1.929245158,
                       52.232064943)
data.monitor[50,] = c(19.66421955,11.001820004,11.398539907,16.113780413,7.709821333,
                       6.242993824,27.868824973)
data.monitor[51,] = c(14.811358485,36.14718038,12.872950274,20.32063876,7.287831074,
```

```
                     4.239102911,4.320938119)
data.monitor[52,] = c(23.278824405,24.10250212,15.159468841,19.869704654,7.630631547,
                     5.077835411,4.881033028)
data.monitor[53,] = c(5.037953624,8.843714256,14.89809712,29.633122178,6.965435687,7.813018301,
                     26.808658834)
data.monitor[54,] = c(24.239301402,18.240494658,9.415848566,27.5653309,8.458932909,6.112961435,
                     5.967130133)
data.monitor[55,] = c(30.243519249,11.66012045,6.276512176,23.90933978,9.662500995,7.923603404,
                     10.324403949)
data.monitor[56,] = c(33.34046926,9.939630074,8.488182907,17.433419676,7.828502669,5.247050574,
                     17.722744843)
data.monitor[57,] = c(17.423996186,13.070705758,13.371904049,22.189782731,10.149556083,
                     6.146539384,17.647515812)
data.monitor[58,] = c(24.53199522,20.107505162,12.650499464,15.620035967,11.506600996,
                     5.696678802,9.886684393)
data.monitor[59,] = c(0,15.046443781,8.014486435,17.370998505,5.924600631,8.611433962,
                     45.032036685)
data.monitor[60,] = c(6.965118027,13.749812057,10.645015787,23.010869802,2.412456046,
                     5.234051596,37.982676685)
data.monitor[61,] = c(22.71476913,11.616315752,9.412332751,38.281419097,16.002505522,
                     1.336148398,0.63650936)
data.monitor[62,] = c(9.860083663,1.693446304,10.157701643,54.495477049,21.582307067,
                     1.530212128,0.680772141)
data.monitor[63,] = c(0,0.453901001,2.830506241,86.29922422,9.707112525,0.461475641,
                     0.247780375)
data.monitor[64,] = c(48.175906465,12.719053011,7.714793513,4.55230273,10.242158079,
                     8.682343179,7.913443026)
data.monitor[65,] = c(0,2.0912437,12.806882303,63.321224349,20.624491538,0.816156349,
                     0.340001761)
data.monitor[66,] = c(0,0,12.693575782,72.94952293,13.463463388,0.617602407,0.275835496)
data.monitor[67,] = c(0,1.102769159,5.605470531,77.980609639,14.457147904,0.630234211,
                     0.223768552)
data.monitor[68,] = c(41.15613086,16.242988024,6.810621798,1.979931403,9.6392281,13.777570855,
                     10.393528972)
data.monitor[69,] = c(0,0.686524744,12.070305768,68.224652036,17.025392863,1.351802827,
                     0.641321762)
data.monitor[70,] = c(0,0.064210164,4.953695259,64.42670226,29.278610595,0.764480507,
                     0.512301224)
data.monitor[71,] = c(0,0.456978438,12.224173224,81.65947079,5.023841052,0.392358781,
                     0.243177721)
data.monitor[72,] = c(15.838886376,6.505872572,7.88841359,38.474799508,28.220423815,
                     1.982872737,1.088731401)
data.monitor[73,] = c(17.791103613,10.255680113,11.512626273,23.258074576,13.789143373,
                     6.129846267,17.263525788)
data.monitor[74,] = c(0,4.385572169,16.064115914,58.95742159,18.573904955,1.361199175,
                     0.657786194)
data.monitor[75,] = c(0,0.253065093,7.8945306,72.091068243,19.301190706,0.460145369,0)
data.monitor[76,] = c(17.49890546,10.690776911,15.856246536,34.827312277,19.513646778,
                     1.044026872,0.569085168)
data.monitor[77,] = c(8.643730396,8.844030745,14.02872821,44.232026135,21.042339845,
                     1.878427256,1.330717414)
data.monitor[78,] = c(0.127054591,0.723658757,10.963522232,60.728936292,24.942371772,
                     1.001253446,1.513202906)
data.monitor[79,] = c(0.460620506,0.794173287,10.878409193,66.679055964,19.960134392,
                     0.704167968,0.523438697)
data.monitor[80,] = c(19.969184885,11.170703511,11.911628259,24.0643006,28.28903625,
                     3.101414716,1.493731781)
data.monitor[81,] = c(18.829641144,15.092235815,16.287481128,28.053245095,13.716187008,
                     2.233370199,5.787839611)
data.monitor[82,] = c(34.916973262,12.885055769,12.935984843,18.065463,12.517403875,
                     3.255972106,5.423147139)
data.monitor[83,] = c(0,0,2.888275537,83.200377647,13.911346814,0,0)
data.monitor[84,] = c(18.845435583,8.9331423,13.915861298,37.209559358,19.457174336,
                     1.638827117,0)
data.monitor[85,] = c(26.786033993,10.575256379,7.502489578,19.470972121,19.219667099,
                     6.936236927,9.509343899)
data.monitor[86,] = c(12.23484798,14.852271544,13.415902964,40.266867692,16.541425198,
                     1.559780783,1.128903839)
data.monitor[87,] = c(4.101526087,10.393859476,6.997383792,53.982182959,24.518355204,
                     0.006692474,0)
data.monitor[88,] = c(39.26997075,17.258009374,7.984894055,11.414748262,14.23538601,
                     4.420299385,5.416692156)
data.monitor[89,] = c(0,0.744870488,17.601332685,71.27285406,9.1124969,0.548727898,
                     0.719717976)
data.monitor[90,] = c(10.47234374,22.662352034,16.484169645,29.741772311,18.389994871,
                     1.674546078,0.574821322)
```

```
data.monitor[91,] = c(31.51,33.47,9.57,8.63,10.11,3.14,3.57)
data.monitor[92,] = c(0,0.23,5.71,77.59,16.38,0.08,0)
data.monitor[93,] = c(44.54,23.86,4.98,4.61,0.84,0.49,20.68)
data.monitor[94,] = c(28.64,10.71,12.21,31.99,12.36,1.96,2.13)
data.monitor[95,] = c(0,11.49,25.03,52.95,9.46,0.67,0.4)
data.monitor[96,] = c(56.19,5.29,5.41,14.12,9.32,4.76,4.92)
data.monitor[97,] = c(27.99,3.35,14.05,39.15,7.73,4.32,3.42)
data.monitor[98,] = c(12.3,8.58,9.89,26.53,29.9,5.83,6.97)
data.monitor[99,] = c(0,0.07,15.66,76.64,6.69,0.17,0.78)
data.monitor[100,] = c(28.05,13.26,10.91,14.27,20.36,5.6,7.53)
data.monitor[101,] = c(9.307060133,4.573773867,11.488148027,13.175242236,2.35364144,
                       11.172752821,47.929381473)
data.monitor[102,] = c(30.55597801,21.21146283,12.672996285,8.431460122,12.338135268,
                       8.733460816,6.056506659)
data.monitor[103,] = c(29.62771791,26.777534036,9.229627718,15.307375097,12.387699502,
                       4.567613354,2.102432378)
data.monitor[104,] = c(11.25420144,3.470674874,18.157785416,53.95715624,11.555393377,
                       1.604788651,0)
data.monitor[105,] = c(20.321552157,10.906333839,9.811812278,29.86476901,18.670105228,
                       6.67446194,3.750965549)
data.monitor[106,] = c(17.505266141,4.051561424,6.436148253,12.134692611,28.803509444,
                       27.162130713,3.906691411)
data.monitor[107,] = c(19.937172279,3.566535973,14.462185271,28.272940291,22.67371029,
                       6.626955604,4.460500288)
data.monitor[108,] = c(8.14104061,30.46256395,12.698749655,8.337816121,23.230225405,
                       12.170782687,4.958821577)
data.monitor[109,] = c(32.055347366,8.890993699,11.921920685,12.374762345,21.706550465,
                       8.295712906,4.754712536)
data.monitor[110,] = c(27.42527379,17.521262975,18.633701268,18.559567489,6.287301176,
                       3.623919272,7.948974032)
data.monitor[111,] = c(19.818792162,12.062053906,8.84202979,11.178456449,7.391884839,
                       8.005033032,32.70174982)
data.monitor[112,] = c(42.86266665,18.201839177,6.783450734,11.846192524,10.753279656,
                       5.327124808,4.225446453)
data.monitor[113,] = c(23.959250774,17.344841754,7.410017028,13.201405232,17.196514664,
                       12.524268981,8.36370156)
data.monitor[114,] = c(32.524442305,18.646385865,7.760548543,9.942942586,16.206984295,
                       8.723622759,6.195073645)
data.monitor[115,] = c(38.533385949,13.76782996,11.422450246,18.279606701,10.386246154,
                       3.808602438,3.801878548)
data.monitor[116,] = c(28.573592421,17.808456315,15.116631324,15.667161222,11.239338673,
                       4.729723911,6.865096143)
data.monitor[117,] = c(2.357646825,7.68657458,7.222001392,24.151413042,30.73491074,17.95071099,
                       9.896742425)
data.monitor[118,] = c(12.23989367,16.839306998,15.407003392,23.556032199,20.7117997,
                       7.756356462,3.489607588)
data.monitor[119,] = c(16.401357522,27.845339587,22.213243909,15.372948488,8.733404387,
                       6.231739001,3.201967112)
data.monitor[120,] = c(7.088228774,19.341609394,13.208453411,21.994976823,20.30710953,
                       10.732908608,7.326713461)

## Run test
nmons = dim(data.monitor)[1]
ps = rep(0, nmons)

for (j in 1:nmons) {
  ps[j] = quantile(data.monitor[j,], mu, sigma)
}

## Pick off the ones that fail. FALSE means the sample is OK (i.e. acceptable sediment
# composition)
fail = ps<0.05
fail

## Add results of Mahalanobis tests to df 'b'
b2 <- cbind(b, as.data.frame(fail))
View(b2)

## Create a col for Year/Treatment
b2$TreatYear=NA

## Populate new column 'TreatYear' by concatenating cols 'Treatment' and 'Year'
b2$TreatYear <- do.call(paste, c(b2[c("Treatment", "Year")], sep = "_"))

## Identify the number of Mahalanobis fails by Treatment/Year group
library(plyr)
```

```
b2failsum=ddply(b2,~TreatYear,summarise,sum=sum(fail))
b2failsum # Enter these results in next step

## Save df 'b2' as a .csv.
write.csv(b2,"OUTPUTS/Mahalanobis test results for Area 222 recovery time series.csv")

## Now use data from above file to identify problem sediment fractions. Follow approach in Table
# 4 of the paper, subtracting distribution means from test means etc.

###################################################################################################
### STEP:              68                                                                       ###
###                                                                                             ###
### PAPER SECTION:     METHODS \ Assessing the ecological significance of sediment change       ###
###                    RESULTS \ Assessing the ecological significance of sediment change       ###
###                                                                                             ###
###                                                                                             ###
### TASK:              Supplementary Figure S2 (Area 222 Mahalanobis Calcs)                     ###
###                                                                                             ###
###################################################################################################

## Results from Mahalanobis tests of Area 222 sediment data (Reference and Dredged (High) sites)
Area222data2=data.frame(year=c(5,6,7,8,11,15,5,6,7,8,11,15),
                        failure=c(70,50,30,30,0,0,0,0,0,0,0,10),
                        Treatment=c("Dredged","Dredged","Dredged","Dredged","Dredged","Dredged",
                                    "Reference","Reference","Reference","Reference","Reference",
                                    "Reference"))
View(Area222data2)
str(Area222data2)

library(ggplot2)
# Basic barplot
p<-ggplot(data=Area222data2, aes(x=year, y=failure,fill=Treatment,colour=Treatment)) +
  geom_bar(stat="identity",position="dodge")+
  ylab("%")+
  xlab("Years after dredging")+
  ylim(0,100)+
  scale_x_continuous(breaks=seq(4,15,by=1))+
  annotate("text", label = "Faunal recovery \n (Dredged site)", x = 12, y = 20,
           color = "black",size=4)+
  geom_segment(aes(x=13,y=16,xend=14.75,yend=1),size=0.5,colour="black",
               arrow = arrow(length = unit(0.3,"cm"))) +
  theme_bw(base_size=14)
p

## Save plot to an image file (png or tiff)
png("OUTPUTS/FIGURE S2.png",width = 15,height = 15,units = "cm",res = 800,pointsize = 14)
#tiff("OUTPUTS/FIGURE S2.tiff",width = 15,height = 15,units = "cm",res = 800,pointsize = 14)
p
dev.off()
```
